# p70S6K/Akt dual inhibitor DIACC3010 is efficacious in preclinical models of gastric cancer alone and in combination with trastuzumab

**DOI:** 10.1038/s41598-023-40612-9

**Published:** 2023-09-25

**Authors:** Shota Fukuoka, Yoshikatsu Koga, Mayumi Yamauchi, Shigehiro Koganemaru, Masahiro Yasunaga, Kohei Shitara, Toshihiko Doi, Takayuki Yoshino, Toshio Kuronita, Brian Elenbaas, Pamela Wahra, Hong Zhang, Lindsey Crowley, Molly H. Jenkins, Anderson Clark, Takashi Kojima

**Affiliations:** 1 Division of Experimental Therapeutics, Exploratory Oncology Research and Clinical Trial Center, National Cancer Center, Kashiwa, Japan; 2 Department of Gastroenterology and Gastrointestinal Oncology, National Cancer Center Hospital East, 6-5-1, Kashiwanoha, Kashiwa, Chiba 277-8577 Japan; 3 Division of Developmental Therapeutics, Exploratory Oncology Research & Clinical Trial Center, National Cancer Center Hospital, Kashiwa, Japan; 4 Department of Experimental Therapeutics, National Cancer Center Hospital East, Kashiwa, Japan; 5 Merck Biopharma Co., Ltd. (an affiliate of Merck KGaA), Tokyo, Japan; 6 EMD Serono Research & Development Institute, Inc. (an affiliate of Merck KGaA), Billerica, MA USA

**Keywords:** Cancer, Gastrointestinal cancer

## Abstract

The PI3K-Akt-mTOR (PAM) pathway is implicated in tumor progression in many tumor types, including metastatic gastric cancer (GC). The initial promise of PAM inhibitors has been unrealized in the clinic, presumably due, in part, to the up-regulation of Akt signaling that occurs when the pathway is inhibited. Here we present that DIACC3010 (formerly M2698), an inhibitor of two nodes in the PAM pathway, p70S6K and Akt 1/3, blocks the pathway in in vitro and in vivo preclinical models of GC while providing a mechanism that inhibits signaling from subsequent Akt up-regulation. Utilizing GC cell lines and xenograft models, we identified potential markers of DIACC3010-sensitivity in Her2-negative tumors, i.e., *PIK3CA* mutations, low basal pERK, and a group of differentially expressed genes (DEGs). The combination of DIACC3010 and trastuzumab was evaluated in Her2-positive cell lines and models. Potential biomarkers for the synergistic efficacy of the combination of DIACC3010 + trastuzumab also included DEGs as well as a lack of up-regulation of pERK. Of 27 GC patient-derived xenograft (PDX) models tested in BALB/c nu/nu mice, 59% were sensitive to DIACC3010 + trastuzumab. Of the 21 HER2-negative PDX models, DIACC3010 significantly inhibited the growth of 38%. Altogether, these results provide a path forward to validate the potential biomarkers of DIACC3010 sensitivity in GC and support clinical evaluation of DIACC3010 monotherapy and combination with trastuzumab in patients with HER2- negative and positive advanced GCs, respectively.

## Introduction

Gastric cancer (GC) is the fifth most common malignancy and the third leading cause of cancer death worldwide^1^, with most GCs occurring in patients from Asia and South America^2^. For pre-metastatic, early-stage GCs, the current standards of care (surgery and chemotherapy) provide relatively good outcomes (5-year survival rates close to 90%). Refined chemotherapeutic treatments and the approval of targeted therapies, such as trastuzumab, ramucirumab^3^, pembrolizumab, and nivolumab^4,5^, have helped to improve GC patients’ outcomes. However, patients with late-stage, metastatic disease, which represents most cases due to its relatively late detection, still have poor prognoses^2^ with global 5-year survival rates below 20%^6^. Innate or acquired resistance is also a significant issue, reducing effectiveness of approved therapies in this disease. Undoubtedly, better treatment options are needed for GC patients.

PI3K/Akt/mTOR (PAM) pathway components have been explored as targets for GC, due to its involvement in both the origin and progression of GC^7^. The mTOR inhibitor everolimus showed promising results in a Phase II clinical trial^8^ but failed to provide significantly better outcomes than best supportive care in the GRANITE-1 Phase III trial^9^. Akt is another promising target for GC given the relatively high rate (78%)- of Akt activation in this disease^10^. However, neither pan-Akt inhibitors ipatasertib^11^ or MK-2206^12^ had significant activity in Phase II clinical trials of patients with GC.

The unrealized potential of PAM pathway inhibitors may be due, in part, to toxicity concerns and the potential hyperactivation of Akt isoforms occurring upon PAM pathway blockage^13^. A drug that overcomes these toxicities, while still blocking the PAM pathway, would be a promising candidate for GC treatment. DIACC3010 (formerly M2698) is a selective ATP-competitive dual inhibitor of p70S6K and Akt1/3, which can overcome the compensatory Akt feedback loop induced by PAM pathway inhibition, while avoiding the toxic effects of pan-Akt inhibition^14,15^. In a Phase I study (NCT01971515) in patients with advanced solid tumors, DIACC3010 was well tolerated with evidence of clinical benefit, most notably in patients whose tumors lacked confounding tumor alterations (*EGFR, KRAS, AKT2*) but harbored PAM pathway alterations^16^. It is noteworthy that no limiting hyperglycemia was observed in this clinical trial with DIACC3010 in contrast to that seen with other pan Akt inhibitors^17^.

In addition, given that trastuzumab is an approved drug for treating HER2+ GC and PAM pathway activation has been described as a marker of resistance to anti-HER2 therapy in GC, any investigation of the potential of DIACC3010 as a GC treatment should also include combination with trastuzumab.

Therefore, we characterized the potential of DIACC3010 to treat human GC by employing both in vitro and in vivo preclinical models and investigated how the preclinical efficacy of DIACC3010, alone and combined with trastuzumab, correlated with known (*PIK3CA* mutations^18^ and pERK expression^19^) and potential biomarkers of sensitivity and resistance to PAM pathway inhibition. Altogether, these results support further clinical evaluation of DIACC3010 in patients with advanced GCs, both alone in HER2-negative GC and in combination with anti-HER2 treatment in HER2-amplified GC.

## Materials and methods

### In vitro methods

#### Cell culture and proliferation assay

Thirteen GC cell lines were used in this study: IM95m, MKN-1, MKN-45, and NUGC-3 (Japanese Collection of Research Bioresources); GCIY, GSU, HGC-27, KE-39, MKN-7, and SH-10-TC (Riken Bioresources Center); NCI-N87 (American Type Culture Collection); OE-19 (European Collection of Authenticated Cell Cultures); and 44As3 (Luc, kindly provided by Dr K. Yanagihara, National Cancer Center Hospital East).

Cells were maintained in the medium recommended by suppliers, supplemented with 10% fetal bovine serum (Cell Culture Technologies), 100 units/mL penicillin, 100 μg/mL streptomycin, and 25 μg/mL amphotericin B (Sigma-Aldrich) in a humidified atmosphere containing 5% CO_2_ at 37 °C. The growth-inhibitory effects of DIACC3010, trastuzumab (Chugai Pharmaceutical Co., Tokyo), and the combination of these single agents were examined using a tetrazolium salt-based proliferation assay (WST-8 assay, Dojindo). Each cell line was seeded into 96-well plates (1.5 × 10^3^ cells/100 μL/well), incubated for 24 h (h) at 37 °C and then treated with DIACC3010 (0–10 μM) alone or with trastuzumab (0–10 μg/mL) for 72 h. Medium was removed and replaced with WST-8 solution (10 μL) and fresh medium (90 μL), and the plates were incubated for 2 h at 37 °C.

Growth-inhibitory effects were assessed spectrophotometrically with a SpectraMax 190 (Molecular Devices), and 50% growth inhibition (GI_50_) was determined from dose–response plots. Mean values and variances for each cell line were calculated for cell viability assays. Negative values shown in the dose–response plots indicate a decrease in cell count from baseline (Day 0, i.e., just before drug exposure). Bliss independence models were used to examine effects of DIACC3010 + trastuzumab. The Bliss expectation was calculated as (A + B) − A × B, where A and B were the fractional growth inhibitions induced by agents A and B at a given dose. Bliss sums were calculated for each cell line from the Bliss excess scores (difference between the Bliss expectation and observed growth inhibition induced by the combination of equal doses of agents A and B)^20^.

#### Biomarkers

Of the 13 GC cell lines, 7 were further investigated because of their potential use in xenograft models (HGC-27, IM95m, KE-39, NCI-N87, MKN-1, MKN7, OE-19). These 7 cell lines (0.5–1.0 × 10^6^ cells/well) were treated in vitro with DIACC3010 (0 or 1 μM) for 2, 6, and 24 h to assess DIACC3010 monotherapy effects on chosen biomarkers. For the combination studies, OE-19 and NCI-N87 cells were treated with DIACC3010 (0 or 1 μM) and/or trastuzumab (0 or 10 μg/mL) for 24 h.

Protein biomarkers were detected by western blot. Primary antibodies used for western blots were obtained from Cell Signaling Technologies: HER2 (#4290), pHER2 (Tyr1221/1222; #2243), pan-Akt (#4685), PTEN (#9188), PRAS40 (#2691), pPRAS40 (Thr246; #2997), p70S6K (#2708), pP70S6K (Thr389; #9234), S6 (#2217), pS6 (Ser240/244; #2215), pAkt (Ser473; #4060), ERK1/2 (#4695) and pERK1/2 (Thr202/Tyr204; #4370). Secondary antibodies were HRP conjugated donkey anti-mouse IgG (#715-036-150) and donkey anti-rabbit IgG (#711-036-152), purchased from Jackson Immuno Research.

Cells were lysed in RIPA buffer (Wako) and equal amounts of extracted protein (2 μg) were separated by SDS-PAGE and transferred to PVDF membranes using a Trans-Blot Turbo transfer machine (Bio-Rad). Immunostaining of the membranes was performed using the iBind western System (Life Technology) according to the manufacturer's protocol. Primary antibodies and secondary antibodies were diluted to appropriate concentrations in iBind buffer. Antibodies were used at the following dilutions: against HER2 (1:500), phosphorylated HER2 (Tyr^1221^^/1222^) (1:200), Akt (1:200), PTEN (1:500), PRAS40 (1:500), phosphorylated-PRAS40 (1:500), P70S6 kinase (1:500), phosphorylated-P70S6 kinase (1:500), S6 (1:500), phosphorylated-S6 (1:500), phosphorylated-Akt (1:400), ERK1/2 (1:200) and phosphorylated-ERK1/2 (1:400) as indicated. Subsequently, membranes were washed with a tris-buffered saline containing 0.1% Tween 20, and proteins were visualized using ECL prime substrate (GE Healthcare). Western blots were imaged using the ChemiDoc XRS Plus System (Bio-Rad) and band images were quantified by Image J (https://imagej.nih.gov/ij). The exposure time for detecting the signal in individual western blots differed among the cell lines evaluated and was determined at the optimum exposure time, given that the intensity of the baseline signal of each marker varied among the cell lines.

#### Colony formation assays (CFA)

Cell survival was determined by inoculating 6-well plates with different GC cell lines (1.5–3 × 10^3^ cells/well). After 24 h, the medium was changed for wells containing DIACC3010 (0, 0.13 or 0.25 µM) and/or trastuzumab (1 µg/mL) and the cells were incubated in a humidified chamber (at 37 °C) for 10 days. Cells were washed with PBS, fixed with 4% paraformaldehyde, and stained with crystal violet in water for at least 2 h at room temperature before imaging. The area covered by the colonies was quantified with ColonyArea, as described by Guzmán et al.^21^.

#### Mutational analyses

DNA was extracted from cell lines using the QIAamp DNA Mini Kit (QIAGEN) and stored at − 20 °C. Mutational hotspots in 48 cancer related genes were characterized using the TruSeq Amplicon Cancer Panel (IIlumina; genes listed in Supplementary Table S3), according to the manufacturer’s instructions.

### In vivo methods

#### Efficacy and PD analyses in cell line xenograft models

HGC-27 and OE-19 (1 × 10^7^) cells suspended in 0.1 or 0.2 mL PBS, respectively, were injected subcutaneously into the right flanks of 5-week-old BALB/c nu/nu mice. BALB/c nu/nu mice were obtained from Charles River Laboratories, Japan. Mice were maintained under specific pathogen-free conditions and provided with standard feed and sterilized water. Mice were monitored daily, and tumor volume (TV) was measured once or twice weekly using calipers; TVs were calculated as length/2 × width^2^. Endpoints calculated at the end of each study were tumor/control (T/C) and tumor growth inhibition (TGI); $\% {\text{T}}/{\text{C}} = \left[ {\left( {{\overline{\text{x}}\text{TV}}_{t} /{\overline{\text{x}}\text{TV}}_{c} } \right) \times 100} \right]$ and %TGI = [1-(TVΔ*t*/TVΔ*c*)], where *t* is treated and *c* is control.

When TVs reached approximately 200 mm^3^, mice from each model were assigned to four groups (n = 6). Mice with HGC-27 tumors were treated orally (po) with 10, 20, or 30 mg/kg DIACC3010 daily (QD) × 14 (10 mL/kg) and mice with OE-19 tumors were treated with DIACC3010 30 mg/kg po QD × 21, trastuzumab 15 mg/kg/week by intravenous injection (QW i.v.), or DIACC3010 + trastuzumab. Control mice were treated with the vehicle for DIACC3010 QD po (0.5% HPMC; 0.25% TBS-T; pH3 in 100 mM citrate buffer).

In a separate group of mice implanted with the same tumor cohort, HGC-27 and OE-19 tumors were allowed to grow for 14 days before mice were assigned to treatment groups (average TV of approximately 300–400 mm^3^). For DIACC3010 monotherapy, mice with HGC-27 tumors were treated with vehicle or DIACC3010 in vehicle at 20 mg/kg po QD × 4. Mice bearing OE-19 tumors were treated with DIACC3010 (30 mg/kg po QD × 4) and trastuzumab in PBS (15 mg/kg i.v. × 1). Tumor samples were collected upon euthanasia at 4 and 24 h after the final DIACC3010 treatment (n = 3/time point).

#### In vivo PDX models and tumor analyses

Twenty-seven patient-derived xenograft (PDX) models (Shanghai ChemPartner Co., Ltd.) of GC were used. They were derived from male and female Chinese patients (28–75 years of age) with either poorly or moderately differentiated gastric adenocarcinoma, except for one patient who was classified as having a Signet ring cell carcinoma (Supplementary Table S1). Models were implanted in Nu/nu mice that were obtained from Vital River Laboratory Animal Technology Co. LTD (Beijing, China).

Prior cryopreserved tumor fragments at passages 1–9 were implanted subcutaneously into the right flank of nu/nu mice and then passaged 3–5 more times to generate enough tumor-bearing mice for study enrollment (Supplementary Table S1). When TVs reached 100–250 mm^3^, mice from each model were assigned to one of four groups and treated with vehicle control, DIACC3010 (20 mg/kg QD po, except for GAX059 and GAX066 models [30 mg/kg QD PO]), trastuzumab (15 mg/kg QW i.v.)^22^, or DIACC3010 + trastuzumab. TVs and body weights (Supplementary Table S2) were measured twice per week. Based on the pharmacokinetics of DIACC3010 in mouse^23^ and human^16^, the clinically relevant dose of DIACC3010 in mice was calculated to be roughly 30 mg/kg QD. However, this dose was not well-tolerated in these studies, as determined by body weight loss, so the dose was decreased to 20 mg/kg QD after the first two models were treated. The study was terminated when mean TV in the vehicle control group reached 1200 mm^3^, with a study duration of 17–60 days after treatment start, depending on the model.

Tumors from naïve, untreated mice of each PDX model were dissected when they had reached 400–600 mm^3^ in size and cut in half. One half was fixed in 10% formalin and processed for paraffin embedding (FFPE). The other half was cut into halves again and snap frozen in liquid nitrogen and stored at − 80 °C. HER2 status of the PDX models was assessed in FFPE mounted sections by immunohistochemistry (IHC; PATHWAY anti-HER2/neu [4B5] Rabbit Monoclonal Primary Antibody, Ventana Medical Systems), and scored from 0 to 3+. Models that scored 2+ or 3+ were investigated for *HER2* amplification FISH, using the PathVysion HER2 DNA Probe Kit (Abbott Laboratories).

Phosphorylated (p) and total (t) ERK protein was analyzed in snap frozen PDX tumor fragments from untreated animals. Tumor pieces were placed in individual Percellys 2 mL homogenizing tubes containing ceramic beads (Bertin Corp, Rockville, MD) with 500 µl 10 × RIPA buffer (Cell Signaling, Danvers MA) diluted to 1 × with water supplemented with phosphatase and protease inhibitors (PhosSTOP, Sigma-Aldrich, St. Louis, MO), Pierce Protease Inhibitor Mini Tablets (Thermo Fisher, Waltham, MA), and phenylmethanesulfonyl fluoride (PMSF, Sigma-Aldrich) at a working concentration of 100 µg/mL from a stock solution of 2.5 mg/mL dissolved in isopropyl alcohol. Tubes were placed in a Percellys 24 Bead Mill Homogenizer located within a 4°C cold room. Tumor fragments were homogenized 3 times for 15 s each at 5000 rpm followed by a 30 s pause and another homogenization 3 times for 15 s each at 5000 rpm. The lysates were transferred to 1.5 mL centrifuge tubes and centrifuged at 14,000*g* for 30 min at 4 °C. The supernatants were transferred again to new 1.5 mL centrifuge tubes, 1 µl benzonase (Pierce, Thermo Fisher) was added to each tube, and all tubes were sonicated for 20 s in a sonicating 37 °C water bath. An additional centrifugation was performed at 14,000*g* for 15 min at 4 °C, and lysates were transferred again to 1.5 mL centrifuge tubes and protein concentrations were measured by Pierce BCA assay (Thermo Fisher) according to manufacturer’s instructions, and each sample was diluted to a standard concentration of 0.5 mg/ mL. Lysates were then aliquoted (10 µl per well) into multiple 96-well plates and stored at − 20 °C until analysis.

Immunomodulatory analysis was performed following the ProteinSimple (South Wallingford, CT) Jess 2 Immunoassays RePlex Module protocol provided with the EZ standard pack 1 of 12-230 kDa kit. Tumor lysates were probed with pERK1/2 antibody (Cell Signaling Technology, catalog #4370) and tERK1/2 antibody (Cell Signaling Technology, catalog #4695), according to manufacturer’s protocol, at a dilution of 1:1000 per well. Data analysis was performed using the Compass for SW software version 5.0. Using the high dynamic range detection profile exposure, area results for pERK were divided by area results for tERK to yield a corrected area.

For mutational status, genomic DNA was isolated from snap frozen tumor fragments from untreated xenograft mice using the AllPrep DNA/RNA FFPE Kit (Qiagen); 20 to 27 mg of tissue was used as input for the DNA isolation. The genomic DNA concentrations were quantified using the fluorescent dye-based QubitTM double stranded DNA HS Assay Kit (Thermo Fisher Scientific). Library preparation from 30 ng DNA per sample was performed with the Illumina AmpliSeq® Cancer HotSpot Panel v2 (genes listed in Supplementary Table S4) and the MiSeq next generation sequencing system according to the manufacturer´s protocols. Variant Lists included all detected variants and their annotation to the ClinVar, COSMIC, and dbSNP databases and the consequences of the mutations were classified. Only variants classified as pathogenic or likely pathogenic are shown.

For gene expression characterization, total RNA was extracted from approximately 10–15 mg of frozen tumor tissue from untreated xenograft mice using the RNAqueous-4PCR Kit according to the manufacturer’s instructions (Thermo Fisher Scientific). Purified RNA was quantified using a Qubit® 3.0 Fluorometer (Thermo Fisher Scientific). Gene expression was analyzed in 50 ng input RNA using the PanCancer IO360 Panel (nanoString IO 360 V1.0); raw counts were corrected by subtracting the mean + 2 standard deviations (SDs) from negative controls and then normalizing against the geometric means of both positive controls and reference genes. Gene expression amplification was evaluated using the multiplex digital nCounter® platform and analyzed with nSolver v4.0 software (nanoString Technologies, Inc., Seattle, WA, USA). The lower limit of detection was set at 20 counts, therefore transcripts where more than 95% of samples had counts under 20 were excluded from subsequent analyses.

Gene expression normalization was performed relative to housekeeping genes using the GeoMean algorithm. Of the 20 housekeeping genes on the panel, those with the lowest standard deviations after normalization were used (SD < 0.45), employing the geNorm algorithm for each comparison. Mean squared error analysis was used to identify potential samples of low data quality; no samples were removed. All normalized data was then transformed on a log2 scale to undergo further analysis.

### Ethics

All animal studies using cell line xenograft models were approved by the Committee for Animal Experimentation of the National Cancer Center, Japan (approval No. K16-003). These guidelines meet the ethical standards required by law and comply with the guidelines for the use of experimental animals in Japan.

The PDX models were previously established at ChemPartner. At the time of model establishment tumor tissues were obtained in hospitals from patients with informed consent, in accordance with protocols approved by the hospital and ChemPartner Institutional Ethics Committees. PDX studies were conducted at ChemPartner in compliance with the guidelines for the care and use of animals established by the Institutional Animal Care and Use Committee at EMD Serono Research and Development Institute, Inc., Billerica, MA, USA, an affiliate of Merck KGaA.

All in vivo experiments were carried out in accordance with ARRIVE guidelines (Animal Research: Reporting of In Vivo Experiments).

### Statistical analyses

In vitro*:* Statistical significance was analyzed for R and *p* values between GI_50_ values of DIACC3010 and phosphor-protein biomarker expression in the 13 cell lines by linear regression. In colony forming assay (CFA), *p* values were calculated based on one-way analysis of variance (ANOVA) with Tukey HSD (honestly significant difference).

In vivo: In the efficacy and pharmacodynamic (PD) biomarker studies, statistical significance was analyzed using ANOVA followed by Tukey’s post-hoc pairwise comparisons for the OE-19 xenograft or Dunnett’s pairwise comparisons with controls for the HGC-27 tumor model. TV data from PDX models were log-transformed and analyzed by Repeated Measures Analysis of Covariance (RM-ANCOVA) with the starting tumor volume as the covariate, followed by Tukey’s post-hoc pairwise comparisons.

For statistical analysis of pERK/tERK protein, models were grouped according to treatment response, i.e., statistically significant treatment effects compared to vehicle. Group A: models that responded equally to DIACC3010 and its combination with trastuzumab; Group B: models that responded equally to trastuzumab and its combination with DIACC3010; Group C: models that responded equally to DIACC3010, trastuzumab, and the combination; Group D: models that only responded to the combination of DIACC3010 and trastuzumab; and Group E: models that did not respond to any of the treatments. Data from Group A were compared to those from combined Group B-D by unpaired t-test with Welch’s two-tailed correction.

The aforementioned statistical analyses were performed using IBM® SPSS® Statistics version 21, R version 2.1.0, or GraphPad PRISM version 9.1.2. Significance of α = 0.05 was used for all tests except PDX tumor volume data from efficacy studies, where α = 0.10.

All statistical analyses of nanoString data were performed on log2 transformed normalized counts. Differential expression analyses were carried out using nSolver4.0 and Advanced Analysis package 2.0 to determine differentially abundant transcripts with a preset threshold of statistical significance. To control for multiple testing, a corrected for multiple comparisons using Benjamini–Yekutieli FDR adjusted *p* value (i.e., false discovery rate (FDR) q-value) threshold of 0.05 was used for statistical significance.

## Results

### pERK protein and mutated PIK3CA may be markers of resistance and sensitivity to DIACC3010, respectively, in GC

**Figure 1:**
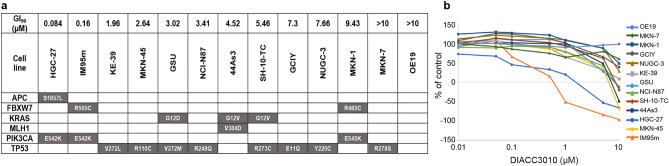
DIACC3010 inhibited growth in two out of three *PIK3CA* mutated GC lines, HGC-27 and IM95m. (**a**) Specific genetic mutations of 13 GC cell lines from the panel of 48 genes tested; only pathologic mutations are shown. In the top row of the table are the 50% growth inhibitory (GI_50_) values from the experiments shown in (**b**), where the growth-inhibitory effects of DIACC3010 were measured using a tetrazolium salt-based WST-8 proliferation assay. Cells were seeded into six 96-well plates and treated for 72 h (h) with DIACC3010 (0–10 μM; studies repeated 2–5 times).

Thirteen GC human cell lines were analyzed for 48 cancer-related mutational hotspots. Of the 13 lines, only HGC-27, IM95m, and MKN-1 harbored *PIK3CA* mutations and additional pathologic mutations (Fig. 1a).

In vitro, DIACC3010 only inhibited the antiproliferative activity of HGC-27 and IM95m (Fig. 1b), with 50% growth inhibition (GI_50_) values of 84 and 160 nM, respectively (Fig. 1a). The GI_50_ values of DIACC3010 in the other 11 cell lines, including that for MKN-1, were more than tenfold greater than that of the two most sensitive lines (Fig. 1a,b). An activating *PIK3CA* mutation may therefore confer sensitivity to DIACC3010, although it’s likely not the sole determinant considering that this mutation was also present in relatively resistant MKN-1 cells.

**Figure 2:**
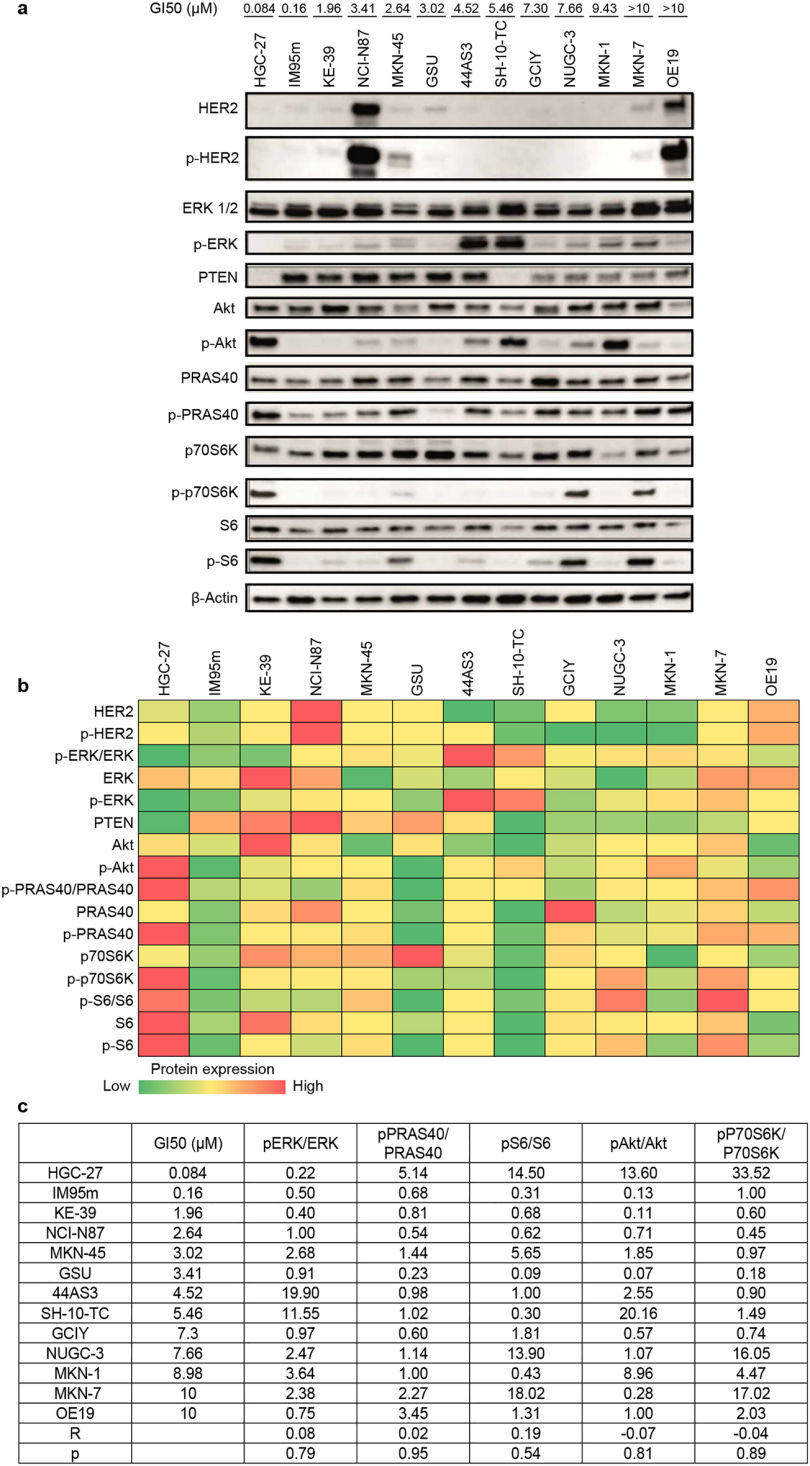
Two *PIK3CA*-mutated cell lines sensitive to growth inhibition by DIACC3010, HGC-27 and IM95m, had relatively low levels of pERK protein expression. (**a**) Thirteen GC cell lines were treated with DIACC3010 for 72 h (h) and then total cellular lysates were then immunoblotted, as indicated on the left. Black-edged rectangles delineate the blot. The 50% growth inhibition (GI_50_) sensitivity to DIACC3010 is shown in the top row. (**b**) Heat map depiction of band intensities of the western blots, where green and red represent relatively low and high protein expression, respectively. Relative protein expression was normalized to total protein (for phosphor-proteins) and to β-actin. (**c**) GI_50_ values versus phospho:total protein ratios for PAM pathway elements with corresponding correlation coefficients (R) and *p* values for the 13 GC cell lines. Statistical correlations were not significant (*p* > 0.05). Original blots are presented in Supplementary Fig. 9.

The basal levels of potential resistance markers were next examined through protein expression in the cells at steady-state via western blot analysis (Fig. 2a,b). The two cell lines sensitive to DIACC3010-induced growth inhibition, HGC-27 and IM95m, both had relatively low levels of pERK protein expression, while carrying *PIK3CA* mutations. The KE39 cell line also had low pERK expression but did not have the *PIK3CA* mutation, whereas the MKN-1 cell line carried the *PIK3CA* mutation but expressed relatively high pERK (Thr202/Tyr204) levels. Given that both MKN-1 and KE39 were resistant to DIACC3010, the presence of the putative sensitivity marker *PIK3CA*mut along with the absence of the potential resistance marker pERK may render GC cells susceptible to growth inhibition by DIACC3010.

Regression analysis of western blot band intensities versus GI_50_ values showed that expression of the markers examined at steady state, pERK/ERK, pPRAS40 (Thr246)/PRAS40 (a marker for Akt signaling), and pS6 (Ser240/244)/S6 (a substrate for p70S6K), did not correlate with the sensitivity of the cells to DIACC3010-mediated growth inhibition (all *p* > 0.05; Fig. 2c). Worth noting is that with only two highly sensitive cell lines out of the 13, achieving statistical significance with such an analysis is challenging.

### DIACC3010 inhibited the PAM pathway and blocked signaling of the induced Akt feedback loop

Inhibition of the PAM pathway is known to result in a compensatory feedback loop that augments signaling through Akt in cancer cells^13^. Stimulation of this feedback loop may reduce effectiveness of PAM pathway inhibitors that target at a single node, whereas the dual-node inhibition of DIACC3010 has been shown to overcome this limitation^14^.

**Figure 3:**
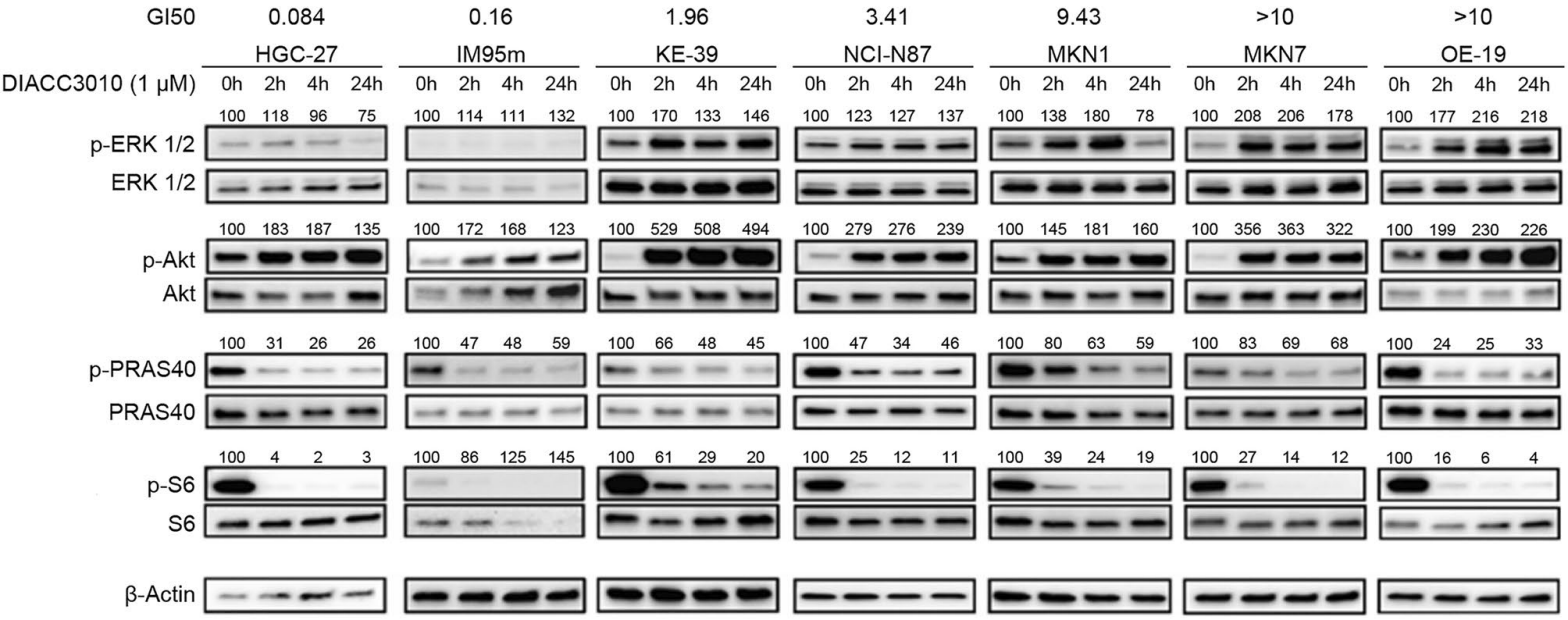
DIACC3010 affected expression of phosphorylated proteins of the PAM pathway in GC cells in vitro. Seven GC cell lines (top of panel) were treated with DIACC3010 for 0, 2, 6, or 24 h. Total cellular lysates were then immunoblotted, as indicated on the left. Black-edged rectangles delineate the blot. GI_50_ values for DIACC3010 in these GC cell lines compared with expression of phosphorylated and total biomarkers. β-actin was used as a loading control. *% of baseline (0 h) of expression levels of phospho-protein normalized by total level of the same protein are shown above the images of phospho-proteins. Original blots are presented in Supplementary Fig. 9.

To confirm these findings in GC cells, signaling components of the PI3K and MAPK pathways were evaluated in 7 of the 13 GC cell lines by western blot analysis. Cells were treated over time with DIACC3010 and represented relatively low, medium, or high GI_50_ values for DIACC3010, depending on the cell line (Fig. 3). DIACC3010 suppressed phosphorylation of S6 (decrease of pS6 band), the downstream marker of p70S6K, and increased pAkt (Ser473) in all lines, recapitulating the feedback loop mechanism. However, there were low basal levels of pS6 in the IM95m cell line and total expression of S6 was also affected by treatment. DIACC3010 also inhibited Akt signaling activity, as shown by the decreased expression of pPRAS40, a downstream marker of Akt. Notably, pERK (Thr202/Tyr204) expression increased in response to DIACC3010 in the five cell lines that were less sensitive to DIACC3010 but not in the two most sensitive cell lines, HGC-27 and IM95m (Fig. 3). Therefore, activation of the MAPK pathway is considered a potential resistance mechanism to DIACC3010 in GC.

### DIACC3010 inhibited growth of xenograft HGC-27 tumors and pS6 expression

**Figure 4:**
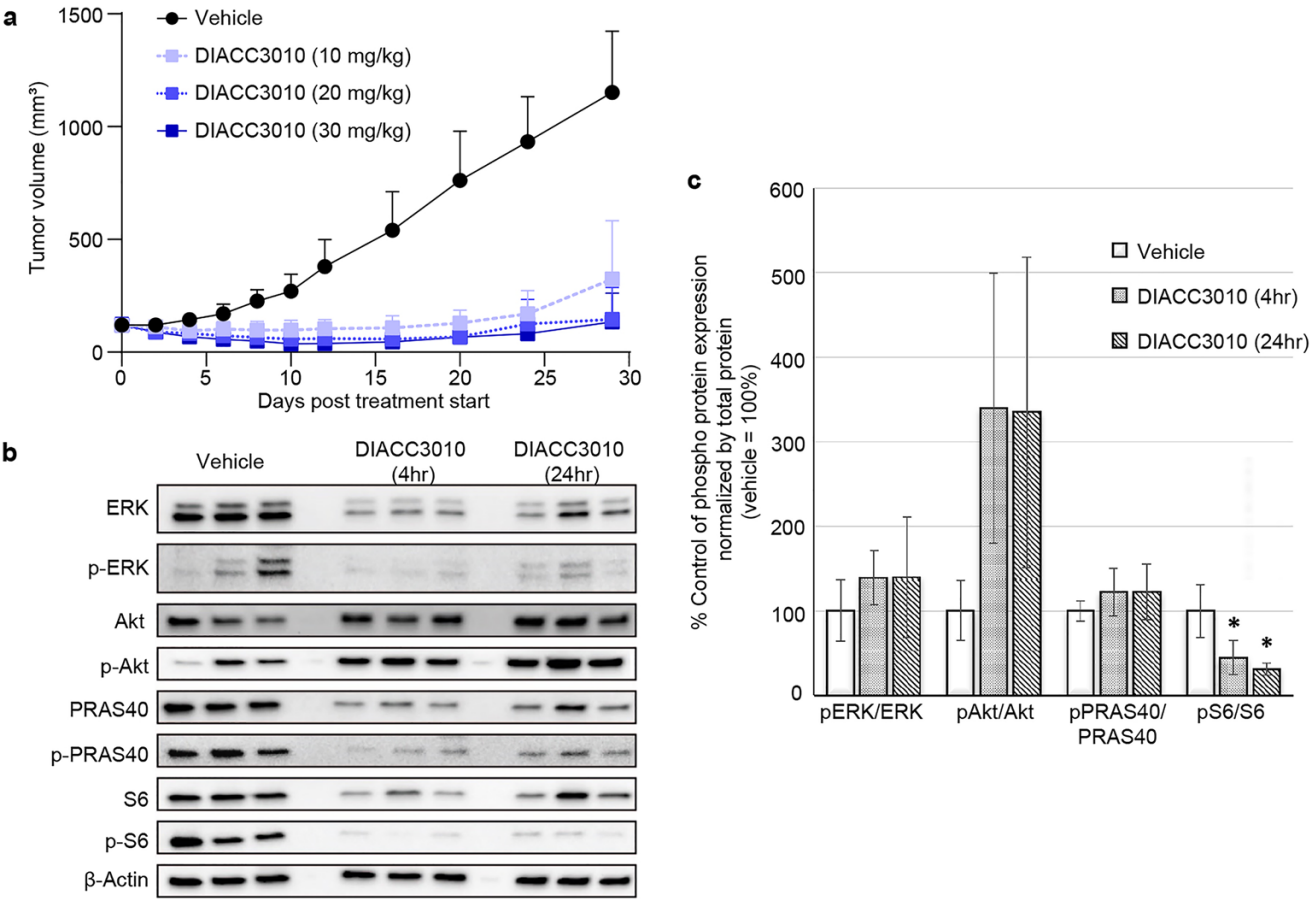
DIACC3010 inhibited growth of HGC-27 xenograft tumors in vivo and pharmacodynamically affected PAM pathway phospho-protein expression. Mice were injected subcutaneously in the flank with 1 × 10^7^ HGC-27 cells. (**a**) Once tumors reached approximately 200 mm^3^, mice were treated orally (po) with vehicle or DIACC3010 at 10, 20, or 30 mg/kg daily (QD; n = 7) starting at Day 0 for 14 days. DIACC3010 significantly inhibited tumor growth compared to vehicle at all 3 doses (*p < 0.01) at Day 29. Mean ± SD are shown. (**b**) In different mice of the same cohort, tumors were allowed to grow for 14 days (tumor size of approximately 300–400 mm^3^) and mice were treated with vehicle or DIACC3010 at 20 mg/kg po QD × 4. Tumor samples were collected upon euthanasia at 4 and 24 h after the final DIACC3010 treatment (n = 3/time point) and total tumor lysates were immunoblotted, as indicated on the left. Black-edged rectangles delineate the blot. (**c**) Band intensities of the western blots were quantified, and phospho-proteins were expressed as a ratio to total proteins (mean ± SD). pS6/S6 was significantly decreased at 4 h and 24 h compared to baseline (0 h; *p* < 0.05). Original blots are presented in Supplementary Fig. 9.

In the DIACC3010-sensitive HGC-27 cell line, all three DIACC3010 monotherapy doses tested (10, 20, and 30 mg/kg) resulted in significantly smaller tumors compared with vehicle control treatment, (*p* < 0.001, Fig. 4a), suggesting that maximum growth inhibition in this model was reached at the lowest treatment dose (10 mg/kg QD). There were no notable treatment effects on body weight in any of the dose groups (Supplementary Fig. S1).

The PD effects of DIACC3010 were also analyzed in xenograft HGC-27 tumors for expression of MAPK and PI3K signaling components (Fig. 4b,c). These results were confounded by the effects of treatment with DIACC3010 on all total proteins examined; however, some conclusions can be made. DIACC3010 significantly reduced pS6/S6 at both 4 h and 24 h relative to the vehicle control (Fig. 4c, p < 0.05), similar to what was seen in vitro (Fig. 3). Although the increase in pAkt/Akt seen in vitro was not significant in vivo, likely due to relatively high variation (*p* = 0.14), there appears to have been activation of the Akt feedback loop, which was confirmed when pAkt was normalized to β-actin (*p* = 0.04). Despite activation of pAkt, pPRAS40 was not increased, demonstrating the dual nature of DIACC3010 to inhibit the pathway at p70S6K and abrogate signaling through activated Akt. Neither pERK/β-actin nor pERK/total ERK ratios were affected by DIACC3010 (Fig. 4b,c), consistent with the in vitro results and the hypothesis that the sensitivity of these cells was due to a lack of pERK up-regulation, a potential resistance marker to PAM pathway inhibition in GC.

### DIACC3010 and trastuzumab combination therapy significantly inhibited growth of OE-19 tumors in mice relative to monotherapies

Given the importance of trastuzumab as an approved drug to treat HER-2 amplified GC and because PAM pathway activation has been described as a marker of resistance to trastuzumab in GC^24^, the combination of DIACC3010 and trastuzumab was examined. First, we tested whether DIACC3010 could reverse trastuzumab resistance in the GC cells. Of the 13 GC cell lines tested in this study, only OE19 and NCI-N87 cells expressed high levels of Her2 protein (Fig. 2a) and correspondingly, only growth of these two lines was inhibited by trastuzumab monotherapy in vitro (Supplementary Fig. S2) with GI_50_ values of 112 μg/mL and 0.8 μg/mL, respectively.

**Figure 5:**
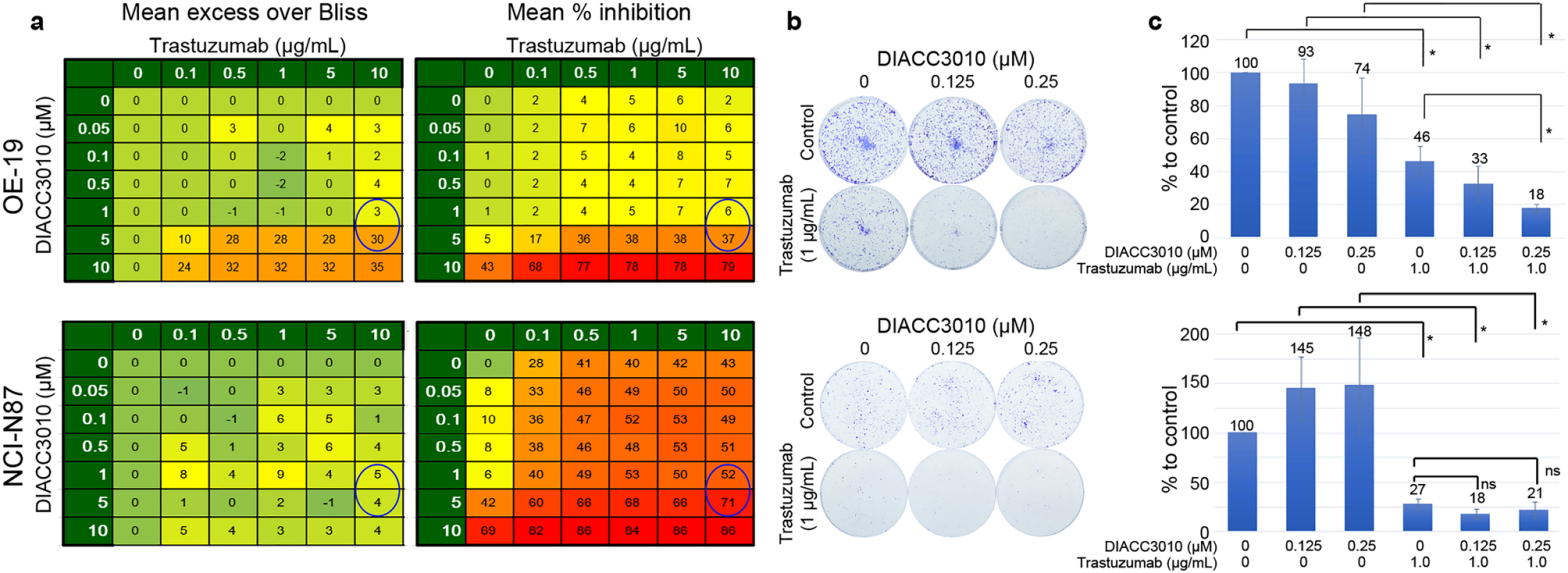
DIACC3010 and trastuzumab synergized to reduce cell growth and colony formation in OE-19 cells, but not in NCI-N87 cells. (**a**) Results of cell proliferation assays of DIACC3010 + trastuzumab using a Bliss independence model employing 6 × 7 dose matrices. The combination matrices were performed at least 3 times for OE-19 and NCI-N87 cell lines. Circled cells indicate scores at clinically relevant exposure of DIACC3010 and standard in vitro treatment dose of trastuzumab; (**b**) Visualization of colony formation assays (CFA) of cell lines with relatively low (NCI-N87) and high (OE-19) mean Bliss sums with varying levels of HER2 expression treated with DIACC3010 and trastuzumab. In agreement with the Bliss data, the only cell line exhibiting synergistic growth inhibition following treatment with the two agents was OE-19. (**c**) Quantitative data of CFA as mean % of control ± SD *p = 0.05. ns = not significant.

Next, the synergy of DIACC3010 and trastuzumab in combination on all 13 of the GC cell lines was examined with cell proliferation assays using a Bliss independence model employing 6 × 7 dose matrices. The combination matrices were performed 3–5 times for each cell line, resulting in mean Bliss sums from 295 for the OE-19 cells to -87 for the NUGC-3 cells (Table 1). Based on the variation of the scores and the majority of ranges that tended to center around zero, we concluded that true synergy of DIACC3010 + trastuzumab was associated with the consistently high Bliss sums in the replicate experiments of the OE-19 cells (Fig. 5a). Similarly, consistently negative Bliss sums were measured across the experiments of the NUGC-3 cells, indicating potential antagonism of the combination.

**Table 1:** Bliss sum values of DIACC3010 + trastuzumab combinations in vitro in GC cell lines.

Cell line	Mean (SD)					
	Bliss sum	n	Bliss Sum Range	% Inhibition DIACC3010 (1 μM)	% Inhibition trastuzumab (1 μg/mL)	HER2 expression^a^
OE19	295 (39)	4	235–339	1 (2)	6 (4)	50.83
KE-39	103 (132)	5	− 85 to 327	27 (9)	7 (5)	1.33
MKN-45	66 (63)	3	3–59	3 (3)	2 (1)	1.12
IM95m	41 (91)	4	− 48 to 126	62 (7)	6 (6)	0.58
44As3	35 (60)	4	− 21 to 120	4 (5)	2 (4)	0.27
HGC-27	32 (24)	5	− 6 to 52	73 (17)	1 (2)	0.85
SH-10-TC	23 (24)	3	− 3 to 27	6 (4)	2 (1)	0.42
MKN-7	11 (54)	5	− 51 to 84	12 (8)	3 (6)	3.00
MKN-1	− 13 (62)	4	− 88 to 65	5 (4)	2 (2)	0.49
GSU	− 24 (80)	3	− 110 to 48	15 (14)	14 (1)	2.12
NCI-N87	− 41 (117)	3	− 129 to 92	11 (8)	28 (11)	130.53
GCIY	− 77 (102)	5	− 200 to 48	17 (13)	3 (8)	1.00
NUGC-3	− 87 (57)	3	− 149 to 37	6 (2)	7 (4)	0.46

^a^Relative expression of HER2 to median value (indicated in GCIY cells) is shown. The original data (image) of HER2 expression is shown in Fig. 2a.

The highest Bliss score, and presumed combination synergy, in the HER2+ cell line OE-19 begged the question as to why the other HER2+ line, NCI-N87, had a relatively low Bliss score, i.e. no synergy (Table 1, Fig. 5a). The OE-19 cells responded to trastuzumab monotherapy in the WST-8 cell proliferation assay with a GI_50_ of roughly 112 μg/mL, which falls within the range of clinically relevant exposure in patients^25^. Thus, sub-clinical doses of trastuzumab in the Bliss matrix allowed for synergy to be detected when DIACC3010 was added, with the maximum of 10 μg/mL of trastuzumab being a relatively typical dose used for in vitro studies involving GC and breast cancer cell lines^26,27^. NCI-N87 cells were even more sensitive to trastuzumab in the WST-8 assay, with a GI_50_ of 0.8 μg/mL, and tended to show a lower Bliss score. The sensitivities of the two cell lines to trastuzumab appeared to have been related to their relative expression of HER2, which was greater in the more-sensitive NCI-N87 cells.

To test these presumptions from the Bliss analyses, cell lines of relatively low (NCI-N87), mid (MKN-7), and high (OE-19, KE-39) mean Bliss sums with varying levels of HER2 expression were tested in a colony forming assay (CFA) with DIACC3010 and trastuzumab. Overall, the CFA results supported the Bliss data; OE-19, but not NCI-N87, exhibited synergistic growth inhibition following treatment with DIACC3010 and trastuzumab (Fig. 5b,c, Supplementary Fig. S3).

**Figure 6:**
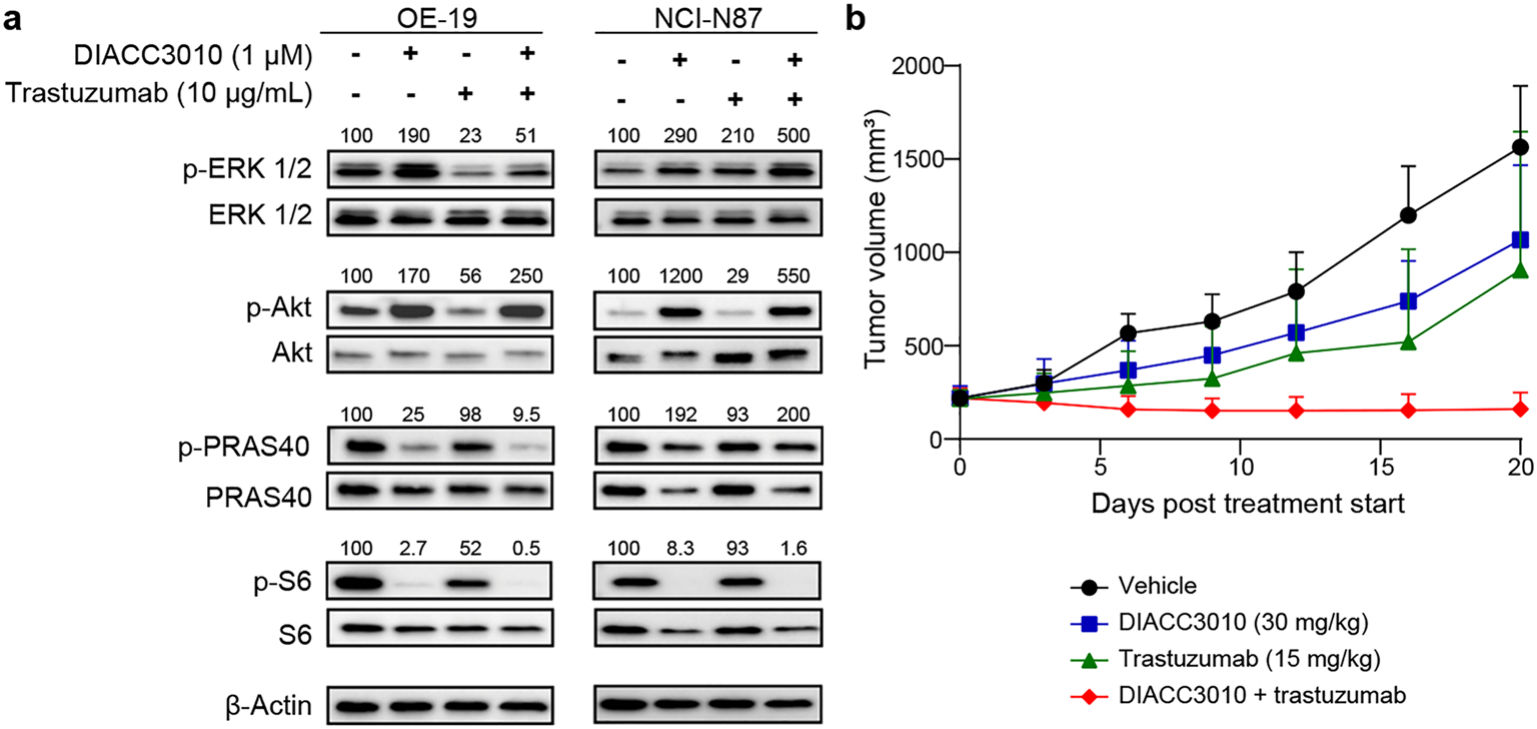
Synergy in OE-19 cells was associated with a combination-induced decrease in pERK and tumor growth inhibition in vivo. (**a**) OE-19 and NCI-N87 cells were treated with DIACC3010 and/or trastuzumab and total cellular lysates were immunoblotted, as indicated on the left. Black-edged rectangles delineate the blot. This western blot analysis revealed that OE-19 cells, which had a synergistic response to the DIACC3010 + trastuzumab combination, had a decrease in pERK/ERK following treatment with trastuzumab, either alone or combined with DIACC3010. NCI-N87 cells, which did not respond synergistically to the combination, had an increase in pERK/ERK following treatment with both monotherapies and a further increase in response to the combination. (**b**) Mice were injected subcutaneously in the flank with 1 × 10^7^ OE-19 cells. Once tumors reached approximately 200 mm^3^, mice were assigned to treatments (n = 6): vehicle, DIACC3010 at 30 mg/kg orally/daily (po, QD), trastuzumab 15 mg/kg once per week (iv, QW). The monotherapy treatments were not effective (*p* > 0.05), whereas the combination significantly inhibited tumor growth (*p < 0.01). Original blots are presented in Supplementary Fig. 9.

To investigate the signaling in OE-19 and NCI-N87 HER2+ lines that had disparate synergy results with the DIACC3010 + trastuzumab combination, the PD effects of DIACC3010 + trastuzumab combination were examined by western blot analysis (Fig. 6a). In both cell lines, DIACC3010 monotherapy and in combination with trastuzumab inhibited p70S6K, which stimulated the PAM pathway feedback loop, shown by the reduction in pS6/S6 and the increase in pAkt/Akt expression, while also blocking the signaling of Akt, as evidenced by the reduction in pPRAS40/PRAS40. The difference between the two cell lines was seen with pERK/ERK expression, which was decreased in OE-19 cells upon treatment with trastuzumab, both alone and in combination with DIACC3010, whereas pERK/ERK expression was increased in NCI-N87 cells upon monotherapy treatments and was augmented with the combination therapy (Fig. 6a). Therefore, the reduction of pERK (Thr202/Tyr204) induced by trastuzumab may have been sufficient for the cells to overcome resistance to DIACC3010. pERK may therefore be a candidate marker of synergism of DIACC3010 + trastuzumab treatment in HER2+ GC models.

Based on these cumulative results from the OE-19 cells in vitro, the synergy of DIACC3010 and trastuzumab was evaluated in this model in vivo. The combination of DIACC3010 + trastuzumab significantly inhibited tumor growth compared with vehicle and both monotherapies (all *p* < 0.05; Fig. 6b) in OE-19-tumor-bearing mice. Neither monotherapy significantly affected tumor growth (*p* > 0.05), thus confirming the synergy seen in vitro. We observed no notable effects of treatment on the body weight in any group (Supplementary Fig. S4).

### Sensitivity of GC PDX models to DIACC3010 and trastuzumab monotherapies or combination treatment could be linked to their mutational and/or HER2 status or differentially expressed genes (DEGs)

**Figure 7:**
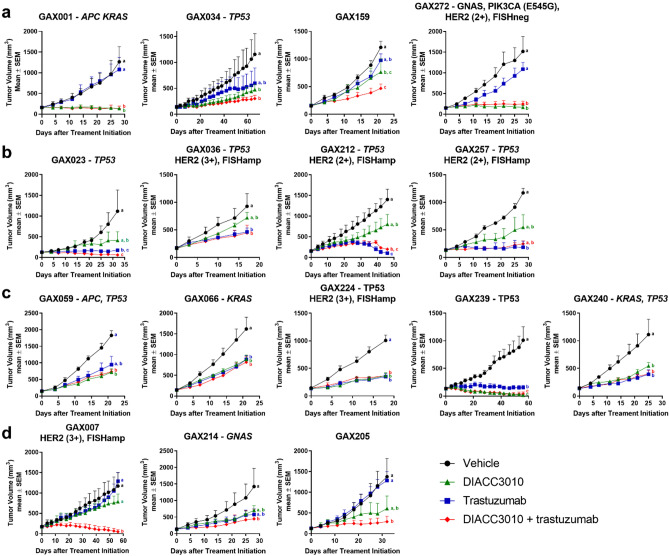
DIACC3010 and trastuzumab, alone and in combination, had differential effects in a panel of GC patient-derived xenograft models. (**a-d**) nu/nu mice bearing subcutaneous PDX tumors (100–250 mm^3^) were treated (n = 3 per group) with vehicle control, DIACC3010 (20 mg/kg QD po), trastuzumab (15 mg/kg QW iv), or a combination of DIACC3010 + trastuzumab, with the exception of GAX059 and GAX066 models, which received DIACC3010 at 30 mg/kg QD po. TVs and body weights were measured twice per week and the study was terminated when mean TV in the vehicle control group reached 1200 mm^3^. (**a**) Treatment with DIACC3010 alone and combined with trastuzumab significantly inhibited tumor growth compared with vehicle control in 4 models but these 2 treatments were not significantly different from each other. (**b**) Treatment with trastuzumab alone and combined with DIACC3010 significantly inhibited tumor growth compared to vehicle control in 3 models but these 2 treatments were not significantly different from each other. (**c**) Treatment with both agents alone and in combination all inhibited tumor growth equally compared to vehicle control in 5 models. (**d**) The combination of DIACC3010 + trastuzumab significantly inhibited tumor growth in 2 models compared to vehicle control, whereas effects of the monotherapies were not significant. In the GAX007 models, the combination effects on tumor growth were significantly different from the monotherapy effects. The model names, mutated oncogenic hotspot genes, and HER2 status are given above each graph. A lack of given HER2 status means the model was HER2 (1+) or (0). ^a,b,c^Tumor volume means of treatment groups for each model with similar superscripts were not significantly different (a = 0.10).

We assessed the effects of DIACC3010 and trastuzumab monotherapies and combination therapy in relation to the HER2 and mutational status in 27 GC PDX models derived from Chinese patient tumors. In the PDX models, sensitivity to a given treatment was determined by statistically significant differences compared with control. Overall, 9 of the 27 PDX models (33%) were sensitive to DIACC3010 monotherapy, 9/27 were sensitive to trastuzumab monotherapy (33%), and 16/27 (59%) were sensitive to the combination (Fig. 7, Supplementary Fig. S5). In the 21 models that were not *ERBB2-*amplified by FISH*,* 8 of them (38%) were sensitive to DIACC3010 monotherapy.

We looked at the relative sensitivities of the models to the different treatments and parsed the models into five groups. Four of the 27 PDX models (GAX001, GAX034, GAX159, GAX272 [15%]) were equally sensitive to DIACC3010 monotherapy and the combination (Group A; Fig. 7a). Four out of 27 PDX models (GAX023, GAX036, GAX212, GAX257 [15%]) were equally sensitive to trastuzumab monotherapy and the combination (Group B) and of those, GAX023 and GAX212 tumors regressed and GAX257 tumors experienced stasis (Fig. 7b). In Group C, five models (GAX059, GAX066, GAX224, GAX239, GAX240 [19%]) were sensitive to both monotherapies, but there were no additive or synergistic effects seen with the combination of DIACC3010 + trastuzumab, and only GAX239 experienced tumor regression (Fig. 7c). Three models (GAX007, GAX205, GAX214 [11%], Group D) were sensitive to DIACC3010 + trastuzumab combination therapy, but not to the monotherapies, where GAX007 exhibited what could be considered synergy (Fig. 7d), similar to what was seen with the OE-19 xenograft. The remaining 11 models [40%] were insensitive to all treatments (Group E; Supplementary Fig. S5).

Of the models sensitive to DIACC3010 (Group A), two had tumor growth inhibition that could be described as stasis (Fig. 7a), one of which harbored a *PIK3CA* mutation (GAX272). This was a similar rate of sensitivity to DIACC3010 as was seen in the cell line models (2 of 13 cell lines; 15%). Additionally, none of the four models that responded to DIACC3010 were *ERBB2-*amplified, as determined by FISH (Supplementary Table S1). In these Group A models, the combination treatment generated the same results as DIACC3010 monotherapy, and we concluded that trastuzumab did not add any benefit to the antitumor activity of DIACC3010 in those models. With GAX272 harboring the *PIK3CA* mutation, we considered mutated or amplified *PIK3CA* to be a potential biomarker of sensitivity to DIACC3010, analogous to the in vitro cell line data (Fig. 1). As a follow up, we conducted a preliminary analysis with tumor DNA from the three models most sensitive to DIACC3010 to look at *PIK3CA* copy number^28^, but the gene was not amplified (copy numbers were < 4). However, with 25% (1/4) of the Group A PDX models and both of the DIACC3010-sensitive cell line models harboring *PIK3CA* mutations, we consider this a possible response marker in a subset of tumors.

The sensitivity of models to DIACC3010, trastuzumab, and DIACC3010 + trastuzumab combination was compared with their HER2 status (Fig. 7, Supplementary Fig. S5). All the HER2 (3+) models and 33% (3/9) of the HER2 (2+) models had *HER2* amplification as determined by FISH (Supplementary Table S1), which was somewhat lower for HER2 (2+) than the 44% reported for the clinic^29^. The frequency of HER2+ models (HER2 (3+) or positive by FISH) was 22% (6/27), which was consistent with clinical findings^30^. Of the six *ERBB2*-amplified PDX models, four were sensitive to trastuzumab (67%), whereas none of the models that were sensitive to DIACC3010 were *ERBB2*-amplified and we considered that amplified *ERBB2* may be an exclusion marker for sensitivity to DIACC3010 in GC. Without a relatively high rate of response to DIACC3010 in the models that were not *ERBB2* amplified, we conducted several analyses of the untreated PDX tumors in an attempt to identify potential predictive markers of sensitivity to DIACC3010. First, sensitivity of the 27 GC PDX models to treatment with DIACC3010, trastuzumab or the combination was compared with mutational variants within these models, identified using the Illumina oncogene hotspot sequencing panel (Fig. 7, Supplementary Fig. S5, Supplementary Table S1). The main hotspot variants were for *TP53* (15/27 models, 55.5%), *KRAS* (5/27, 18.5%), and *APC* (3/27, 11%). These mutation frequencies are comparable to those observed in larger GC genomic databases (e.g. TCGA PanCancer Atlas, cBioPortal somatic mutation frequencies are: *TP53* 49%, *APC* 12%, *KRAS* 9%). Similar to what was seen for the cell lines, sensitivity to the treatments, whether monotherapy or the combination, could not be linked to the status of these mutations in the models. Based on the data from the cell line models, we then examined pERK protein in the PDX tumors. Variability in the pERK/tERK ratio across samples was relatively high (Supplementary Fig. S6). Comparing data from Group A (DIACC3010 sensitive) versus all other groups approached significance at *p* = 0.09. However, unlike the cell line models, there was no corresponding mutation of *PIK3CA* in three of four sensitive Group A models, as mentioned above.

Lastly, RNA from tumor samples were analyzed using the nanoString IO360 panel. This panel was designed to identify DEGs and gene signatures associated with tumor cell mechanisms, micro-environment, and immune response in human tumor samples. Limitations of the PDX model system would not allow full benefits of the panel because the diminished immune cell repertoire and much of the stromal component in the tumors would be of mouse origin, but with the tumor cells being human, information could be acquired for signaling, sensitivity and inhibitory mechanisms, tumor immunogenicity signals, and tumor metabolism. Analyses were focused on the models grouped A-E by responses to treatment as mentioned above.

For the results, no quality flags were observed so no sample exclusions in the data were necessary. As expected, some cell types were dropped from the analyses due to below-background reads, such as CD45 + , total tumor infiltrating lymphocytes, and Tregs. Initial unsupervised cluster analyses of normalized data revealed some clustering of genes which was not related to response group (Supplementary Fig. S7). For pathways, some clustering was seen for MAPK, especially with Groups B and D which appeared to be differentially expressed compared to Group E, and two major clades were seen for the PI3K-AKT pathway, but they were not enriched by group (Supplementary Fig. S7).

When looking at DEGs, comparisons were made between one response group and all other samples (e.g., Group A versus all others). Our main interest was whether we could identify potential predictive response markers for DIACC3010 (Group A) or for the combination (Group D), and whether we could identify potential resistance markers for the insensitive models (Group E). We only considered genes that were differentially expressed at Log2-fold or more and that were significant with an adjusted *p* value of 0.05 or less.

**Figure 8:**
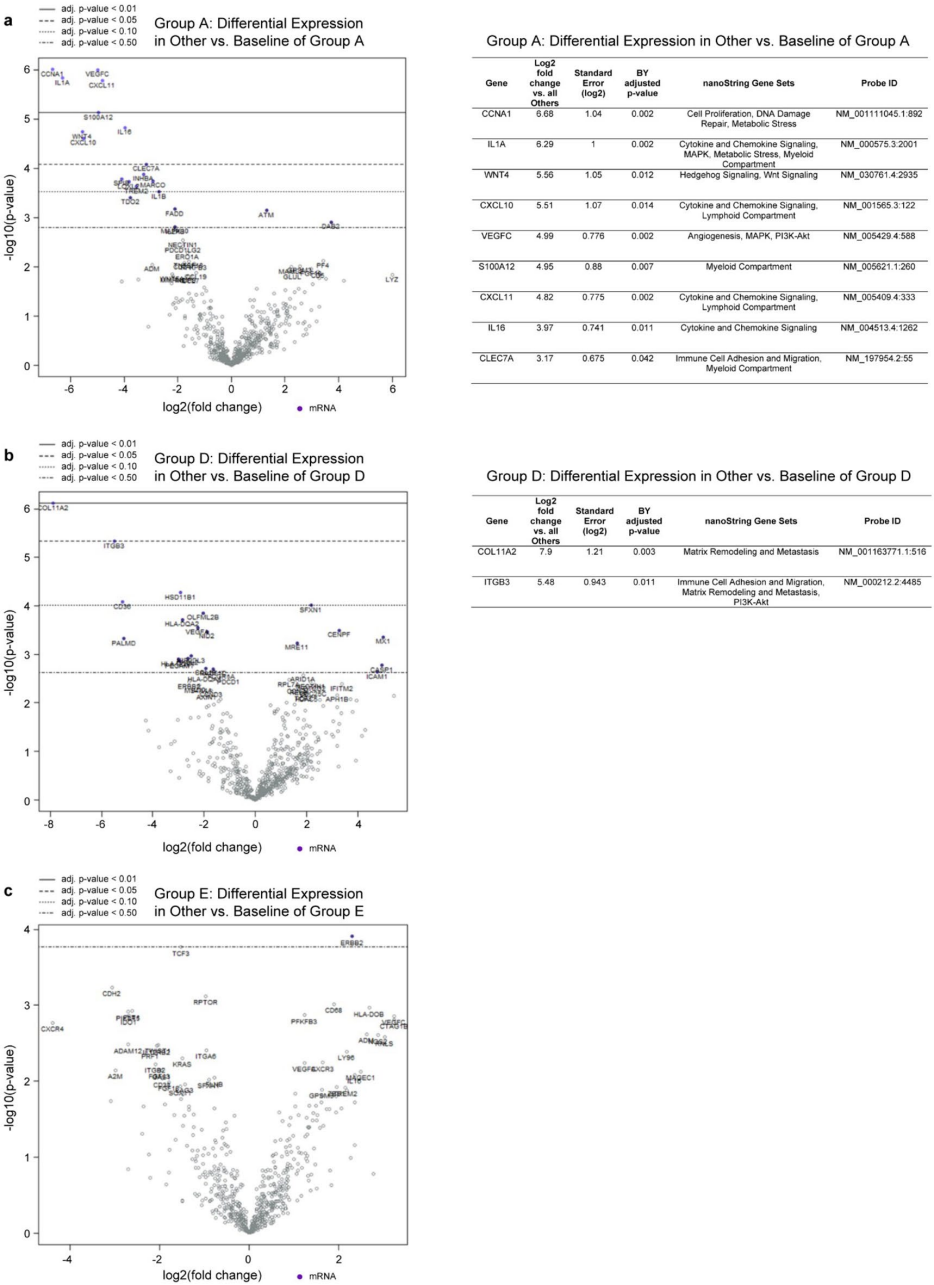
Volcano plots of relative expression of genes in the nanoString PanCancer IO 360 panel in GC PDX tumors. X-axes show lower (to the left of 0) or higher (to the right of 0) log2-fold change in expression of genes in all other tumors versus those in Group A (DIACC3010-sensitive; Panel [**a**]), Group D (combination-sensitive; Panel [**b**]) or Group E (resistant; Panel [**c**]). Y-axes show the -log10 unadjusted *p* value, and horizontal lines denote levels of Benjamini–Yekutieli (BY) corrected adjusted *p* values. Tables to the right of each plot list those genes differentially expressed by at least 2.0 log2-fold and statistically significant with an adjusted *p* value of < 0.05. Nine and 2 genes were significantly expressed by Group A models (**a**) and Group D models (**b**), respectively, at more than 2-(log2)-fold versus all other models. No genes with significant differential expression were detected in Group E versus all others (**c**).

The comparison with Group A yielded the greatest number (9) of DEGs, with models that were sensitive to DIACC3010 having a greater expression of genes involved in chemokine/cytokine and immune-related signaling and angiogenesis/cancer cell microenvironment (Fig. 8a; *CXCL10, CXCL11, IL1A, IL16, S100A12, CLEC7A, VEGFC, WNT4)*. Two DEGs were detected in models sensitive to the DIACC3010 + trastuzumab combination in the Group D comparison (Fig. 8b; *ITGB3, COL11A2*), both being involved in extracellular matrix (ECM) and invasion. No significant DEGs were detected for the Group E non-responder comparison (Fig. 8c; adjusted *p* > 0.05). Two DEGs were identified for both the Group B/trastuzumab-sensitive models (tumor-specific antigens *CTAG1B, MAGEC1*; Supplementary Fig. S8) and the Group C/all treatment-sensitive models (*HLA-DRB5, RNL5*; Supplementary Fig. S8).

## Discussion

In this study we confirmed the potential of the p70S6K/Akt dual inhibitor DIACC3010 to treat GC by employing preclinical models of GC in vitro and in vivo. Inhibiting the PAM pathway at two nodes offers a relatively novel way of attenuating oncogenic signaling in GC and provides a mechanism to block signaling from the Akt up-regulation that occurs with PAM pathway inhibition^14^. Therefore, DIACC3010 has a potential advantage in this indication compared to agents that inhibit the PAM pathway at a single node.

We examined the effects of DIACC3010 both as a monotherapy and as a combination partner with trastuzumab, which is approved for use in HER2-positive GC. Cell lines that were sensitive to DIACC3010 had *PIK3CA* mutations and low pERK. DIACC3010 monotherapy was significantly efficacious in a third of all GC PDX models tested and in 38% that were HER2-negative. We found that *ERBB2-*amplified cell lines and PDX models were not sensitive to DIACC3010 monotherapy, but most responded to trastuzumab treatment either alone or in combination with DIACC3010. We also identified models that responded synergistically to the combination, even though neither monotherapy inhibited tumor growth.

Considering the differential responses of preclinical models to DIACC3010 in the current study, as well as variable responses of patients’ tumors to PAM pathway inhibitors in past clinical trials, we attempted to identify predictive biomarkers of sensitivity or resistance to DIACC3010 and/or its combination with trastuzumab. Based on our data, we propose that relatively high pERK protein in GC tumors is a potential marker of resistance to DIACC3010 and a reduction in pERK levels, upon treatment with DIACC3010 combined with trastuzumab, is a potential response marker for the combination. These starting points will need to be confirmed by additional preclinical studies and/or in a clinical trial.

The expression of pERK indicates involvement of the MAPK pathway, which is known to contribute to multiple processes in GC^31^. One might suggest that a combination of DIACC3010 plus a MEK inhibitor could be efficacious in GC. Indeed, our results are similar to those of Liu et al. who reported an up-regulation of pERK in GC cells treated with the mTOR inhibitor everolimus and the reversal of resistance to everolimus by combining it with the MEK inhibitor trametinib^32^. Our data, however, support the potential of DIACC3010 monotherapy to treat a subset of patients with GC tumors that are HER2-negative with relatively low basal pERK.

The promise of PAM pathway inhibitors in treating GC has as yet been unrealized. This is despite the involvement of the PAM pathway in GC disease and the reported preliminary activity of inhibitors both preclinically and in clinical trials^33^, including Akt inhibitors MK-2206^34^ and ipatasertib^11^ and the mTOR inhibitor everolimus^8,9^. In these cases, the trial investigators theorized that a better response might have been seen with a subset of patients identified through tumor biomarkers. For example, analysis of biomarker data from the Phase II everolimus study revealed an association of high baseline pS6 (Ser240/4) expression with higher disease control rate and prolonged progression-free survival, thus representing a candidate predictive biomarker for everolimus^35^. However, we did not observe that pS6 was a potential biomarker in our GC studies. The recommended doses of these drug candidates are also limited by safety issues, preventing the continuous use of high drug exposure.

In more recent GC clinical trials, investigators have been incorporating biomarkers to stratify patients. In the VIKTORY umbrella GC trial^36^, treatment with pan-Akt inhibitor capivasertib + paclitaxel in patients with tumor expressing *PIK3CA* mutations resulted in 33.3% overall response rate (ORR). The choice of mutated *PIK3CA* as a potential marker for response to PAM pathway inhibitors is consistent with previous evidence from breast cancer^37^, GC^38–40^, and other tumor types^41^. In the current study, we detected *PIK3CA* mutations in three GC cell lines; the two that responded to DIACC3010 monotherapy harbored the E542K mutation. This E542K mutation was present in GC tumors from patients with the greatest response to pan-Akt inhibitor capivasertib in the VIKTORY trial^36^, whereas the more resistant cell line in our study carried the E545K mutation, which was associated with less of a response in the VIKTORY trial. We also detected the E545G *PIK3CA* mutation in one PDX model that responded to DIACC3010 monotherapy with tumor stasis. This low prevalence of mutant *PIK3CA* in the GC PDX panel in the present study (1/27, 4%) is close to the 7% seen in GC tumors in patients^28^. Based on these preclinical data, we consider E542K and E545G *PIK3CA* mutations and lack of HER2 amplification as promising biomarkers of sensitivity to DIACC3010 in GC, in addition to the relatively low basal pERK as mentioned above. Treating a larger set of preclinical HER2 negative *PIK3CA* mutated models with DIACC3010 may provide preclinical proof-of-concept for *PIK3CA* mutations as predictive biomarkers of sensitivity to this compound. In addition, the DEGs involved in chemokine/cytokine signaling for the Group A models may represent a potential multi-gene biomarker of sensitivity to DIACC3010.

Anti-HER2-based therapy is the current standard of care for HER2+ GC in the first-line setting in many regions, and primary and acquired resistance are significant challenges for patients^42^. In our study, the HER2-overexpressing GC cell lines NCI-N87 and OE‑19 were insensitive to DIACC3010 monotherapy, possibly due to DIACC3010-induced pERK elevation, whereas DIACC3010 + trastuzumab synergistically inhibited in vitro growth of OE-19 cells, colony formation, and in vivo growth of OE-19 tumors in mice. Consistent with the in vitro findings, DIACC3010 + trastuzumab reduced pS6 and pERK expression in vivo, showing effective dual PAM and MAPK pathway inhibition. In HER2+ NCI-N87 cells, synergy was not observed, and DIACC3010 + trastuzumab did not block up-regulation of pERK. In a previous report, the dual PI3K/mTOR inhibitor, BEZ235, had antitumor activity against HER2+ GC in PDX models by inhibiting HER2 signaling pathways, indicated by pAkt and pS6 inhibition^43^. In our study, synergy was not seen when the up-regulation of pERK by DIACC3010 in HER2-overexpressing cell line-derived tumors was not held in check by the combination with trastuzumab.

We also examined the combination in GC PDX models. The percentage of GC PDX that were *ERBB2*-amplified was 22% in the current study, which is similar to the 14–24%^44,45^ reported for other GC PDX models and within the range seen in patient tumors^29,46–49^. We did not test by FISH any GC PDX models rated as 0 or 1+ by HER2 IHC, which can have amplified *ERBB2* in the clinic^50–52^. Overall, we feel that the PDX models represented patients at a level that allows translational conclusions.

It is not surprising that responses among the models were varied considering the heterogeneity of GC, both within the tumors and among the tumors/patients^29,53^, but some patterns did emerge. Based on our data, we propose five types of potential responses to DIACC3010 and trastuzumab in GC. One is GC tumors that do not respond to DIACC3010, trastuzumab, or to their combination. We saw this in roughly 40% of the PDX models tested in vivo, which could be generally characterized by a lack of HER2 amplification and, based on cell line data, relatively high pERK. Two additional groups would be GC tumors that respond to either DIACC3010 or trastuzumab monotherapies, as discussed above. A fourth group would be sensitive to both DIACC3010 and trastuzumab monotherapies but do not respond in an additive manner to the combination. In these cases, HER2 signaling in these tumors may be through the “normal,” i.e. non-activated, PAM pathway^7,54^ linearly, such that blocking either HER2 upstream with trastuzumab or the PAM pathway downstream with DIACC3010 generates similar results.

The final group would be GC tumors that respond to the DIACC3010 + trastuzumab combination yet are resistant to either agent alone. This type of response was seen in three PDX models, but in two of them, the combination was not statistically different from the monotherapies as their effects on tumor growth were intermediate between those of the vehicle and the combination. The response seen in the GAX007 model, however, was similar to that of the OE-19 cell line xenograft. Both models were established from moderately differentiated adenocarcinomas^55^. DIACC3010 had no effects on tumor growth in either model. Both were also resistant to trastuzumab monotherapy despite being HER2 (3+) and *ERBB2*-amplified^56^, and the DIACC3010 + trastuzumab combination caused significant regression in both xenografts which was accompanied by a reduction in pERK in OE-19 tumors. Synergy of the combination was quantified in vitro for the OE-19 cells with Bliss and CFA analyses, and based on the similar in vivo results, we surmise that the GAX007 response to the combination was synergistic as well. Thus, these models represent potential GC patients whose HER2 (3+), trastuzumab-resistant tumors may be sensitive to treatment with the combination such that DIACC3010 resolves the resistance to trastuzumab.

Although this study included a variety of analyses, from in vitro cell line studies to in vivo PDX studies, there are several limitations in consideration of translation to clinical application. First, the cell lines used in our study were limited to thirteen, making it difficult to draw conclusions about the correlation between drug sensitivity and gene mutation or protein expression level. For example, there are only 3 GC cells lines with PIK3CA mutations in this study, so that number would need to be expanded in future studies to solidify conclusions about significance of the mutation. Secondly, although DIACC3010 is selective kinase inhibitor against p70S6K and Akt1/3, our study did not account for the differential contribution of Akt2 and Akt1/3 to drug sensitivity. It has been reported that inhibition of the PAM pathway with mTOR inhibitors induces feedback activation of different AKT isoforms in cell line specific manner in GI cancer models, suggesting the importance of considering Akt isoform differences when evaluating drug sensitivity^57,58^. Thirdly, Her2-targeted therapy in clinical practice is primarily adopted in combination with cytotoxic agents, whereas in the current study, the focus was on tumor sensitivity to targeted therapies without chemotherapy^59,60^. Lastly, tumor heterogeneity is thought to contribute to resistance to molecular-targeted agents in gastric cancer, which poses a challenge in translating preclinical models into the clinical setting^61^.

To conclude, we have confirmed the dual-inhibitory mechanism of action of DIACC3010 on the PAM pathway in GC and propose it should be further investigated in this disease. We have provided a foundation for a path forward to the clinic and suggest that next steps include a targeted approach that would include testing of DIACC3010 in additional models of HER2-negative GC in combination with standard of care chemotherapeutics and investigation of DEGs or signature, *PIK3CA* mutations, and low basal pERK as potential predictive markers of sensitivity. An additional next step would be to combine DIACC3010 with trastuzumab in models of HER2 (3+)/trastuzumab-resistant GC and confirm potential response markers such as a reduction in pERK and DEGs. Our combination findings are expected to be applicable to recently approved fam-trastuzumab-deruxtecan-nxki as well. Overall, this study shows that an “all comers” approach resulted in 59% of the PDX models responding to DIACC3010 + trastuzumab, and 38% of the models for which trastuzumab treatment was not applicable responded to DIACC3010 alone, which supports the use of DIACC3010 to treat GC in the clinic.

### Supplementary Information

Supplementary Information.

## Data Availability

All data supporting the findings of this study are included in this published article and its supplementary material file. Full datasets are available upon reasonable request to the corresponding author.
