# Supplementary material for: p70S6K/Akt dual inhibitor DIACC3010 is efficacious in preclinical models of gastric cancer alone and in combination with trastuzumab
Source: Sci Rep. 2023 Sep 25;13:16017. doi: 10.1038/s41598-023-40612-9 (PMC10520030; doi:10.1038/s41598-023-40612-9)

p70S6K/Akt dual inhibitor DIACC3010 is efficacious in preclinical models of gastric cancer alone and in combination with trastuzumab

Shota Fukuoka[1] | Yoshikatsu Koga[3] | Mayumi Yamauchi[2] | Shigehiro Koganemaru[4] | Masahiro Yasunaga[3] |Kohei Shitara[2] | Toshihiko Doi[1,4] | Takayuki Yoshino[2] | Toshio Kuronita[5] | Brian Elenbaas[6] | Pamela Wahra[6] | Hong Zhang[6] | Lindsey Crowley[6] |Molly H. Jenkins[6] | Anderson Clark[6] | Takashi Kojima[2]

[1]Division of Experimental Therapeutics, Exploratory Oncology Research & Clinical Trial Center, National Cancer Center, Kashiwa, Japan

[2]Department of Gastroenterology and Gastrointestinal Oncology, National Cancer Center Hospital East, Kashiwa, Japan

[3]Division of Developmental Therapeutics, Exploratory Oncology Research & Clinical Trial Center, National Cancer Center Hospital, Kashiwa, Japan

[4]Department of Experimental Therapeutics, National Cancer Center Hospital East, Kashiwa, Japan

[5]Merck Biopharma Co, Ltd, Tokyo, Japan; an affiliate of Merck KGaA, Darmstadt, Germany

[6]EMD Serono Research & Development Institute, Inc., Billerica, MA, USA; a business of Merck KGaA, Darmstadt, Germany

Supplementary Material

Tables S1-S4

Figures S1-S9

Supplementary Tables

Table S1. Meta data for patient-derived xenograft (PDX) models, including mutational data of pathogenic or likely pathogenic gene mutations analyzed by Illumina AmpliSeq® Cancer HotSpot Panel v2 and determined via the Catalog of Somatic Mutations in Cancer (COSMIC) and ClinVar

**Model Information**				**Donor Patient Information**		
Model ID	Passage Implanted	HER2 IHC Score	*ERBB2* FISH Results	Sex	Age	Pathological Diagnosis – Gene Mutations of PDX Model
GAX001	FP4+3	0		M	46	Moderately differentiated adenocarcinoma – *APC R1450*, KRAS G13D*
GAX007	FP3+3	3+	amplified	M	54	Moderately differentiated adenocarcinoma
GAX018	FP2+3	2+	non amplified	F	28	Signet-ring cell carcinoma – *KRAS G12D, MLH1 V384D*
GAX023	FP3+3	1+		F	73	Poorly to moderately differentiated adenocarcinoma - *TP53 P151R*
GAX027	FP2+3	1+		M	65	Moderately differentiated adenocarcinoma – *APC E1464Vfs*8*
GAX031	FP1+3	2+	non amplified	M	70	Moderately differentiated adenocarcinoma – *TP53 Y163C*
GAX034	FP2+3	1+		(na)	(na)	Moderately differentiated adenocarcinoma - *TP53 R213**
GAX036	FP2+3	3+	amplified	M	52	Moderately differentiated adenocarcinoma - *TP53 H179R*
GAX059	FP9+5	0		M	68	Moderately differentiated adenocarcinoma – *APC R876*, TP53 R213**
GAX066	FP6+3	0		M	72	Moderately differentiated adenocarcinoma – *KRAS G12C*
GAX070	FP3+3	0		M	75	Poorly-moderately differentiated adenocarcinoma – *CTNNB1 S37C, FBXW7 R505C, TP53 R175H*
GAX150	FP5+3	0		(na)	(na)	Poorly differentiated adenocarcinoma – *MLH1 V384D, TP53 Y163C*
GAX159	FP4+3	1+		M	67	Poorly differentiated adenocarcinoma
GAX165	FP4+3	0				Moderately differentiated adenocarcinoma
GAX205	FP3+3	1+		M	63	Poorly differentiated adenocarcinoma
GAX212	FP2+3	2+	amplified	M	63	Poorly-moderately differentiated adenocarcinoma - *TP53 H193R*
GAX214	FP2+3	1+		F	71	Moderately differentiated adenocarcinoma – *GNAS R844H*
GAX219	FP1+3	2+	non amplified	(na)	(na)	Poorly-moderately differentiated adenocarcinoma – *TP53 A59T*
GAX224	FP3+3	3+	amplified	(na)	(na)	Poorly-moderately differentiated adenocarcinoma - *TP53 H214R*
GAX232	FP3+3	2+	amplified	M	46	Moderately differentiated adenocarcinoma – *SMAD4 R361H*
GAX239	FP2+3	1+		M	70	Poorly differentiated adenocarcinoma - *TP53 H179R*
GAX240	FP2+3	0		M	61	Moderately differentiated adenocarcinoma – *KRAS Q61H, TP53 M246K*
GAX256	FP3+3	0		(na)	(na)	Poorly differentiated adenocarcinoma
GAX257	FP1+3	2+	amplified	F	62	Poorly differentiated adenocarcinoma - *TP53*[‡] *P250L*

GAX267	FP3+3	2+	non amplified	(na)	(na)	Poorly-moderately differentiated adenocarcinoma – *TP53 R306**
GAX271	FP2+4	2+	non amplified	(na)	(na)	Poorly-moderately differentiated adenocarcinoma – *KRAS G13D, TP53 R248Q*
GAX272	FP1+3	2+	non amplified	(na)	(na)	Poorly-moderately differentiated adenocarcinoma – *GNAS R844C, PIK3CA E545G*

FPx+y: Frozen passage number "x" implanted into mice and then passaged "y" more times to generate enough tumor-bearing mice to enroll in the efficacy study.

(na): Information not available

‡ Mutation listed as having an uncertain pathogenic significance

Table S2. Mean percent body weight, based on Day 0 starting body weight, for each treatment group on the final day of study for each model.

Model ID	Treatment	Days of Treatment	% Body Weight	Model ID	Treatment	Days of Treatment	% Body Weight
GAX001	Vehicle	28	-1	GAX205	Vehicle	32	10
	DIACC3010 20 mg/kg		-1		DIACC3010 20 mg/kg		-3
	Trastuzumab 15 mg/kg		-3		Trastuzumab 15 mg/kg		-2
	DIACC3010 + Trastuzumab		5		DIACC3010 + Trastuzumab		-1
GAX007	Vehicle	56	12	GAX212	Vehicle	46	-15
	DIACC3010 20 mg/kg		0		DIACC3010 20 mg/kg		-13
	Trastuzumab 15 mg/kg		21		Trastuzumab 15 mg/kg		9
	DIACC3010 + Trastuzumab		-3		DIACC3010 + Trastuzumab		1
GAX018	Vehicle	39	0	GAX214	Vehicle	28	4
	DIACC3010 20 mg/kg		-9		DIACC3010 20 mg/kg		2
	Trastuzumab 15 mg/kg		4		Trastuzumab 15 mg/kg		4
	DIACC3010 + Trastuzumab		-10		DIACC3010 + Trastuzumab		10
GAX023	Vehicle	32	9	GAX219	Vehicle	28	5
	DIACC3010 20 mg/kg		2		DIACC3010 20 mg/kg		-4
	Trastuzumab 15 mg/kg		10		Trastuzumab 15 mg/kg		4
	DIACC3010 + Trastuzumab		-6		DIACC3010 + Trastuzumab		4
GAX027	Vehicle	32	3	GAX224	Vehicle	18	0
	DIACC3010 20 mg/kg		-1		DIACC3010 20 mg/kg		-19
	Trastuzumab 15 mg/kg		-3		Trastuzumab 15 mg/kg		2
	DIACC3010 + Trastuzumab		1		DIACC3010 + Trastuzumab		-10
GAX031	Vehicle	28	15	GAX232	Vehicle	35	14
	DIACC3010 20 mg/kg		5		DIACC3010 20 mg/kg		2
	Trastuzumab 15 mg/kg		13		Trastuzumab 15 mg/kg		13
	DIACC3010 + Trastuzumab		6		DIACC3010 + Trastuzumab		7
GAX034	Vehicle	66	13	GAX239	Vehicle	56	6
	DIACC3010 20 mg/kg		2		DIACC3010 20 mg/kg		5
	Trastuzumab 15 mg/kg		2		Trastuzumab 15 mg/kg		11
	DIACC3010 + Trastuzumab		-3		DIACC3010 + Trastuzumab		5
GAX036	Vehicle	17	8	GAX240	Vehicle	25	8
	DIACC3010 20 mg/kg		-4		DIACC3010 20 mg/kg		-12
	Trastuzumab 15 mg/kg		8		Trastuzumab 15 mg/kg		3
	DIACC3010 + Trastuzumab		-3		DIACC3010 + Trastuzumab		-4
GAX059	Vehicle	21	20	GAX256	Vehicle	28	4
	DIACC3010 20 mg/kg		4		DIACC3010 20 mg/kg		-5
	Trastuzumab 15 mg/kg		13		Trastuzumab 15 mg/kg		-9
	DIACC3010 + Trastuzumab		-6		DIACC3010 + Trastuzumab		-5
GAX066	Vehicle	21	4	GAX257	Vehicle	28	14
	DIACC3010 20 mg/kg		-7		DIACC3010 20 mg/kg		0
	Trastuzumab 15 mg/kg		7		Trastuzumab 15 mg/kg		9
	DIACC3010 + Trastuzumab		-9		DIACC3010 + Trastuzumab		3
GAX070	Vehicle	39	5	GAX267	Vehicle	28	8
	DIACC3010 20 mg/kg		-11		DIACC3010 20 mg/kg		-2
	Trastuzumab 15 mg/kg		2		Trastuzumab 15 mg/kg		14
	DIACC3010 + Trastuzumab		-11		DIACC3010 + Trastuzumab		1

GAX150	Vehicle	18	9	GAX271	Vehicle	21	0
	DIACC3010 20 mg/kg		0		DIACC3010 20 mg/kg		-5
	Trastuzumab 15 mg/kg		7		Trastuzumab 15 mg/kg		2
	DIACC3010 + Trastuzumab		-2		DIACC3010 + Trastuzumab		0
GAX159	Vehicle	21	9	GAX272	Vehicle	28	5
	DIACC3010 20 mg/kg		6		DIACC3010 20 mg/kg		-5
	Trastuzumab 15 mg/kg		9		Trastuzumab 15 mg/kg		1
	DIACC3010 + Trastuzumab		8		DIACC3010 + Trastuzumab		-5
GAX165	Vehicle	17	0				
	DIACC3010 20 mg/kg		-9				
	Trastuzumab 15 mg/kg		3				
	DIACC3010 + Trastuzumab						

Table S3. **TruSeq Amplicon Cancer Panel Gene List**

ABL1	*EGFR*	*GNAS*	*MLH1*	*RET*
AKT1	*ERBB2*	*HNF1A*	*MPL*	*SMAD4*
ALK	*ERBB4*	*HRAS*	*NOTCH1*	*SMARCB1*
APC	*FBXW7*	*IDH1*	*NPM1*	*SMO*
ATM	*FGFR1*	*JAK2*	*NRAS*	*SRC*
BRAF	*FGFR2*	*JAK3*	*PDGFRA*	*STK11*
CDH1	*FGFR3*	*KDR*	*PIK3CA*	*TP53*
CDKN2A	*FLT3*	*KIT*	*PTEN*	*VHL*
CSF1R	*GNA11*	*KRAS*	*PTPN11*	
CTNNB1	*GNAQ*	*MET*	*RB1*	

Table S4. AmpliSeq for Illumina Cancer HotSpot Panel Gene List

ABL1	*EGFR*	*GNAQ*	*KRAS*	*PTPN11*
AKT1	*ERBB2*	*GNAS*	*MET*	*RB1*
ALK	*ERBB4*	*HNF1A*	*MLH1*	*RET*
APC	*EZH2*	*HRAS*	*MPL*	*SMAD4*
ATM	*FBXW7*	*IDH1*	*NOTCH1*	*SMARCB1*
BRAF	*FGFR1*	*IDH2*	*NPM1*	*SMO*
CDH1	*FGFR2*	*JAK2*	*NRAS*	*SRC*
CDKN2A	*FGFR3*	*JAK3*	*PDGFRA*	*STK11*
CSF1R	*FLT3*	*KDR*	*PIK3CA*	*TP53*
CTNNB1	*GNA11*	*KIT*	*PTEN*	*VHL*

Supplementary Figures

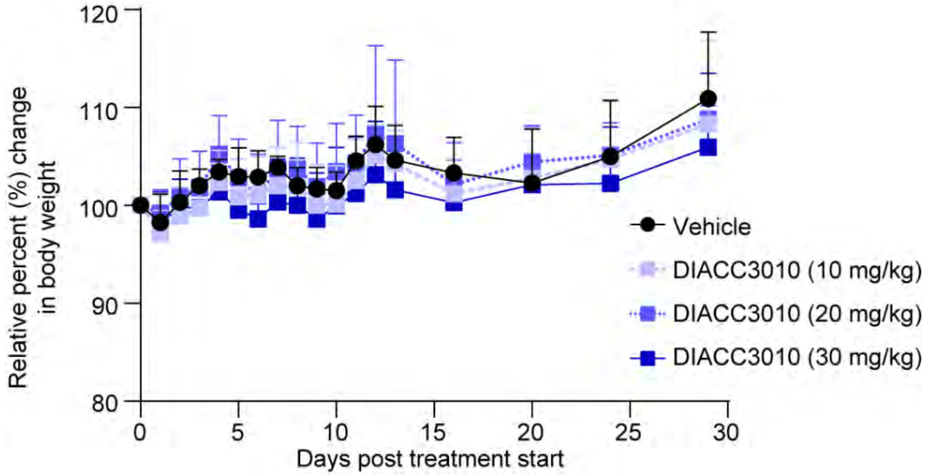

FIGURE S1. DIACC3010 did not affect body weight in the HGC-27 xenograft model. Mice were injected subcutaneously in the flank with 1 x 10^7 HGC-27 cells. Once tumors reached approximately 200 mm^3, mice were treated orally (PO) with vehicle or DIACC3010 at 10, 20, or 30 mg/kg daily (QD; n = 7) starting Day 0 for 14 days. Body weights (BW) were recorded at the time of treatment and for an additional 16 days after treatment. %BW relative to starting BW is shown (mean ± SD) and was unaffected by DIACC3010 treatment throughout the study.

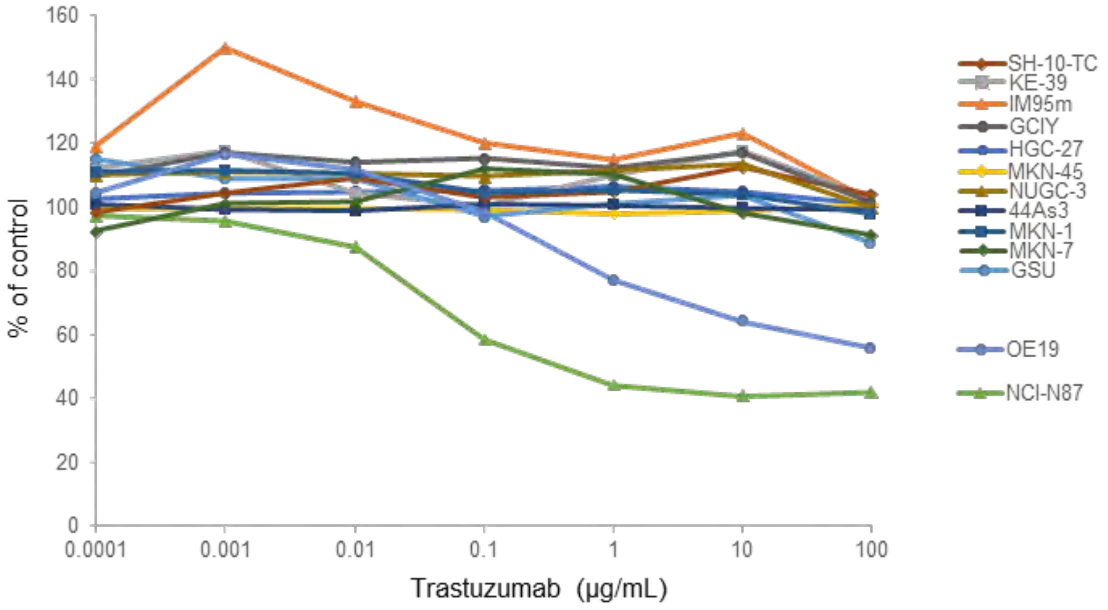

FIGURE S2. Sensitivity to trastuzumab of 13 gastric cancer (GC) cell lines. The growth-inhibitory effects of trastuzumab were measured using a tetrazolium salt-based WST-8 proliferation assay. Cells were seeded into six 96-well plates and treated for 72 hours (h) with DIACC3010 (0–10 µM; studies repeated 2-5 times). The sensitive cell lines OE-19 (GI_{50} 112 µg/mL) and NCI-N87 (GI_{50} 0.8 µg/mL) were also the 2 lines that expressed relatively high levels of HER2 protein.

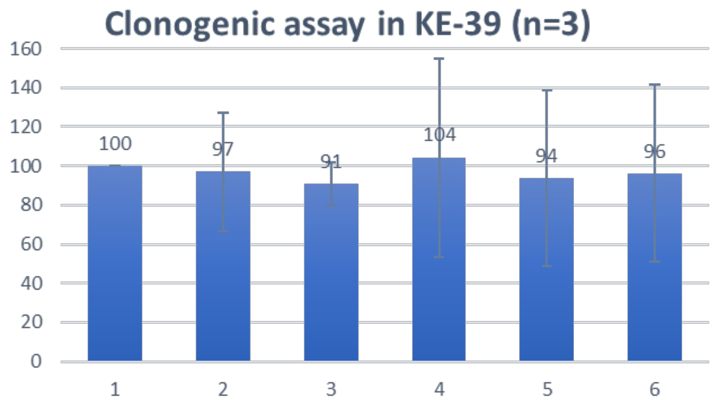

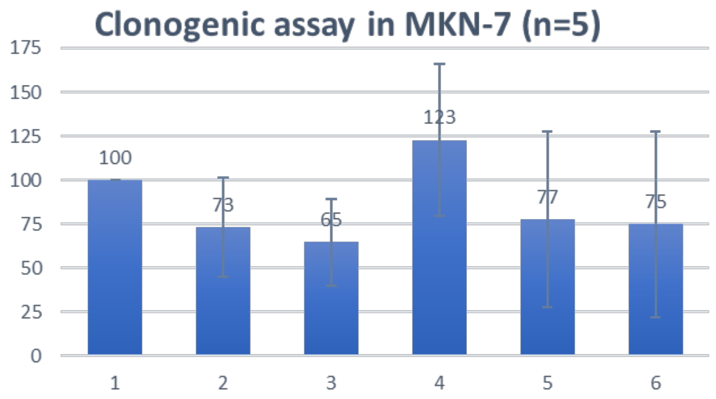

FIGURE S3. Synergy of DIACC3010 + trastuzumab was not detected in the colony formation assay of KE-39 or MKN-7 cells (mean % of control ± SD).

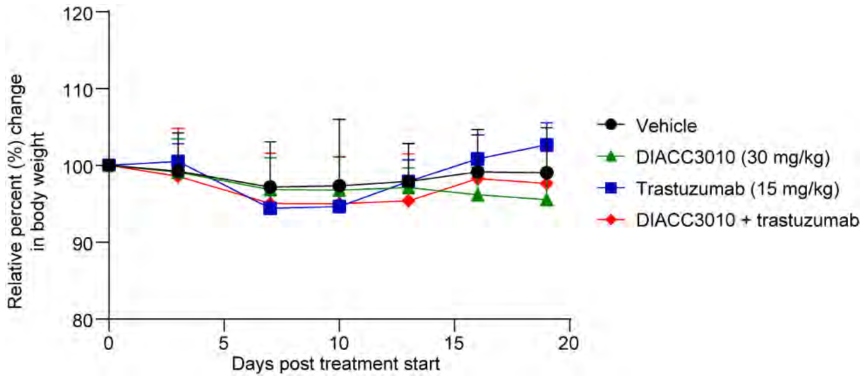

FIGURE S4. DIACC3010 did not affect body weight in the OE-19 xenograft model. Mice were injected subcutaneously in the flank with 1 x 10^7 OE-19 cells. Once tumors reached approximately 200 mm^3, mice were assigned to treatments (n = 6): vehicle, DIACC3010 at 30 mg/kg orally/daily (po, QD), trastuzumab 15 mg/kg once per week (iv, QW). Body weights (BW) were recorded at the time of treatment. %BW (mean ± SD) relative to starting BW is shown below and appears to have been unaffected by any treatment throughout the study.

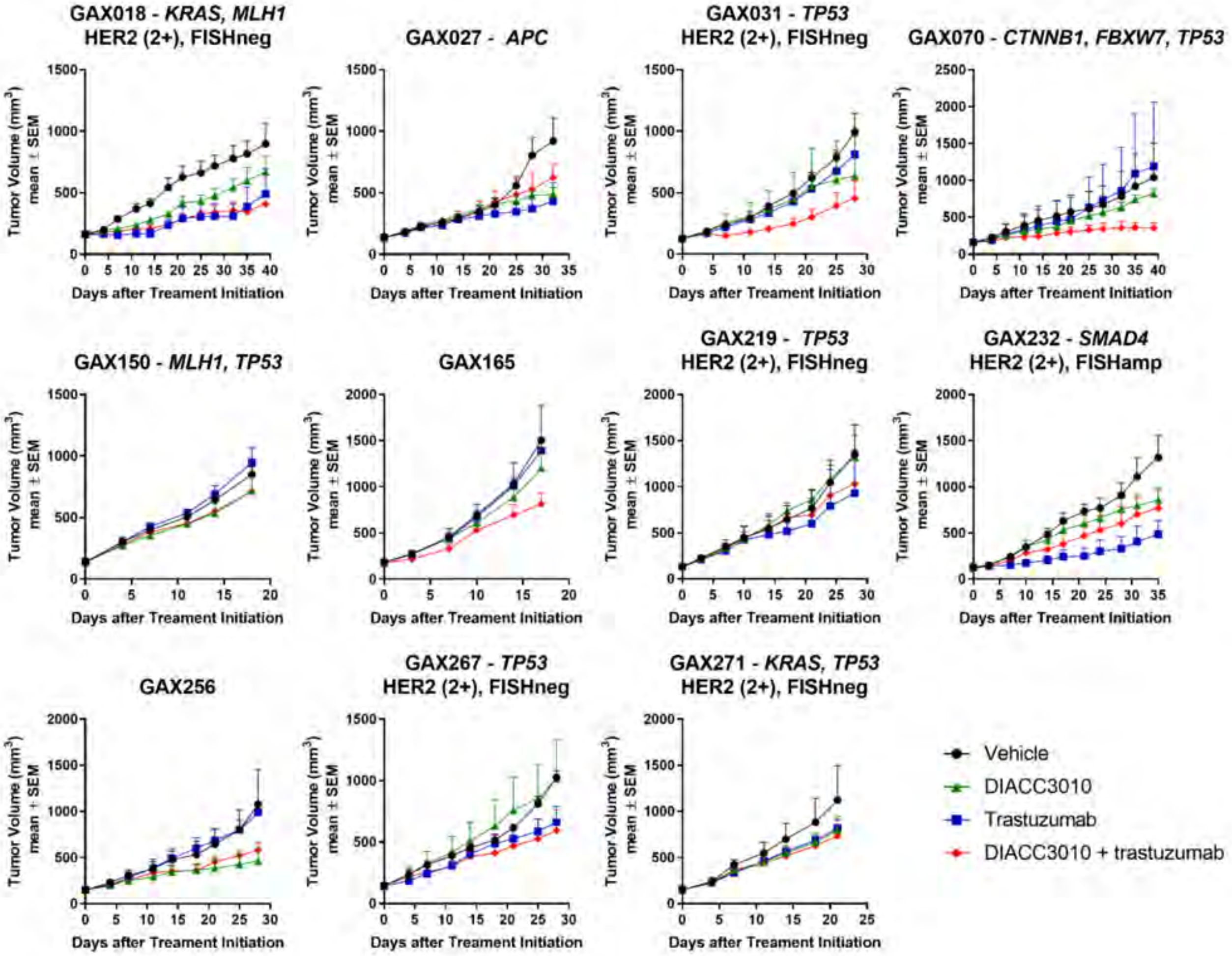

FIGURE S5. DIACC3010 and trastuzumab, either alone or in combination, had no significant effects on tumor growth in 11 patient-derived xenograft models of GC. The model name, mutated oncogenic hotspot genes, and HER2 status are given above each graph. A lack of given HER2 status means the model was HER2 (1+) or (0).

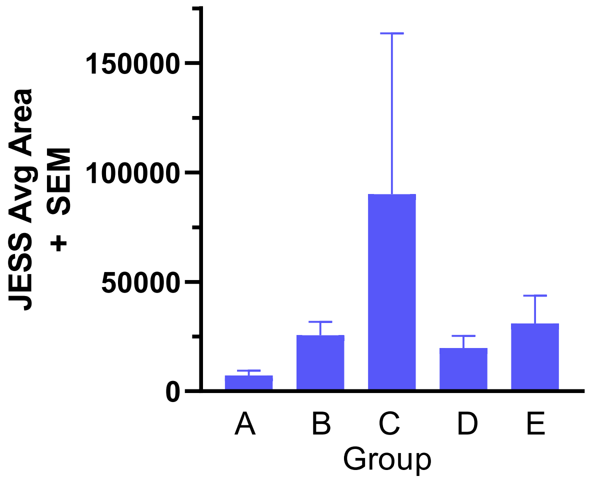

Figure S6. Immunomodulatory analysis was performed following the ProteinSimple (South Wallingford, CT) Jess 2 Immunoassays RePlex Module protocol provided with the EZ standard pack 1 of 12-230 kDa kit. Tumor lysates were probed with phosphorylated (p)ERK1/2 antibody (Cell Signaling Technology, catalog #4370) and total (t)ERK1/2 antibody (Cell Signaling Technology, catalog #4695), according to manufacturer's protocol at a dilution of 1:1000 per well. Data analysis was performed using the Compass for SW software version 5.0 where the high dynamic range detection profile exposure; area results for pERK were divided by area results for tERK to yield a corrected area. Models were grouped according to treatment response; those that responded to DIACC3010 either alone or in combination with trastuzumab (A), models that responded to trastuzumab either alone or in combination with DIACC3010 (B), models that responded to DIACC3010 and trastuzumab either alone or in combination (C), models that only responded to the combination of DIACC3010 and trastuzumab (D), and models that did not respond to any of the treatments (E). Corrected areas of Group A were compared to the corrected areas of groups B-E by unpaired t-test with Welch's two-tailed correction using GraphPad Prism version 9.1.2 ($p = 0.09$).

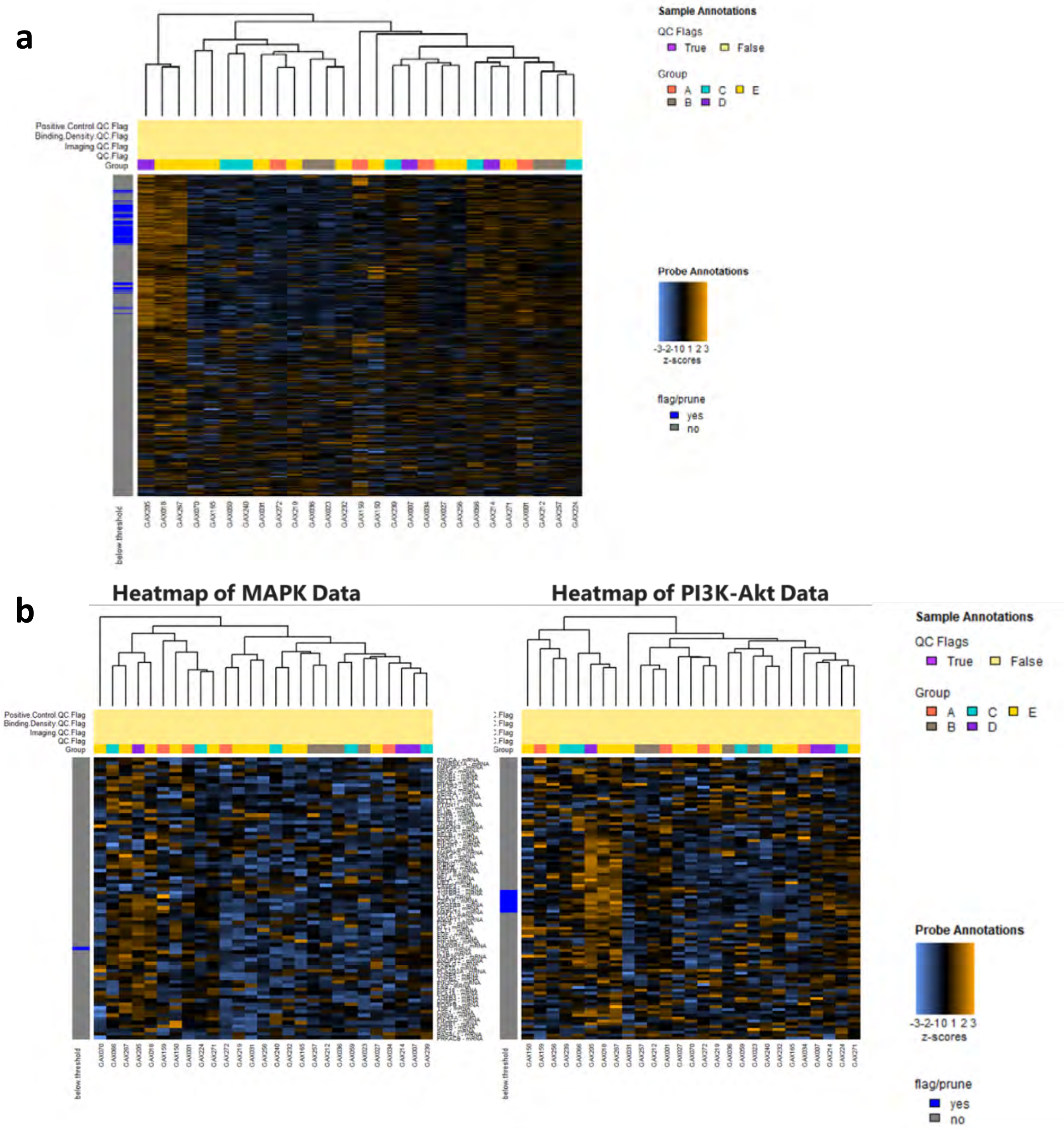

Figure S7. Heatmaps of clustering analyses of (**a**) all genes and (**b**) those assigned to the MAPK and PI3K-Akt pathways from the nanoString analysis. For all heatmaps, unsupervised clustering was generated from normalized data that was scaled to give all genes equal variance. Orange indicates relatively high expression and blue indicates relatively low expression. (**a**) Overall, some clustering in gene expression is seen but is not related to group. (**b**) Some clustering is seen for MAPK genes, especially with Group B and D (Figure 8), which appear to be differentially expressed compared to Group E (Figure S5). (**b**) The 2 major clades for the PI3K-Akt genes are not associated with groups.

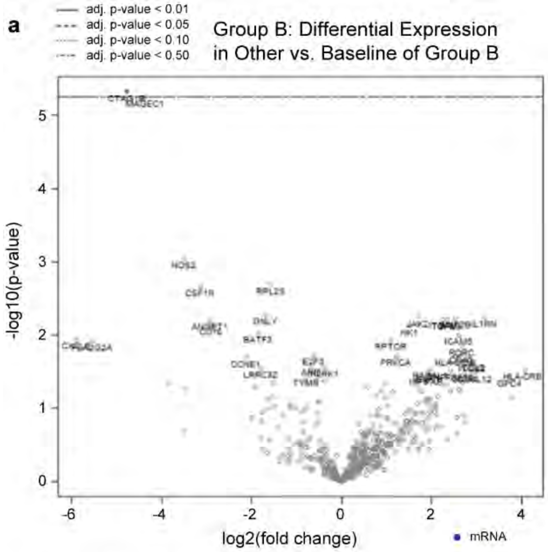

Differential Expression in Group B Baseline vs. All Other Samples

Gene	Log2 fold change vs. all Others	Standard Error (log2)	BY adjusted p-value	nanoString Gene Sets	Probe ID
CTAG1B	4.77	0.823	0.013	(not assigned)	NM_001327.2:285
MAGEC1	4.36	0.761	0.013	(not assigned)	NM_005462.4:338

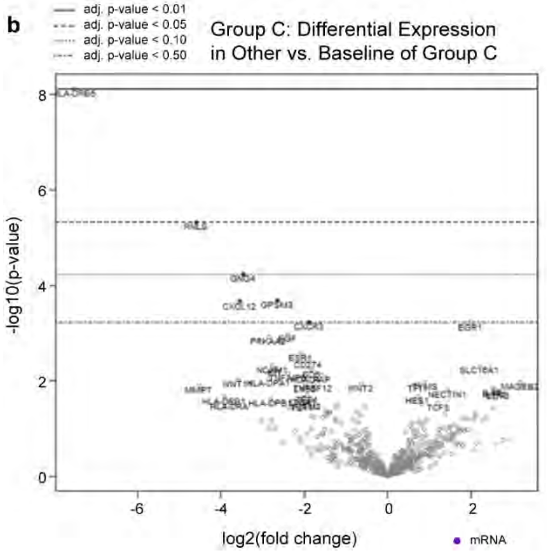

Differential Expression in Group C Baseline vs. All Other Samples

Gene	Log2 fold change vs. all Others	Standard Error (log2)	BY adjusted p-value	nanoString Gene Sets	Probe ID
HLA-DRB5-mRNA	7.53	0.886	0.001	Antigen Presentation, Costimulatory Signaling, Immune Cell Adhesion and Migration, Interferon Signaling	NM_002125.3:135
RNLS-mRNA	4.58	0.789	0.011	(not assigned)	NM_001031709.2:726

Figure S8. Volcano plots of relative expression of genes in the nanoString PanCancer IO 360 panel in gastric cancer patient-derived xenograft tumors. X-axes show lower (to the left of 0) or higher (to the right of 0) log2-fold changes in expression of genes in all other tumors versus those in Group B (trastuzumab-sensitive; Panel **a**) or Group C (equally sensitive to both monotherapies and combination; Panel **b**). Y-axes show the -log10 unadjusted p-value, and horizontal lines denote levels of Benjamini-Yekutieli (BY) corrected adjusted p-values. Tables to the right of each plot list those genes differentially expressed by at least 2.0 log2-fold and statistically significant with an adjusted p-value of < 0.05. 2 genes were significantly expressed by both Group B models (**a**) and Group C models (**b**), respectively, at more than 2-(log2)-fold versus all other models.

Figure S9. The following pages contain the images of the full, uncropped western blots. For each blot, the figure number of the corresponding cropped blot is given. Some lanes on some of the blots are not labeled because they show data that are not a part of the studies in this paper.

Original image
Figure 2

HER2

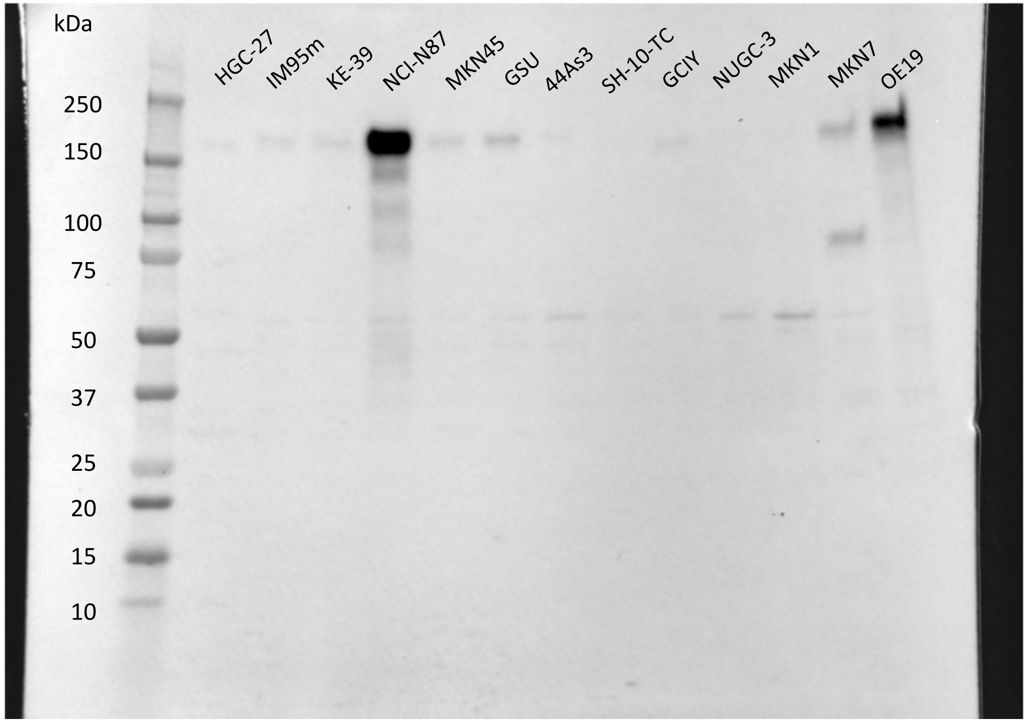

Original image
Figure 2

Phospho-HER2(Tyr1221/1222)

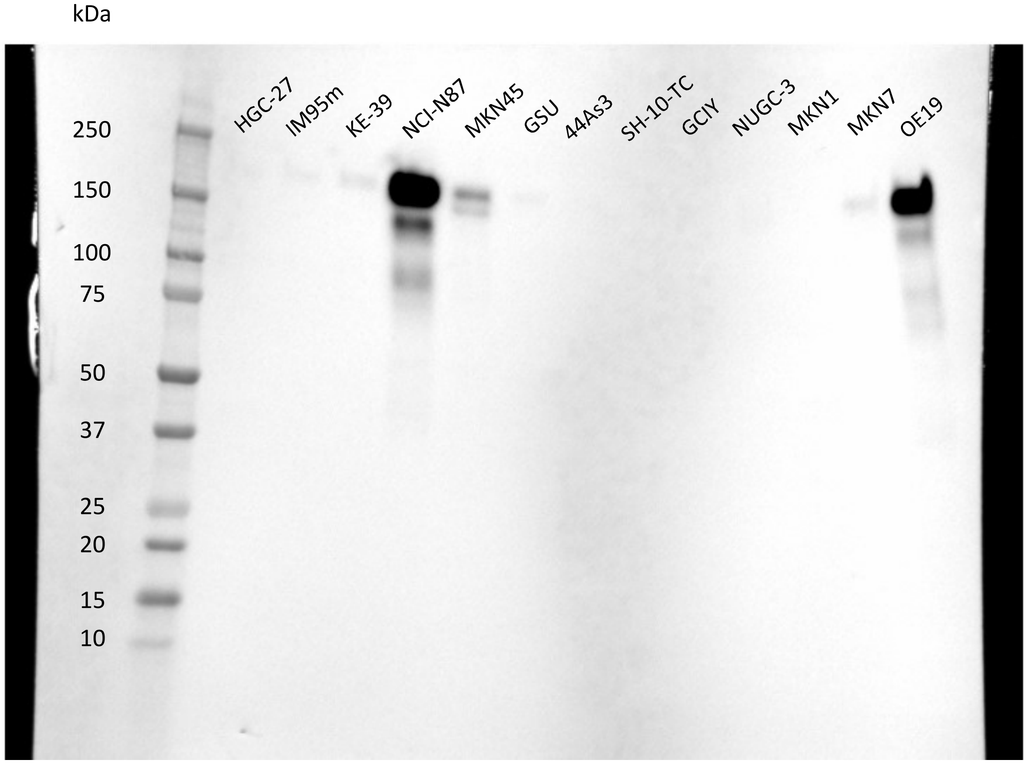

Original image
Figure 2

P44/42 MAPK (Erk1/2)

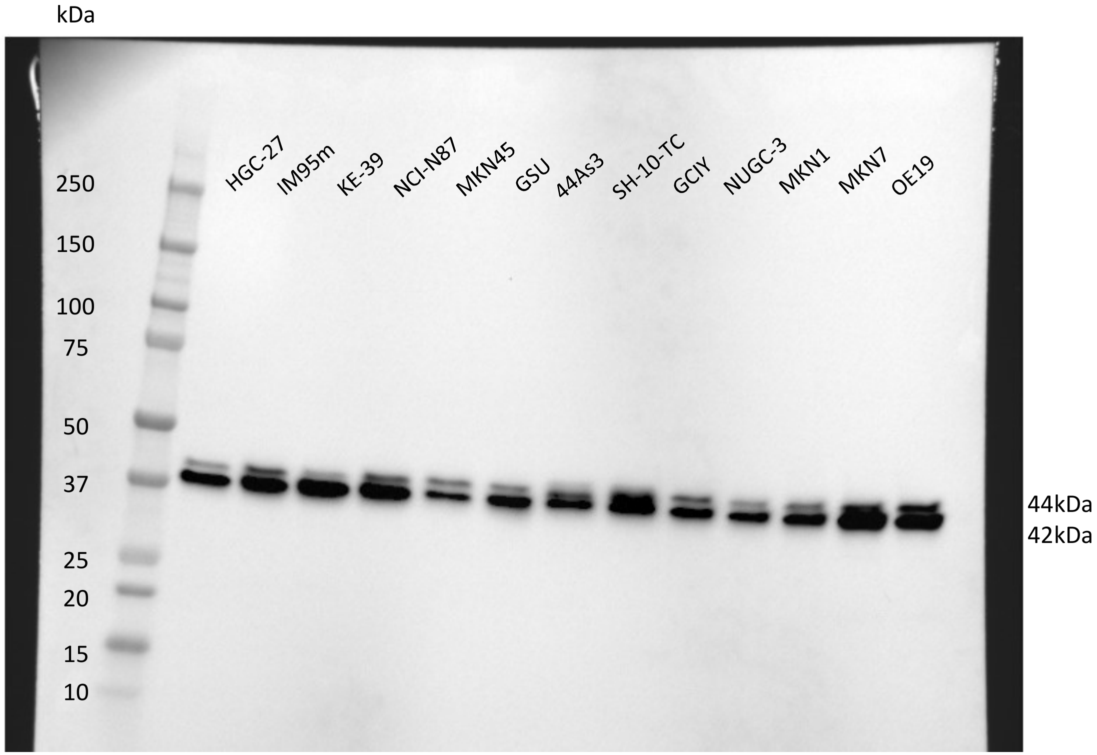

Original image
Figure 2

Phospho-p44/42 MAPK (Erk1/2)(Thr202/Tyr204)

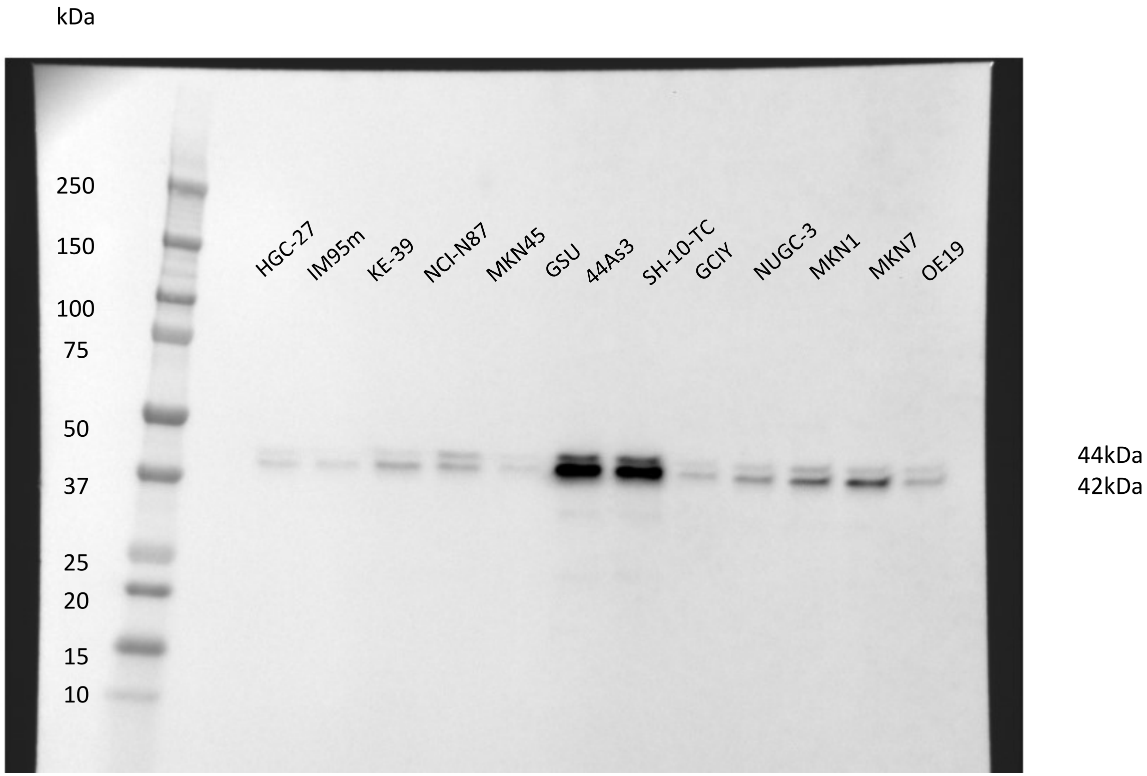

Original image
Figure 2

PTEN

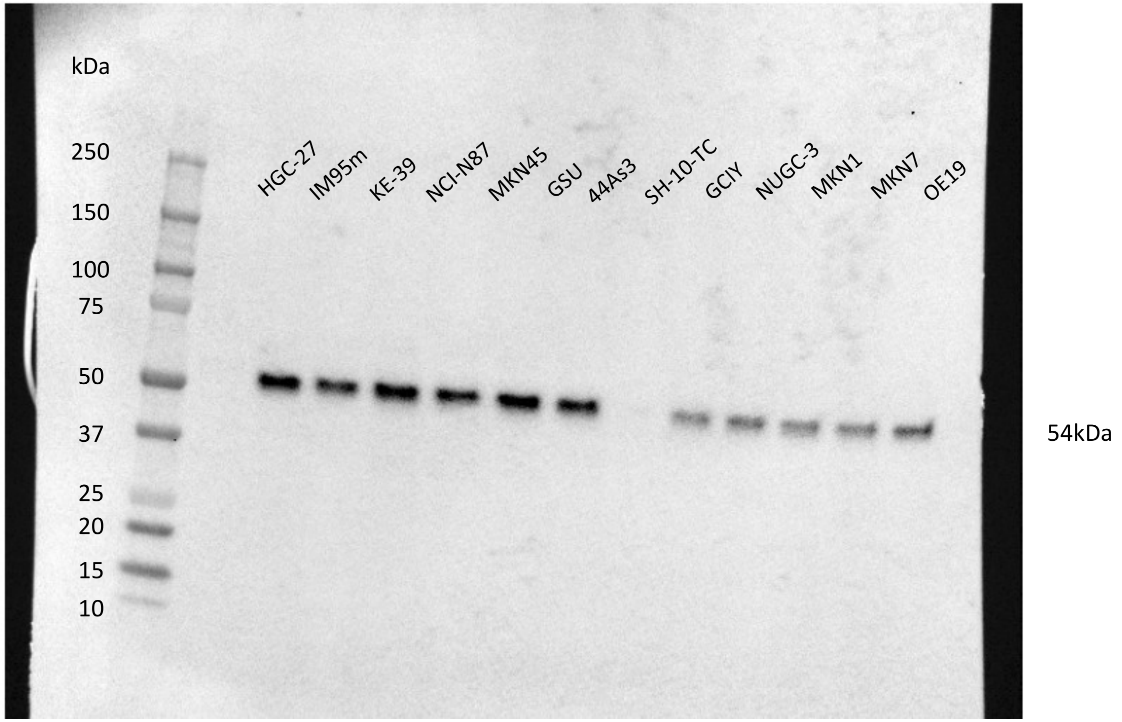

Original image
Figure 2

Akt (pan)

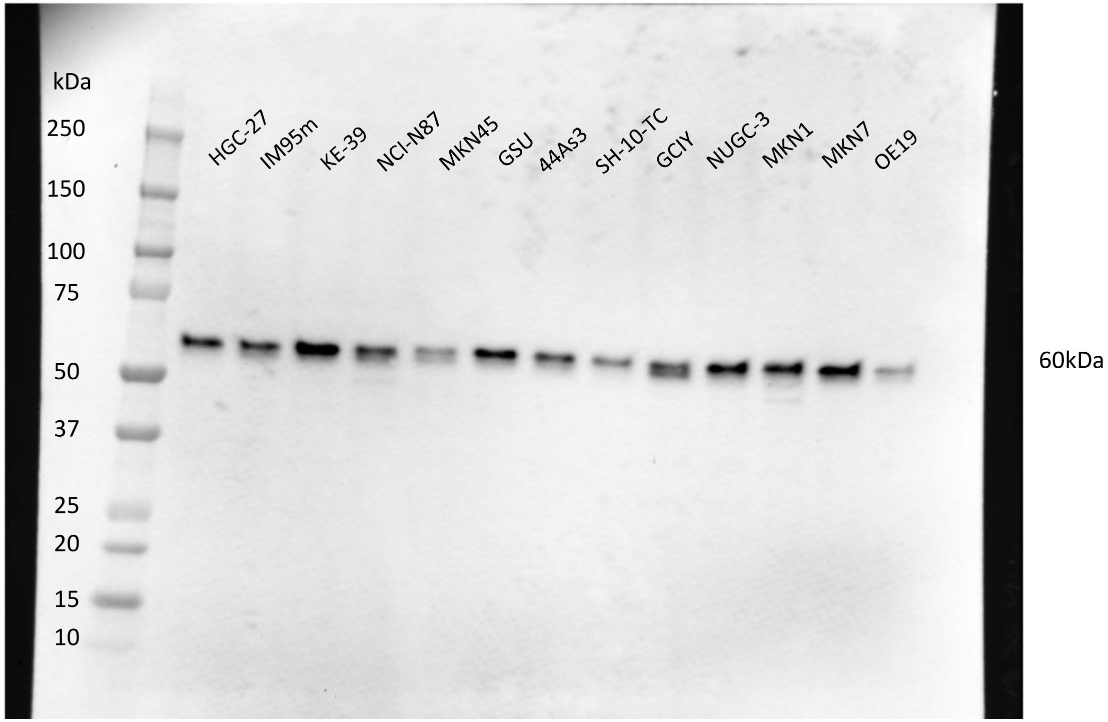

Original image
Figure 2

Phospho-Akt (Ser473)

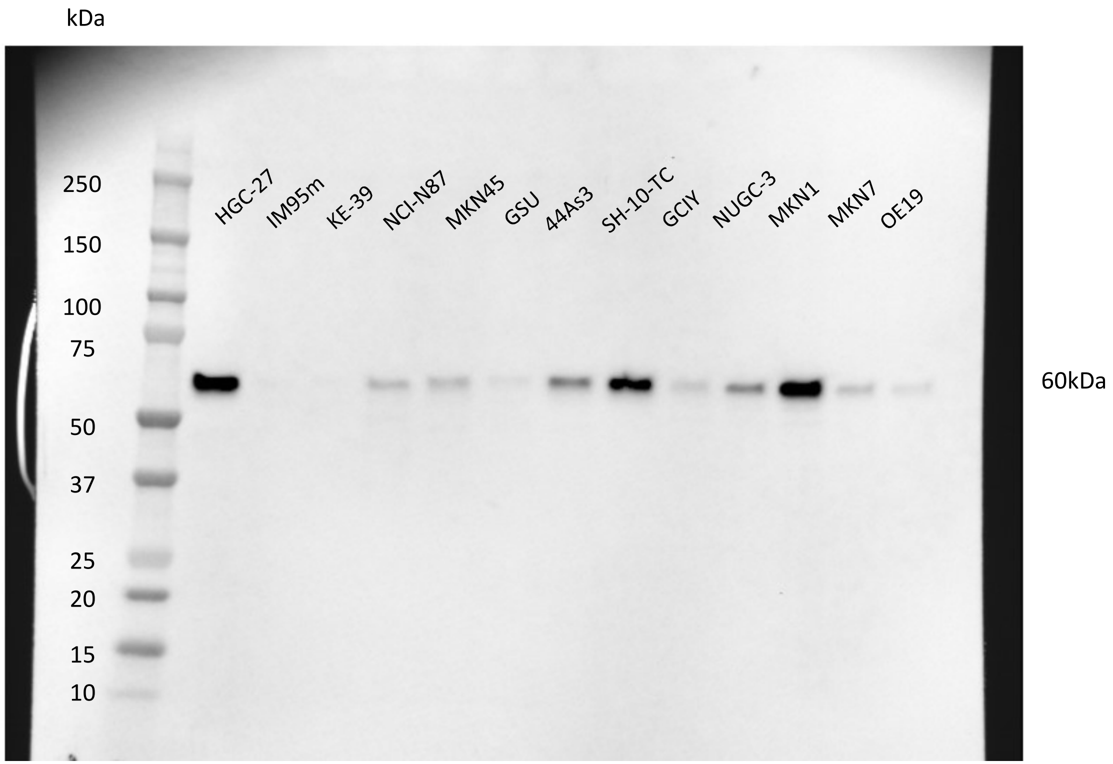

Original image
Figure 2

PRAS40

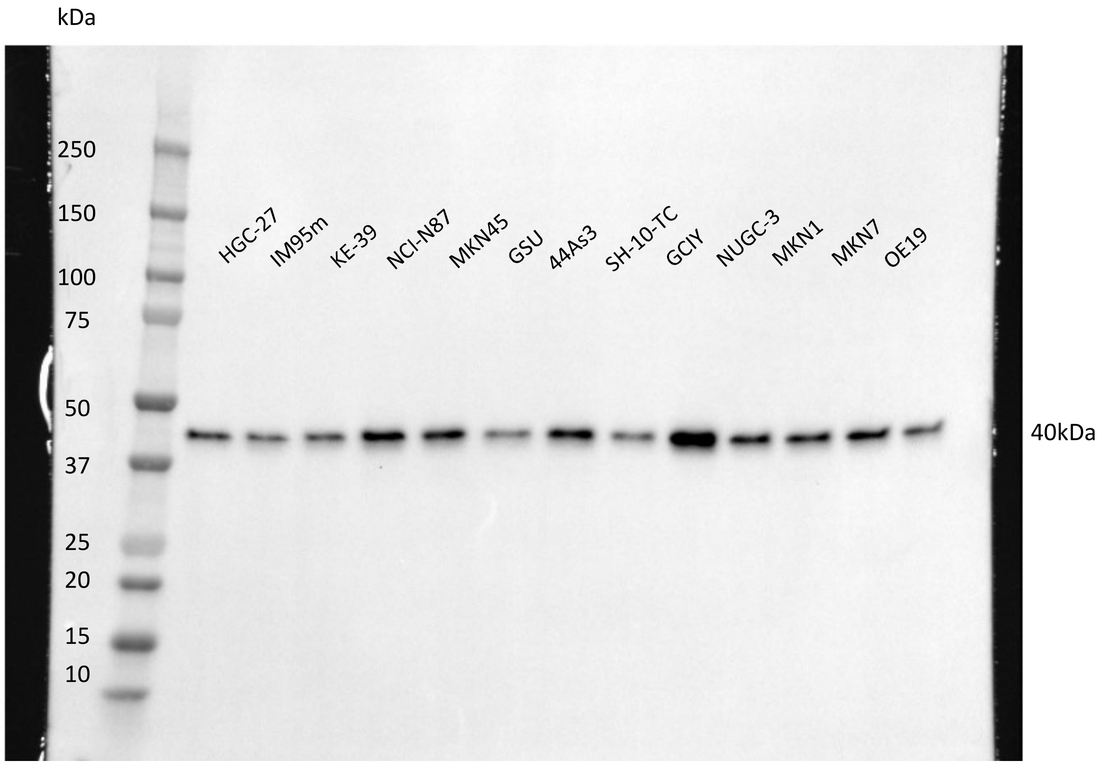

Original image
Figure 2

Phospho-PRAS40 (Thr246)

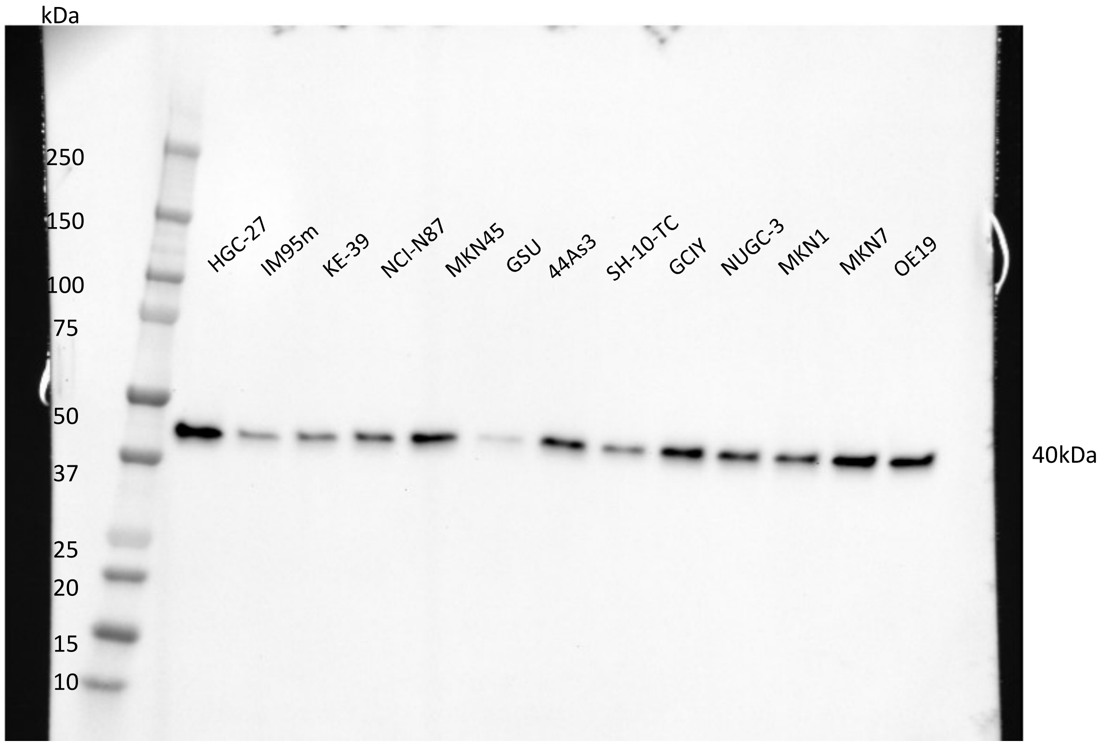

Original image
Figure 2

P70 S6 Kinase

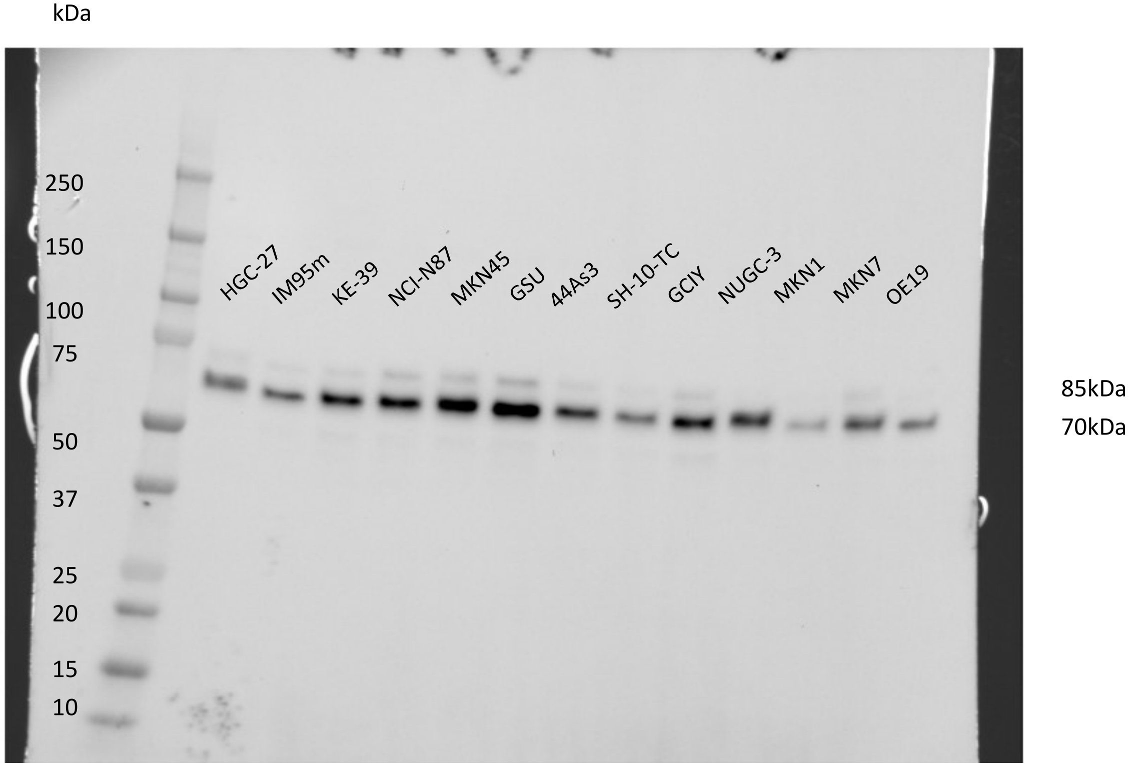

Original image
Figure 2

Phospho-P70 S6 Kinase (Thr389)

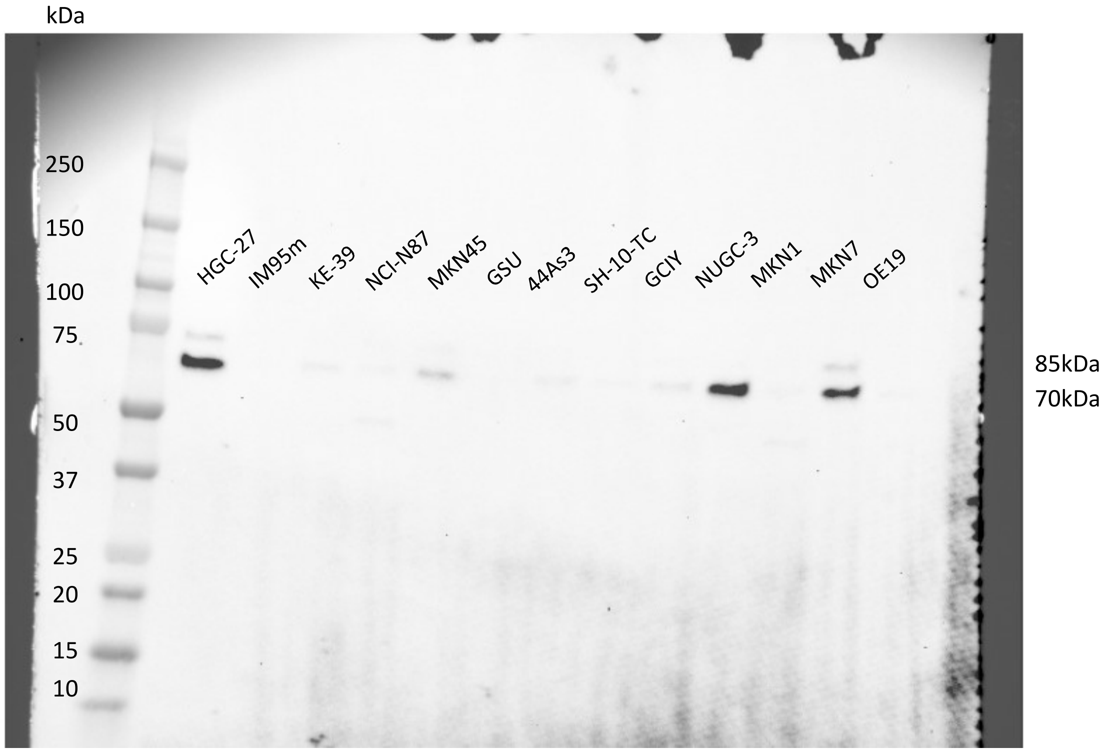

Original image
Figure 2

S6 Ribosomal protein

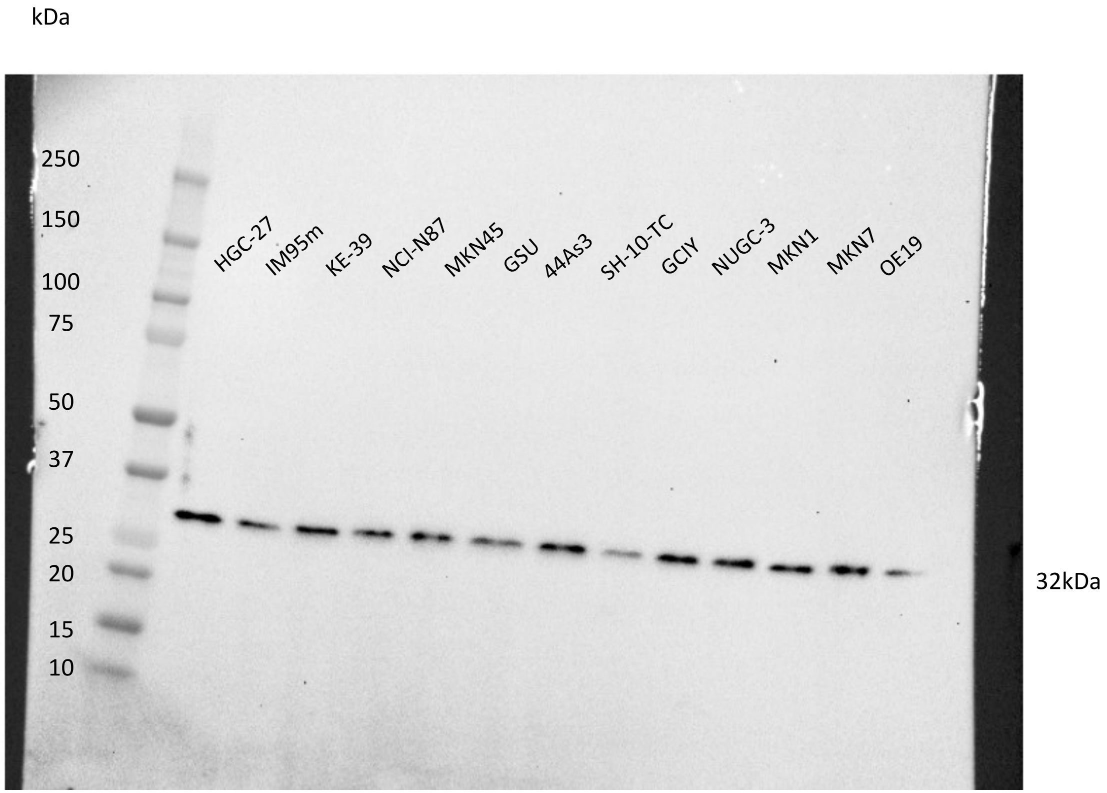

Original image
Figure 2

Phospho-S6 Ribosomal protein (Ser240/244)

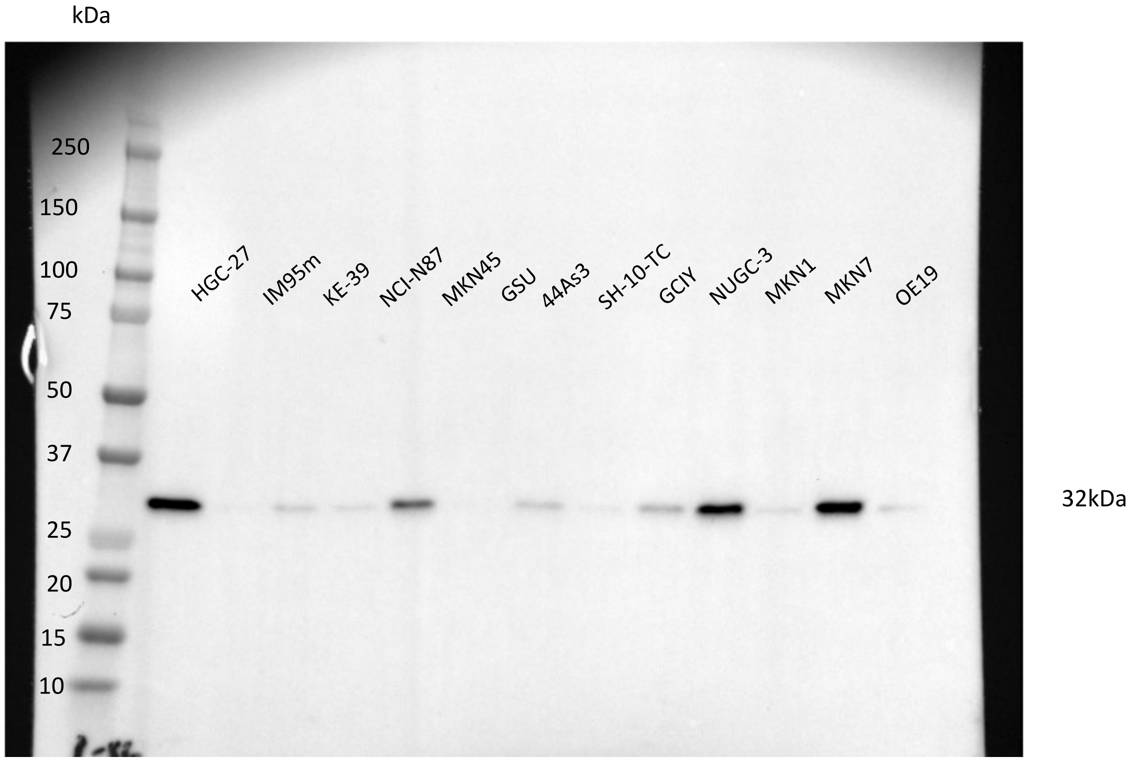

Original image
Figure 2

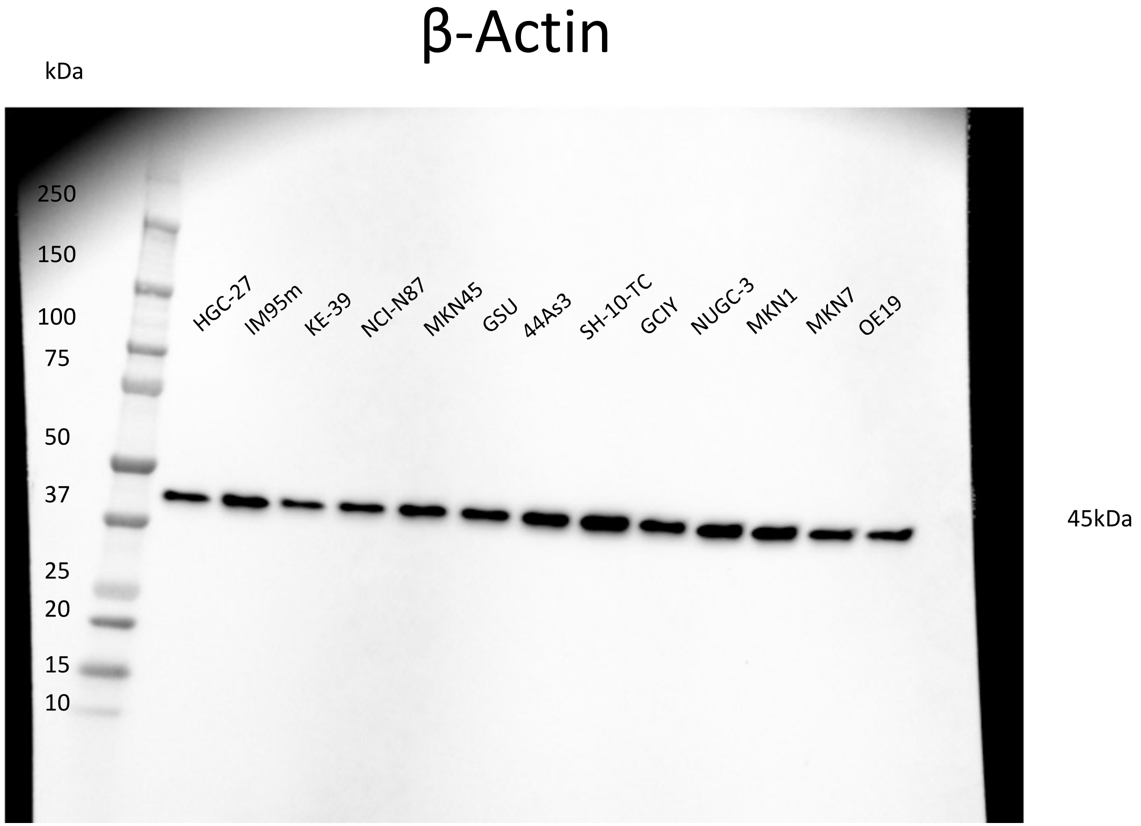

Original image
Figure 3
HGC-27

Phospho-p44/42 MAPK (Erk1/2) (Thr202/Tyr204)

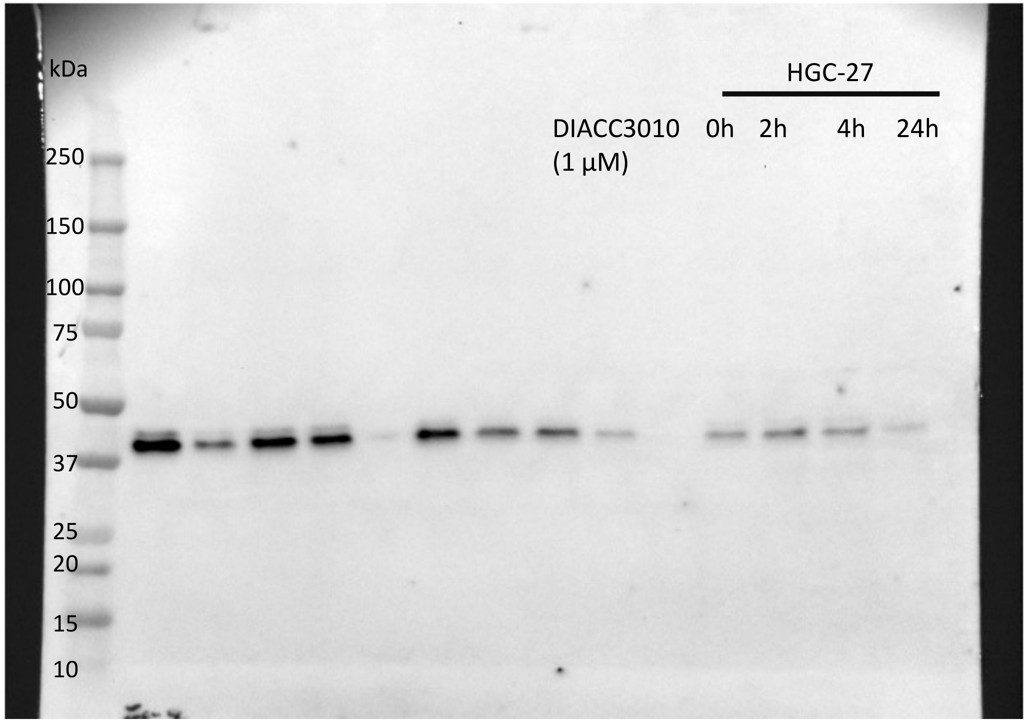

Original image
Figure 3
HGC-27

P44/42 MAPK (Erk1/2)

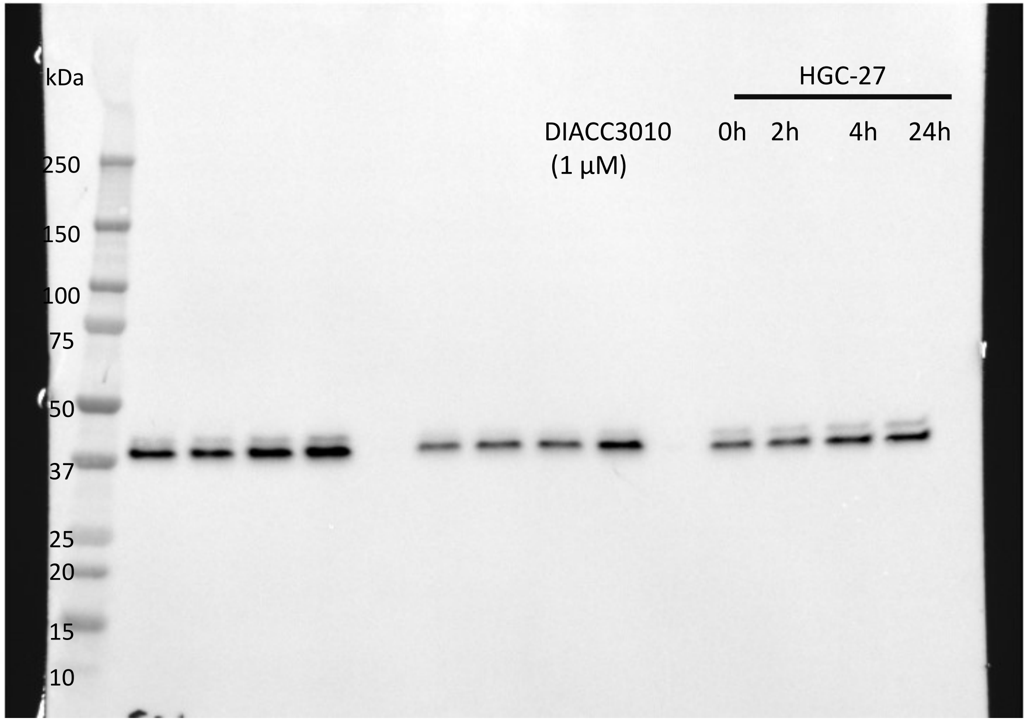

Original image
Figure 3
HGC-27

Phospho-Akt (Ser473)

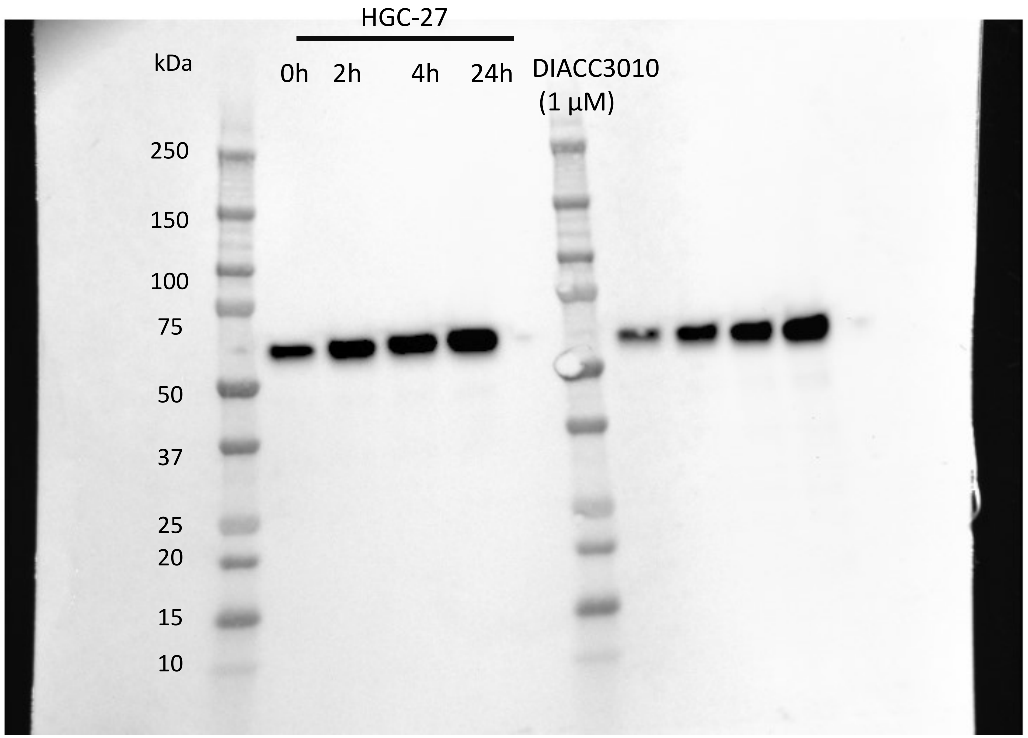

Original image
Figure 3
HGC-27

Akt (pan)

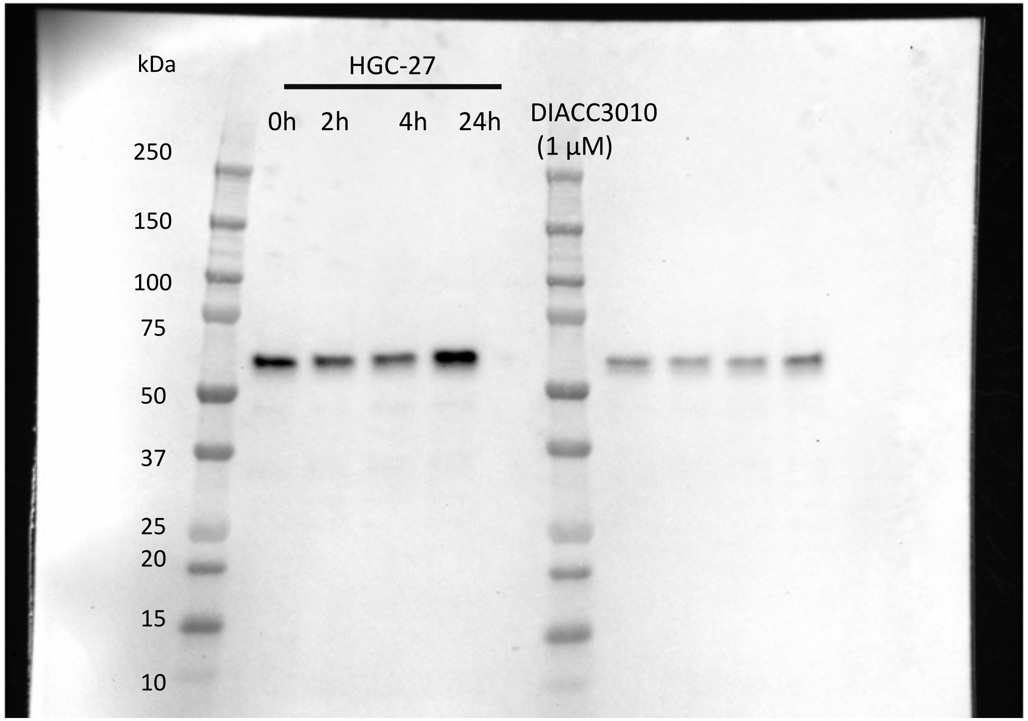

Original image
Figure 3
HGC-27

Phospho-PRAS40 (Thr246)

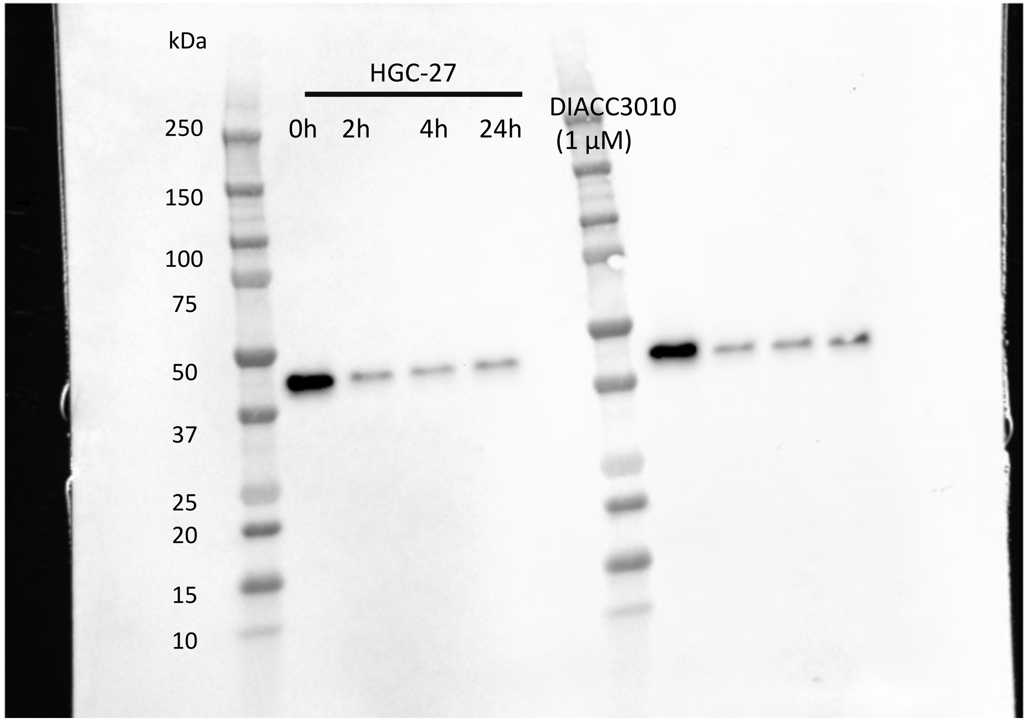

Original image
Figure 3
HGC-27

PRAS40

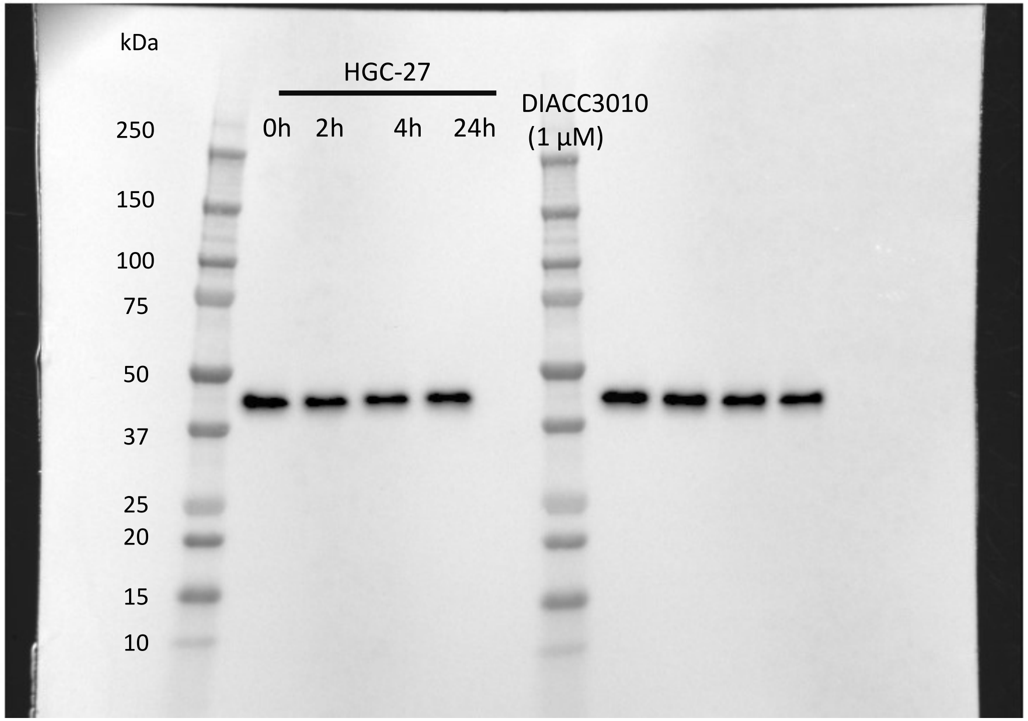

Original image
Figure 3
HGC-27

Phospho-S6 Ribosomal protein (Ser240/244)

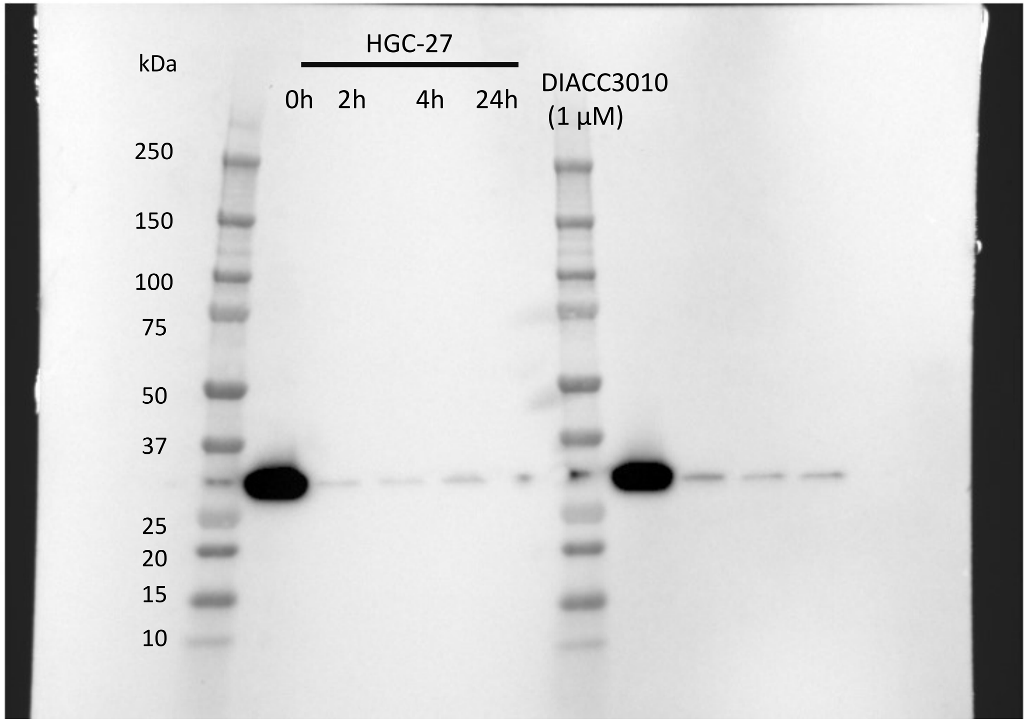

Original image
Figure 3
HGC-27

S6 Ribosomal protein

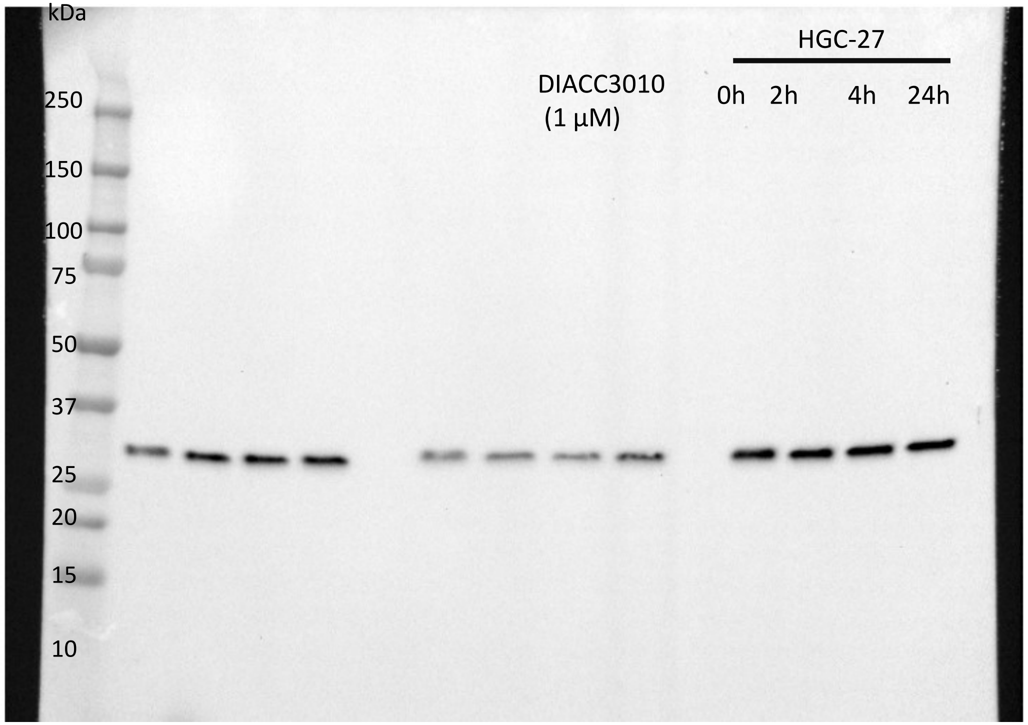

Original image
Figure 3
HGC-27

β-Actin

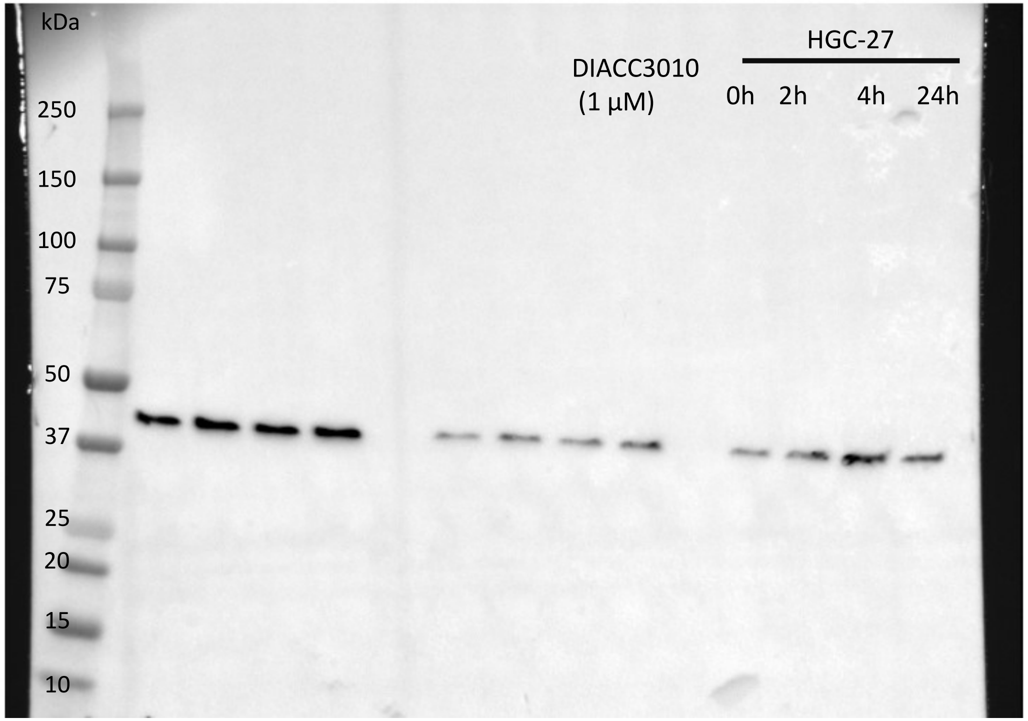

Original image
Figure 3
IM95m

Phospho-p44/42 MAPK (Erk1/2) (Thr202/Tyr204)

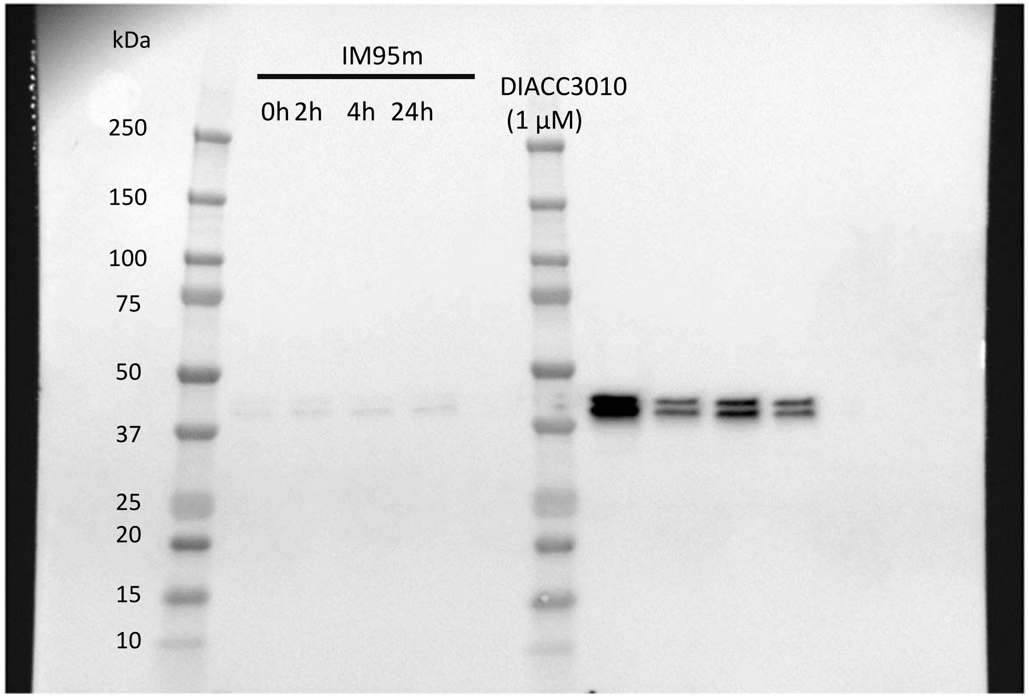

Original image
Figure 3
IM95m

P44/42 MAPK (Erk1/2)

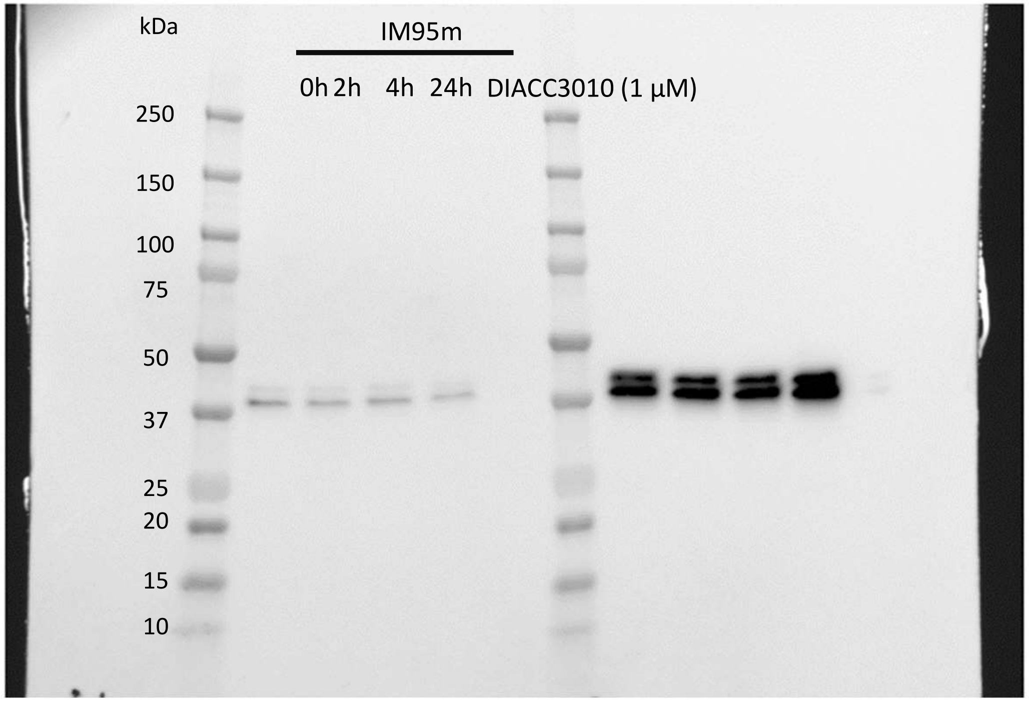

Original image
Figure 3
IM95m

Phospho-Akt (Ser473)

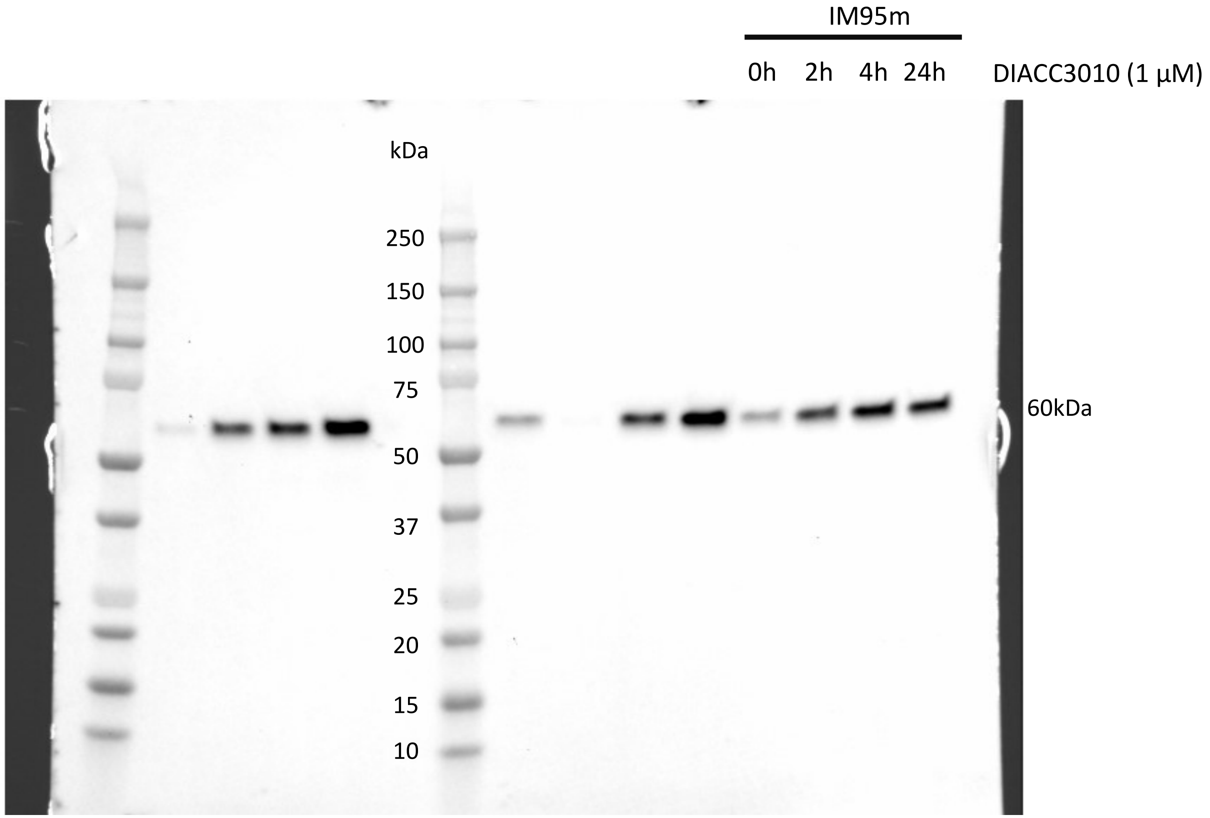

Original image
Figure 3
IM95m

Akt (pan)

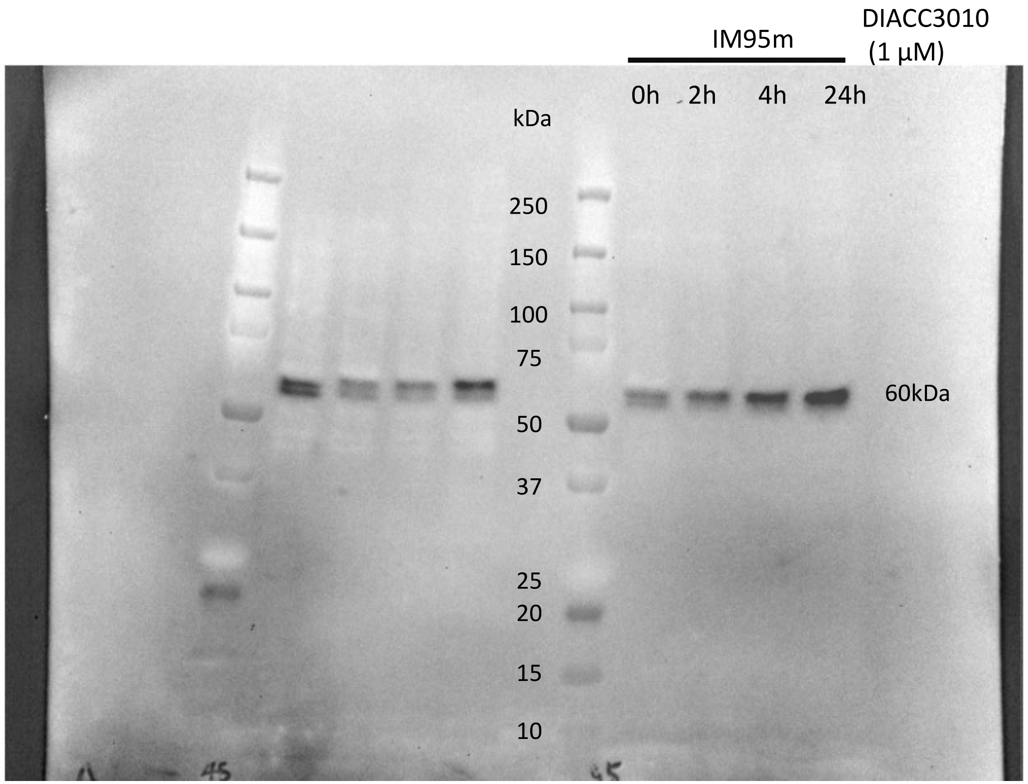

Original image
Figure 3
IM95m

Phospho-PRAS40 (Thr246)

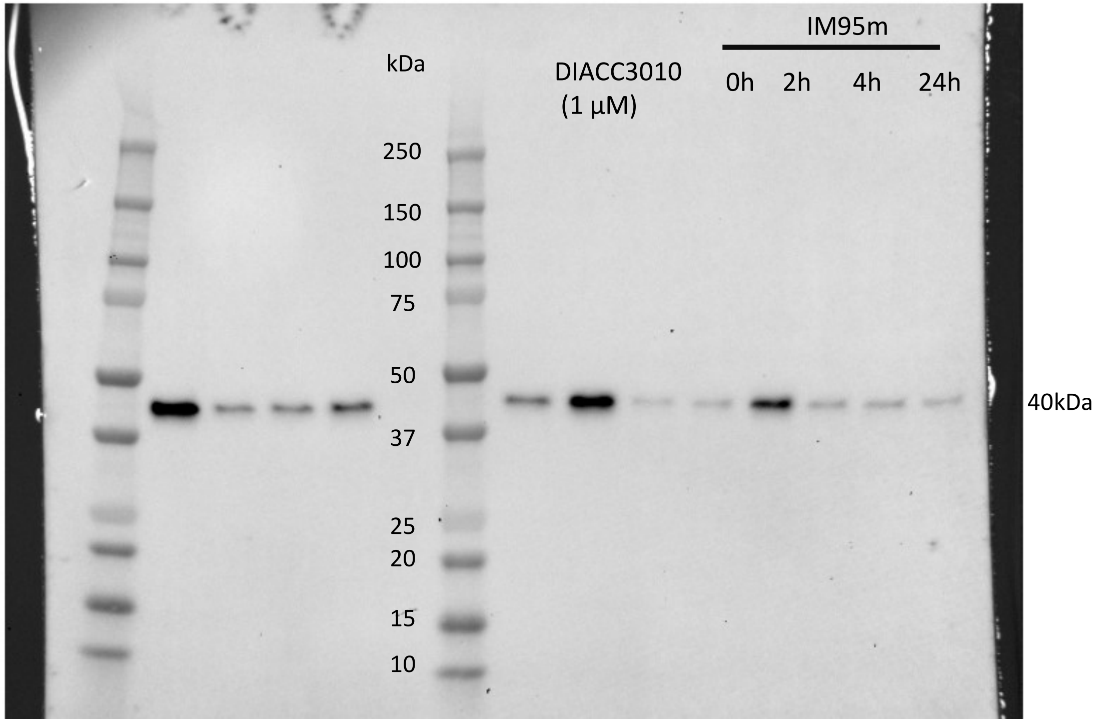

Original image
Figure 3
IM95m

PRAS40

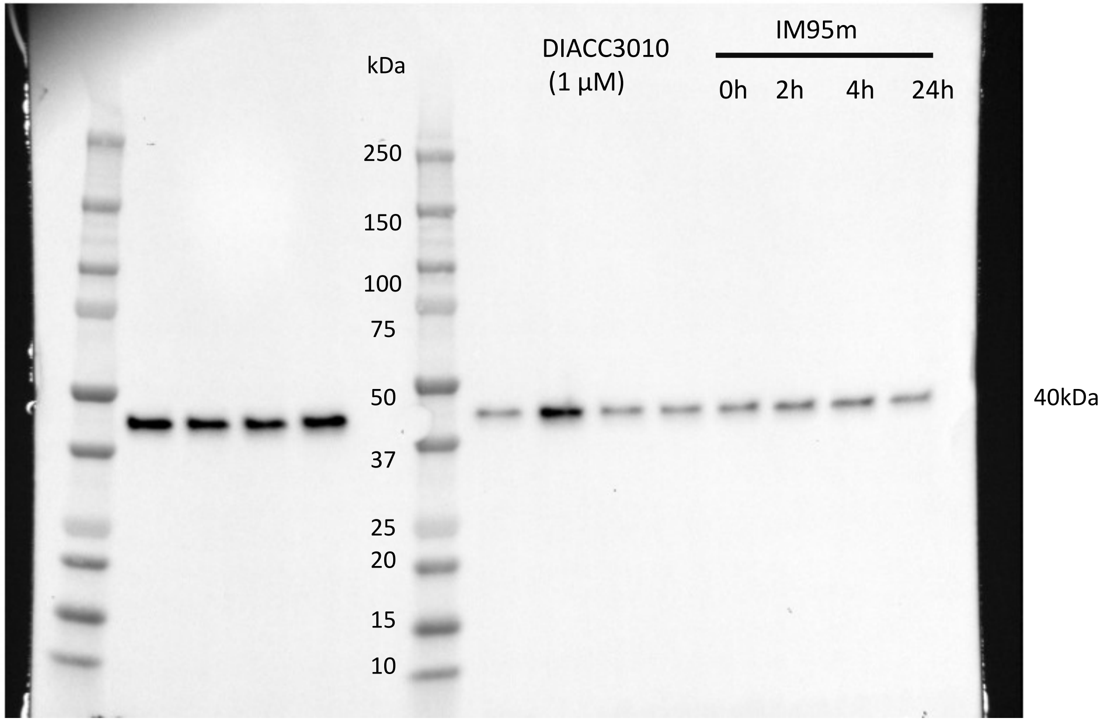

Original image
Figure 3
IM95m

Phospho-S6 Ribosomal protein (Ser240/244)

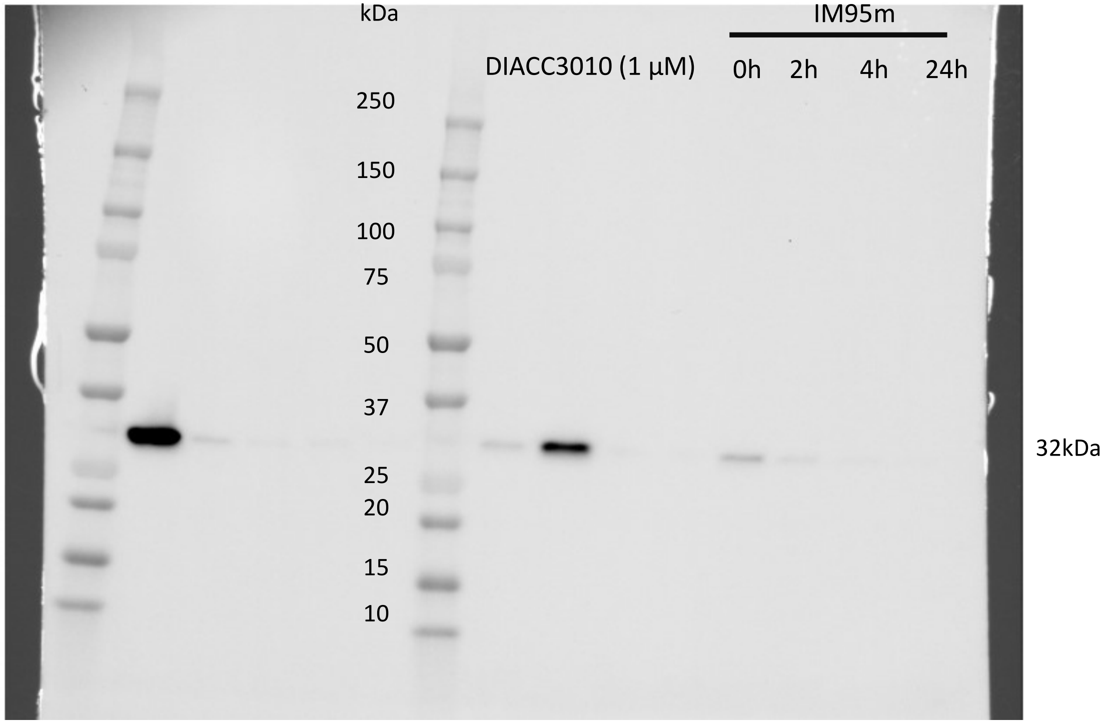

Original image
Figure 3
IM95m

S6 Ribosomal protein

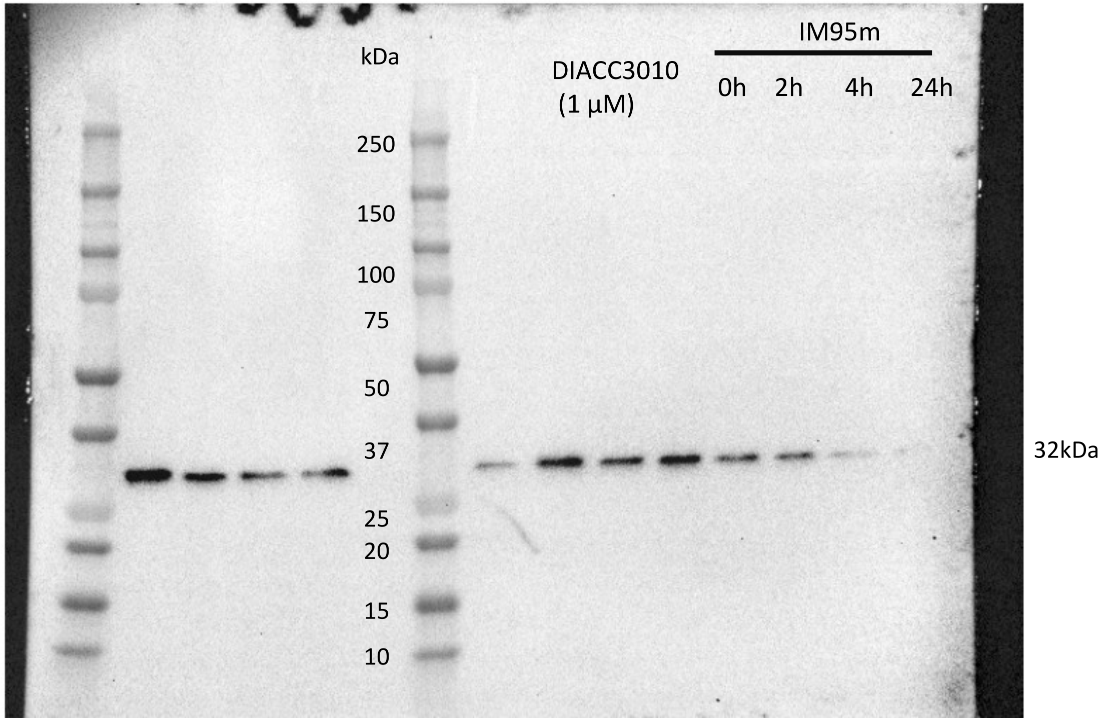

Original image
Figure 3
IM95m

β-Actin

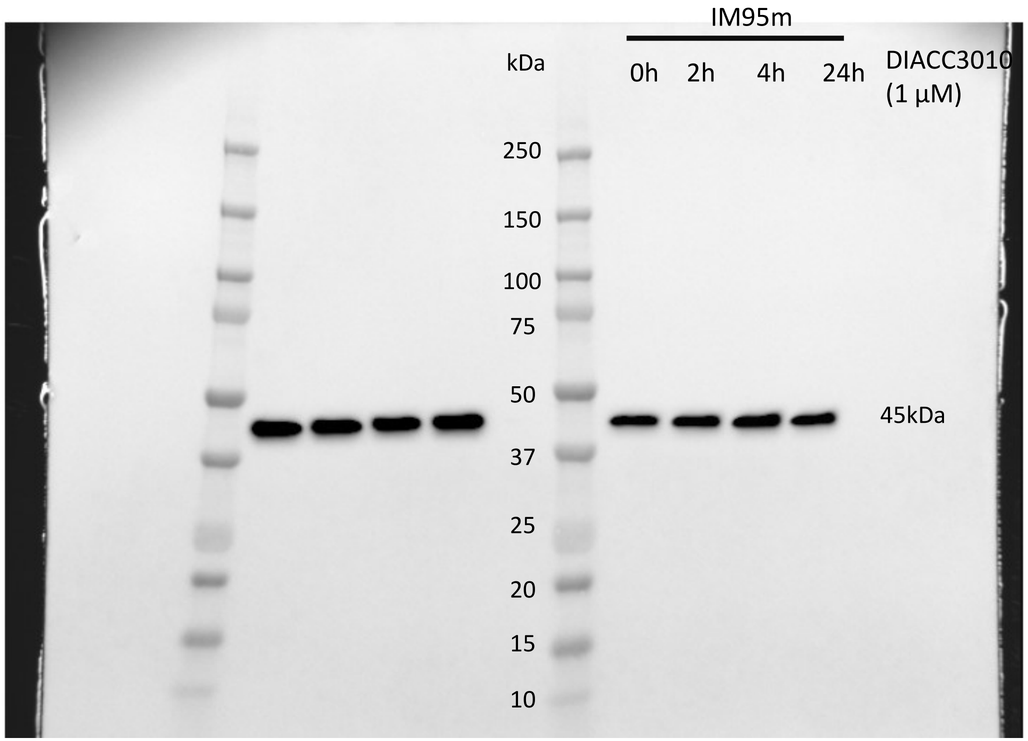

Original image
Figure 3
KE-39

Phospho-p44/42 MAPK (Erk1/2)(Thr202/Tyr204)

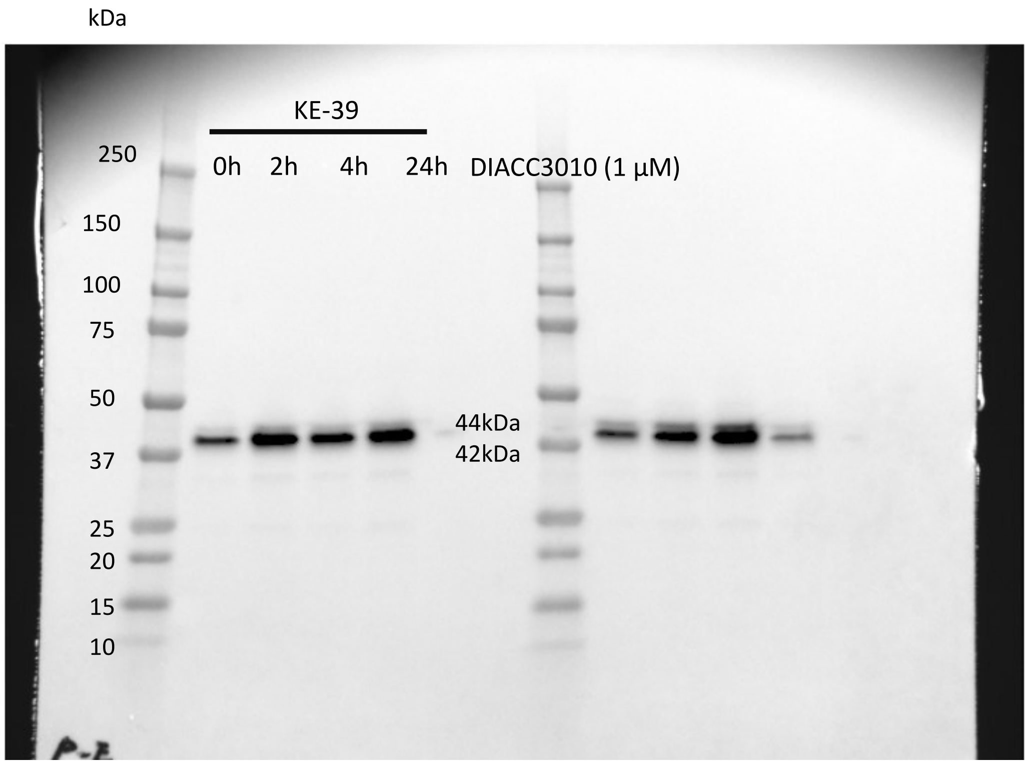

Original image
Figure 3
KE-39

P44/42 MAPK (Erk1/2)

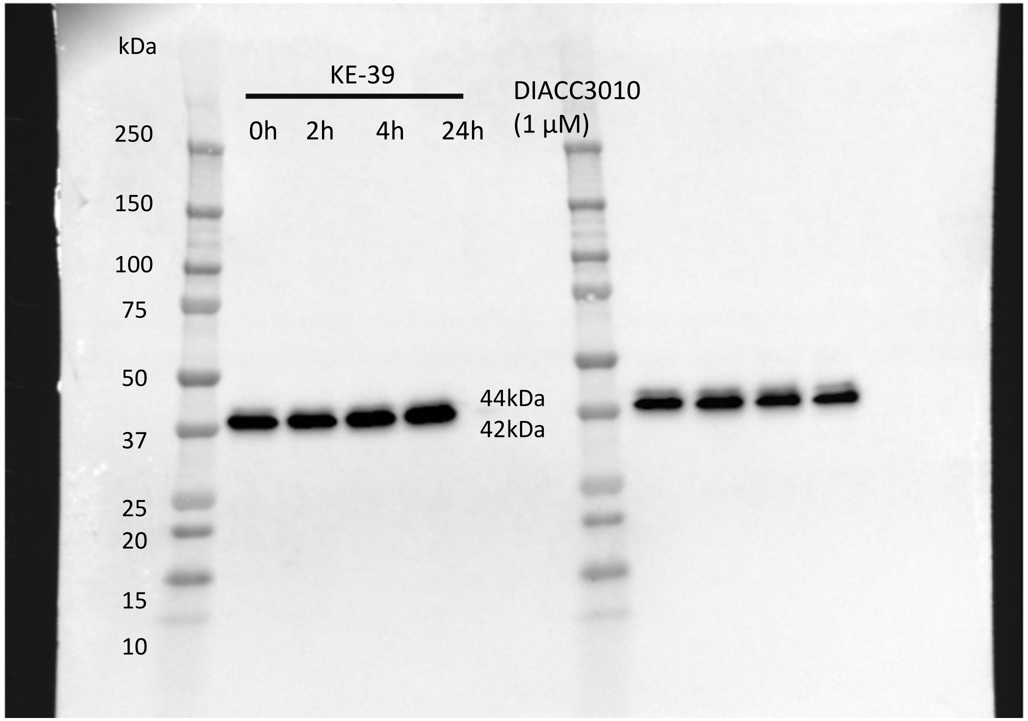

Original image
Figure 3
KE-39

Phospho-Akt (Ser473)

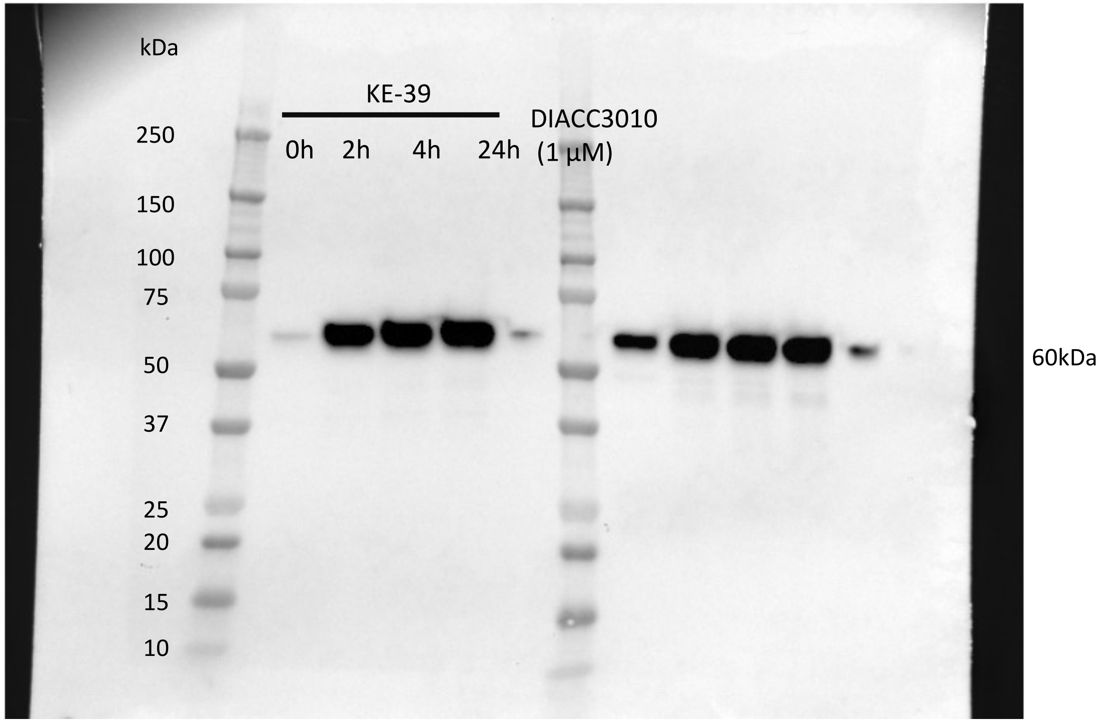

Original image
Figure 3
KE-39

Akt (pan)

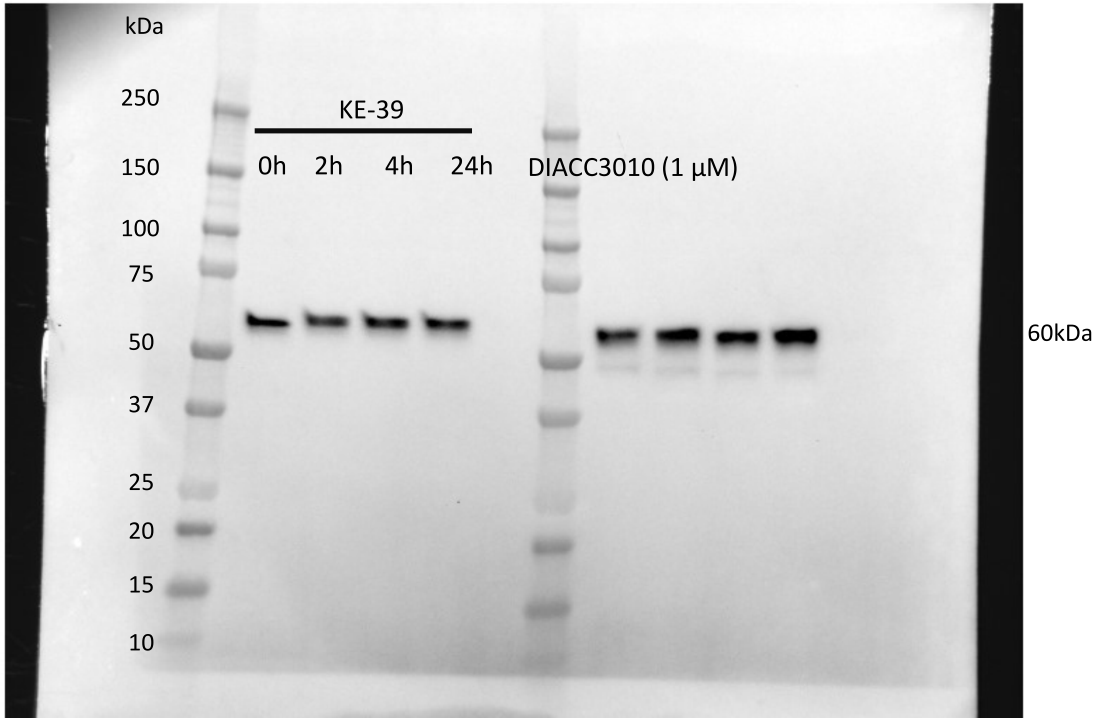

Original image
Figure 3
KE-39

Phospho-PRAS40 (Thr246)

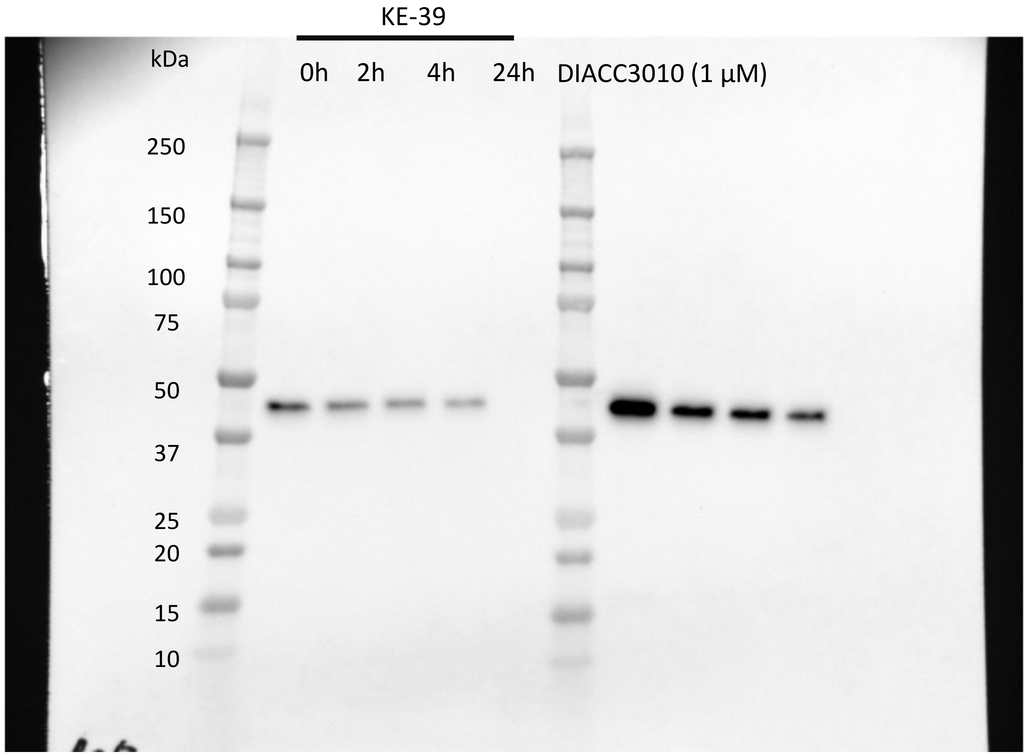

Original image
Figure 3
KE-39

PRAS40

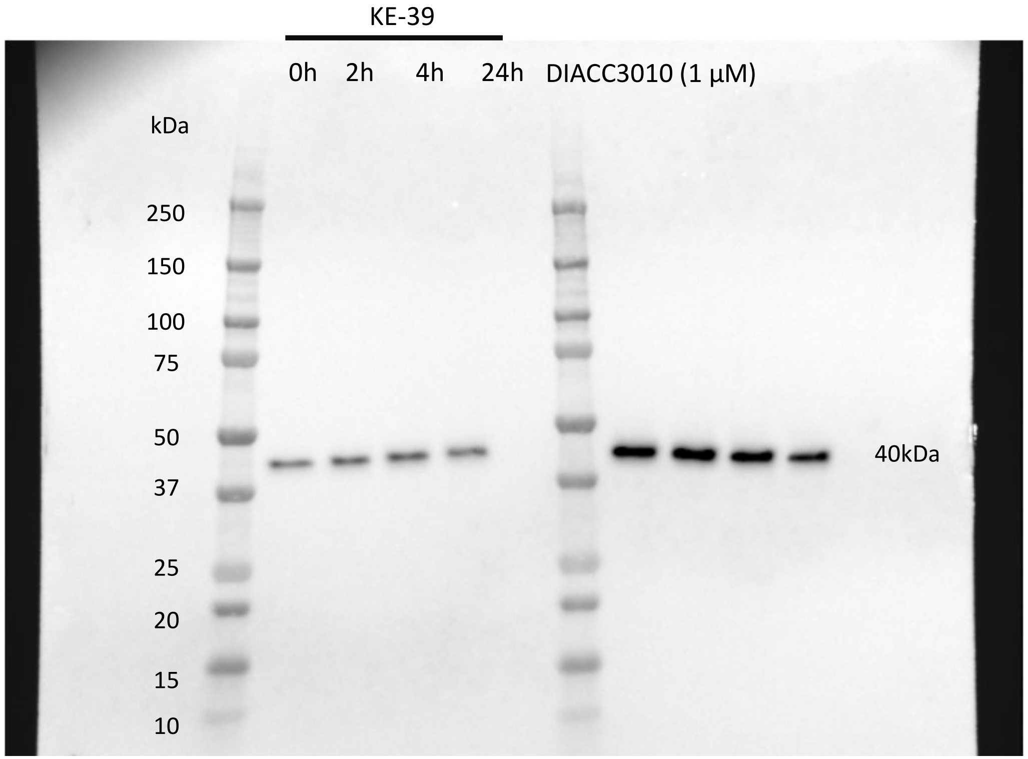

Original image
Figure 3
KE-39

Phospho-S6 Ribosomal protein (Ser240/244)

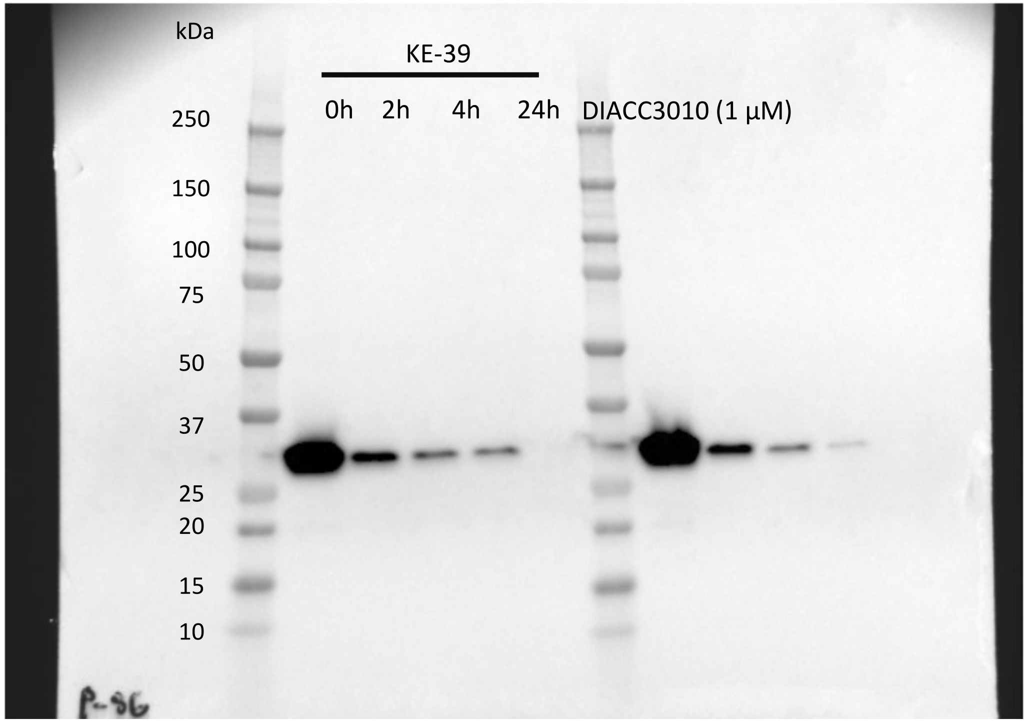

32kDa

Original image
Figure 3
KE-39

S6 Ribosomal protein

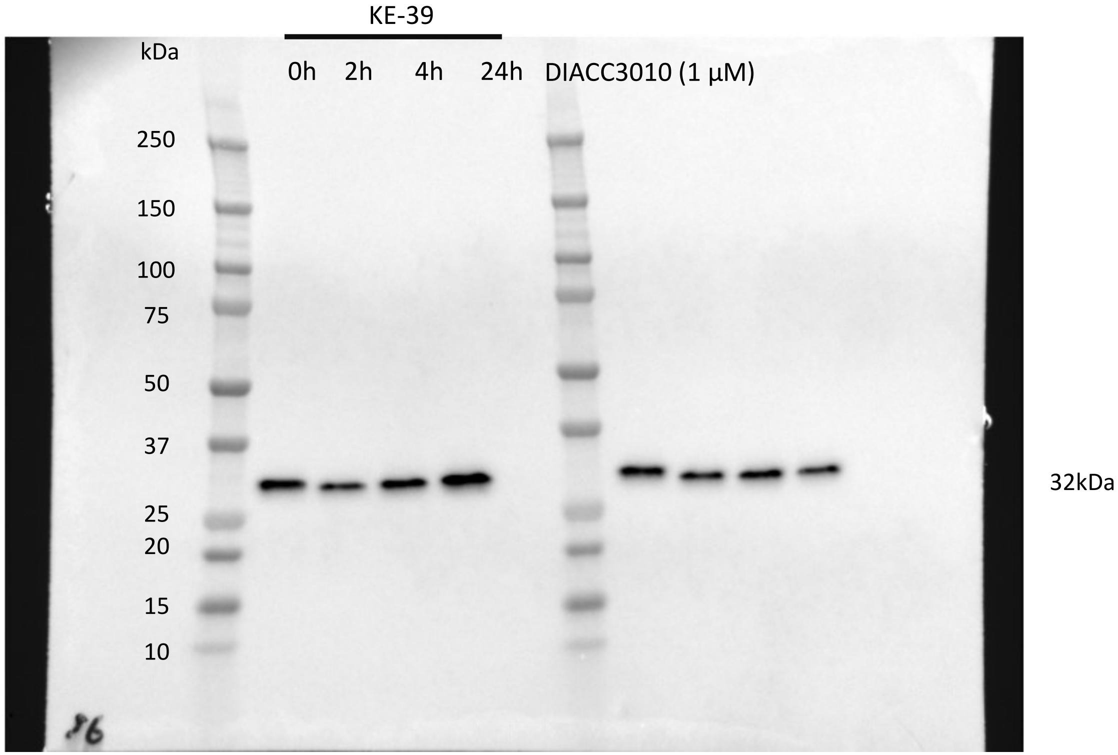

Original image
Figure 3
KE-39

β-Actin

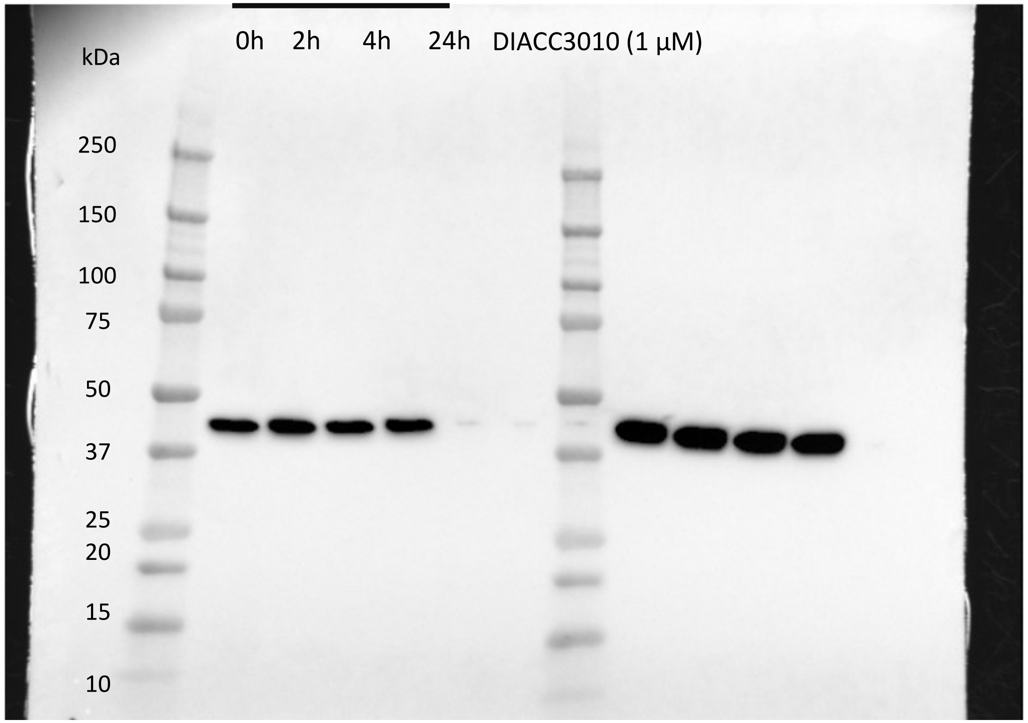

Original image
Figure 3
NCI-N87

Phospho-p44/42 MAPK (Erk1/2)(Thr202/Tyr204)

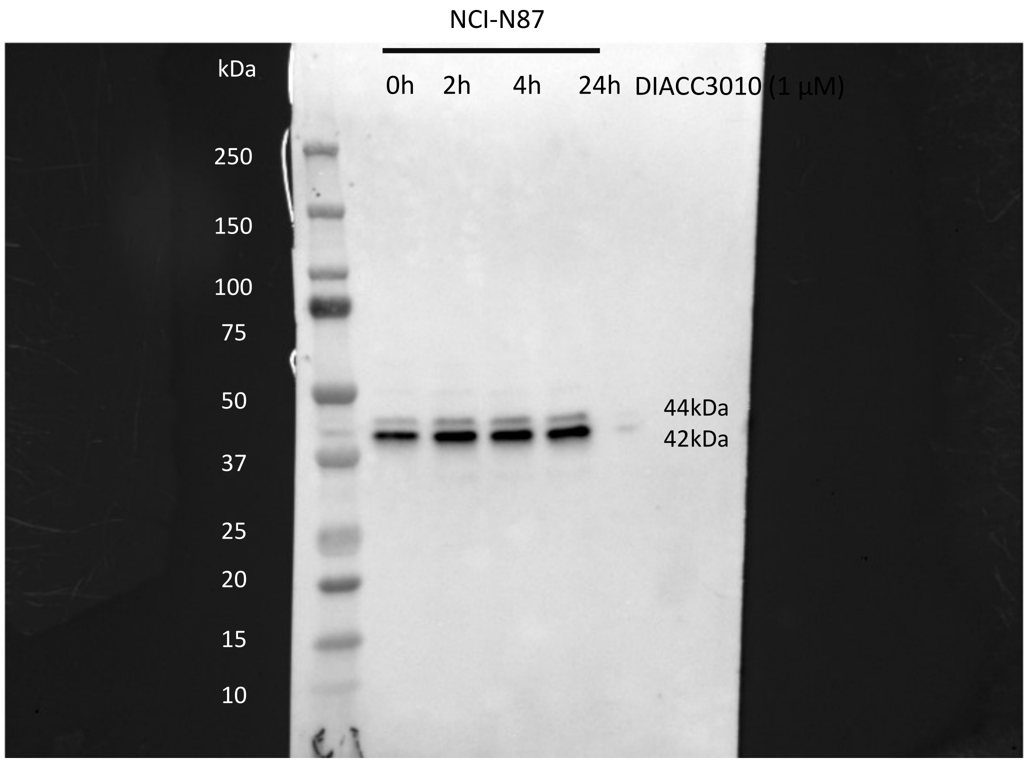

Original image
Figure 3
NCI-N87

P44/42 MAPK (Erk1/2)

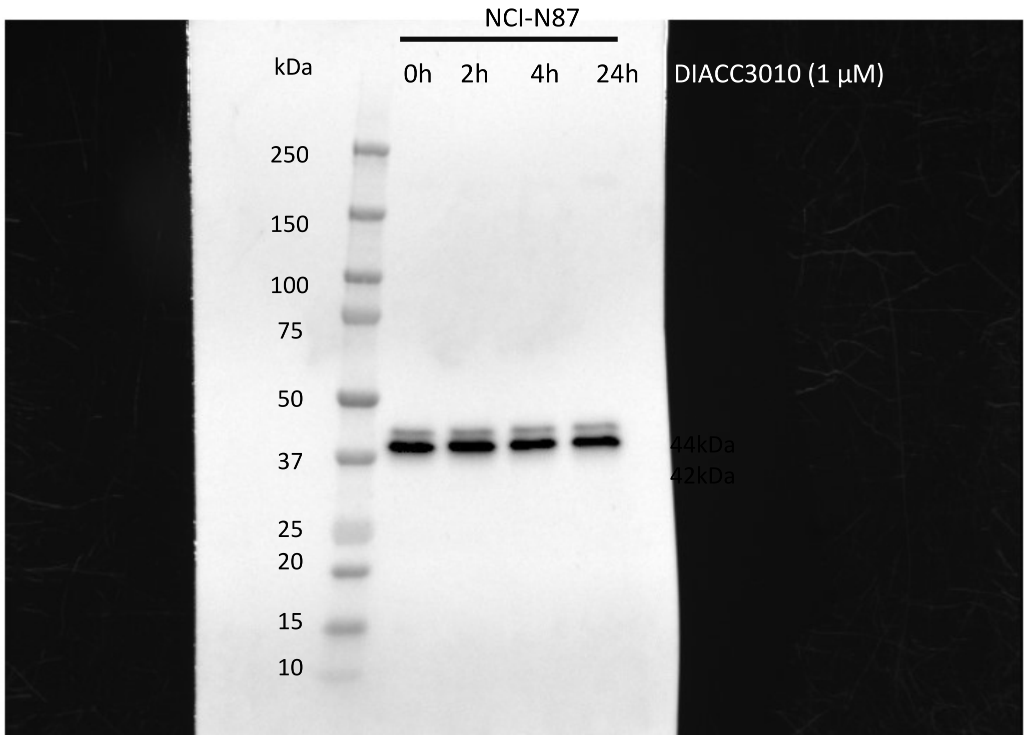

Original image
Figure 3
NCI-N87

Phospho-Akt (Ser473)

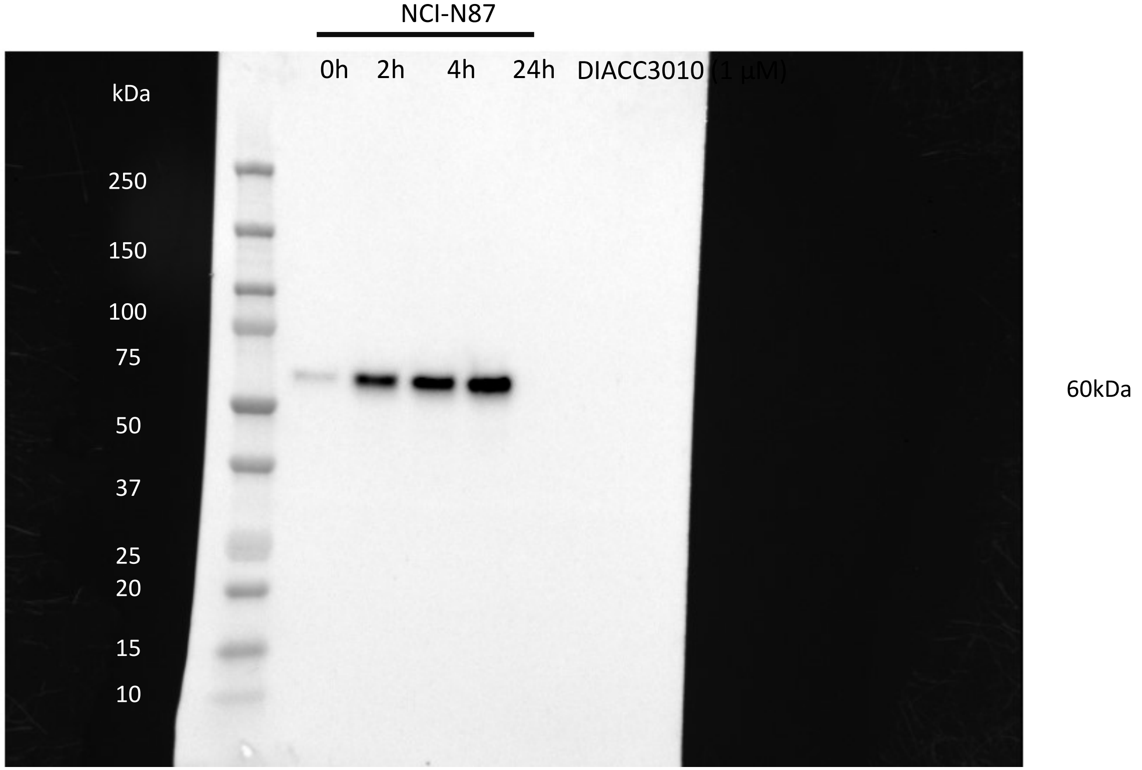

Original image
Figure 3
NCI-N87

Akt (pan)

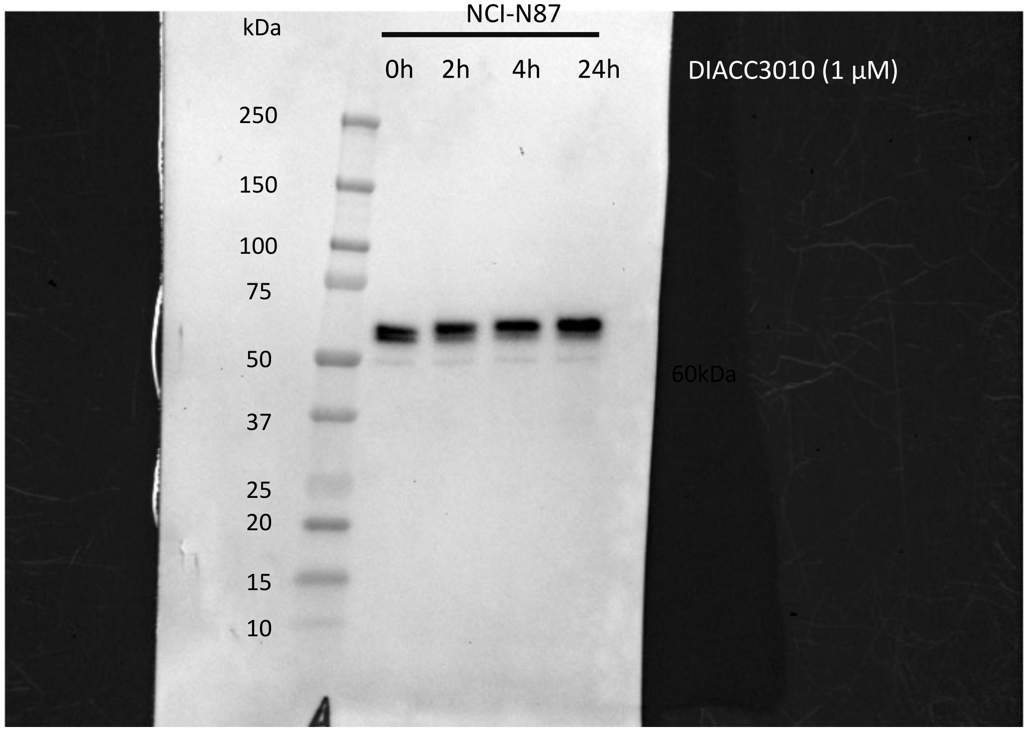

Original image
Figure 3
NCI-N87

Phospho-PRAS40 (Thr246)

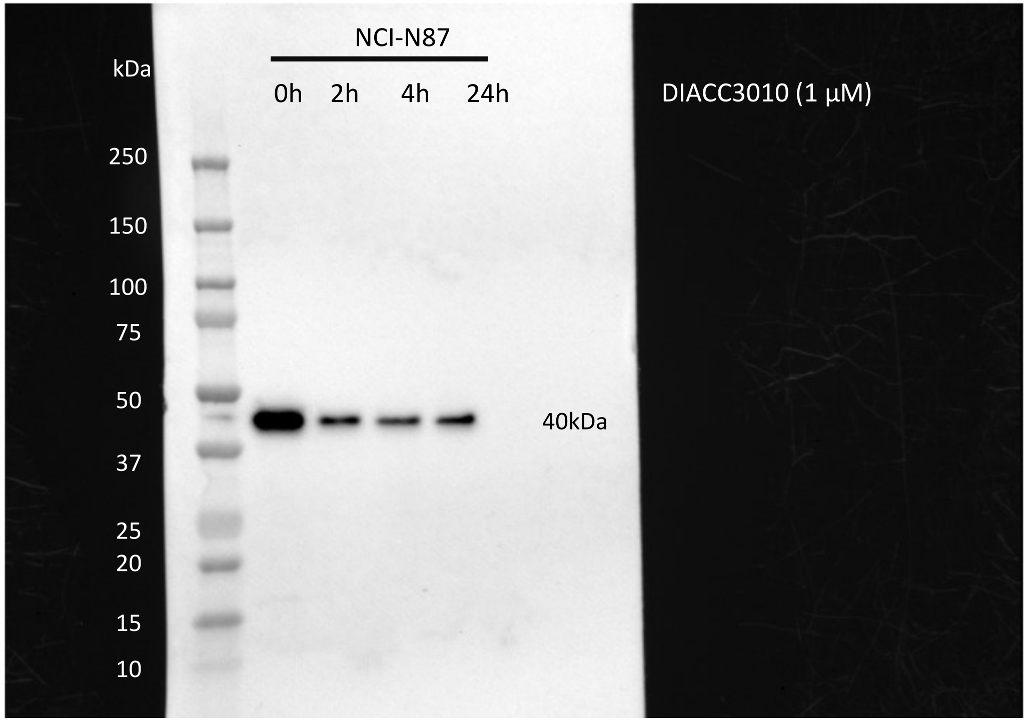

Original image
Figure 3
NCI-N87

PRAS40

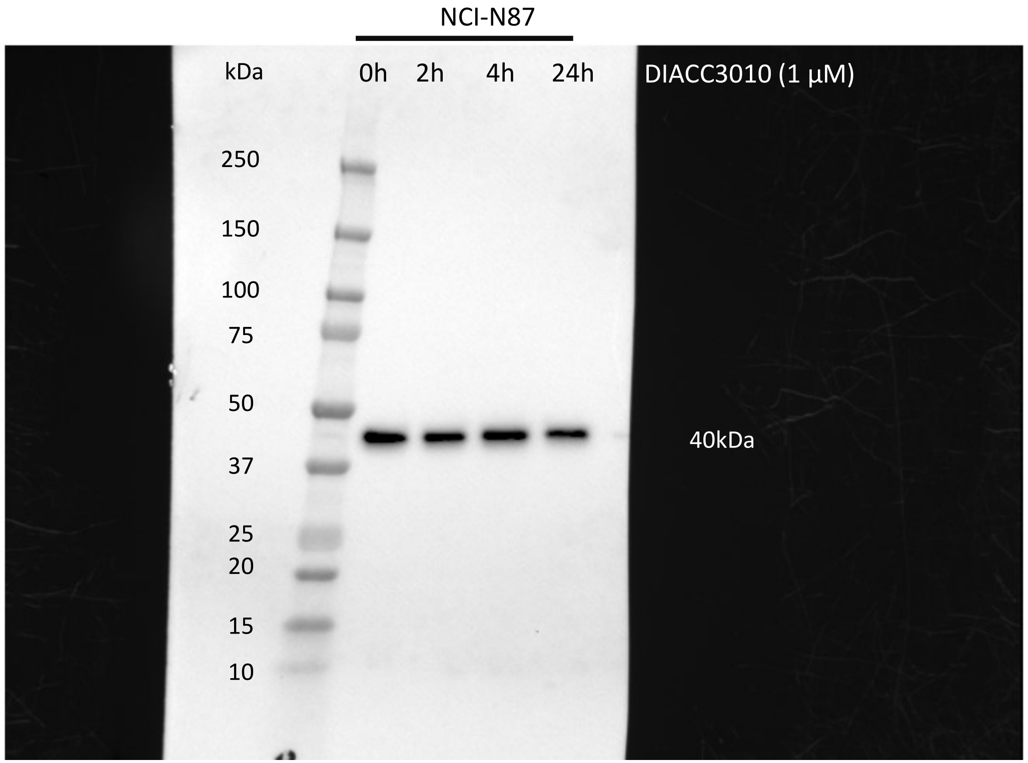

Original image
Figure 3
NCI-N87

Phospho-S6 Ribosomal protein (Ser240/244)

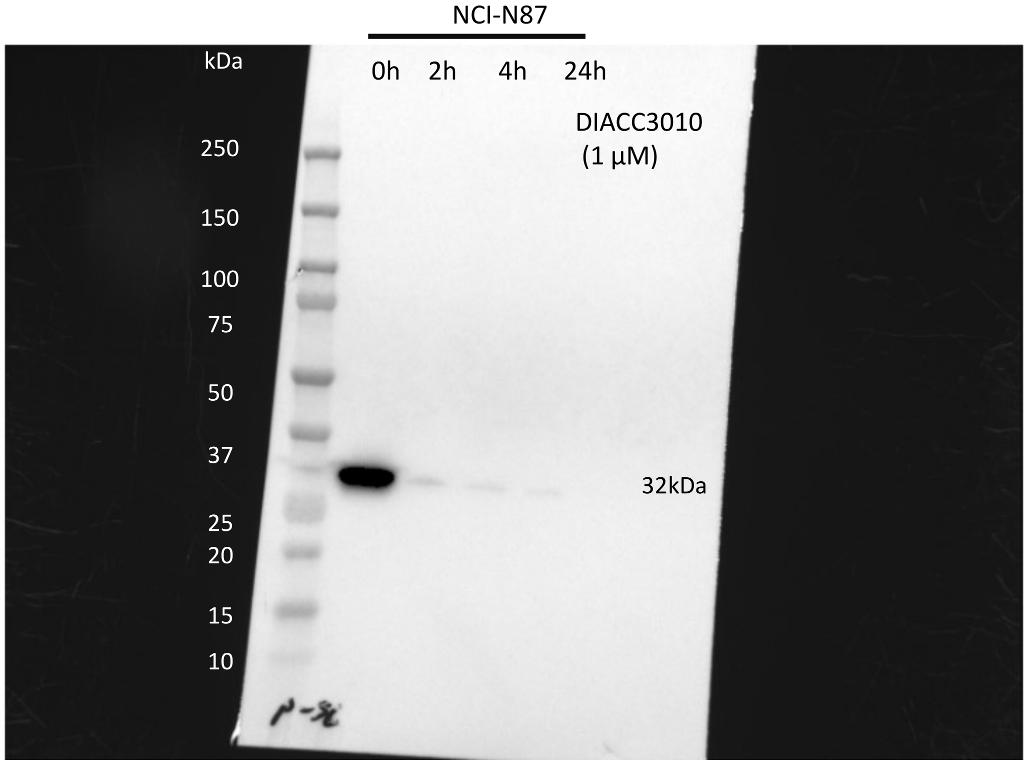

Original image
Figure 3
NCI-N87

S6 Ribosomal protein

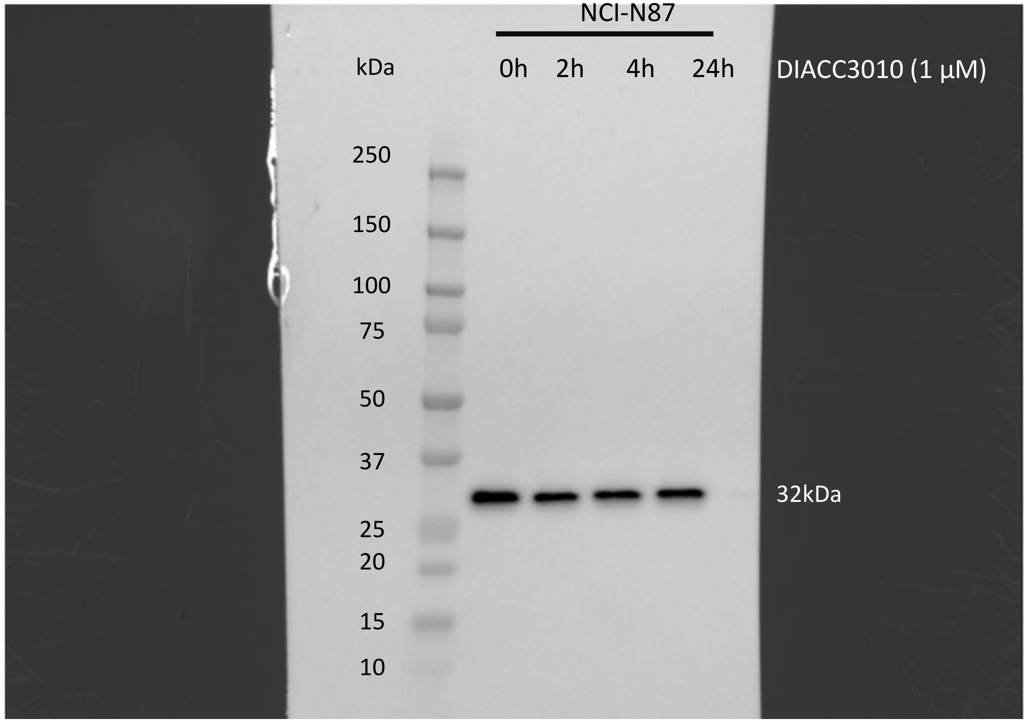

Original image
Figure 3
NCI-N87

β-Actin

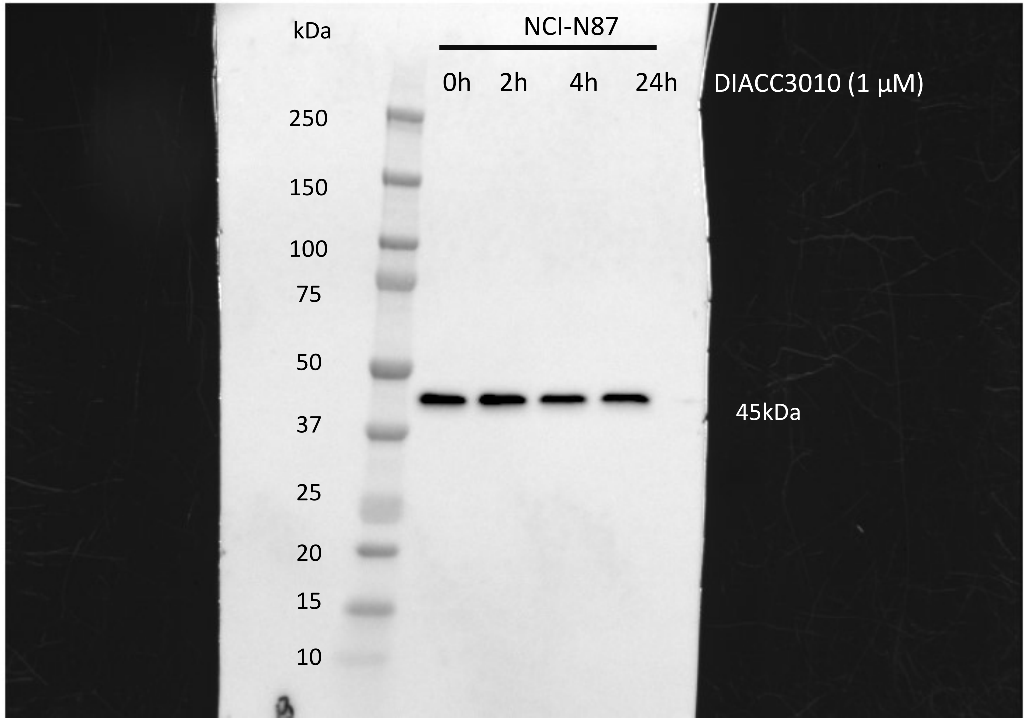

Original image
Figure 3
MKN-1

Phospho-p44/42 MAPK (Erk1/2)(Thr202/Tyr204)

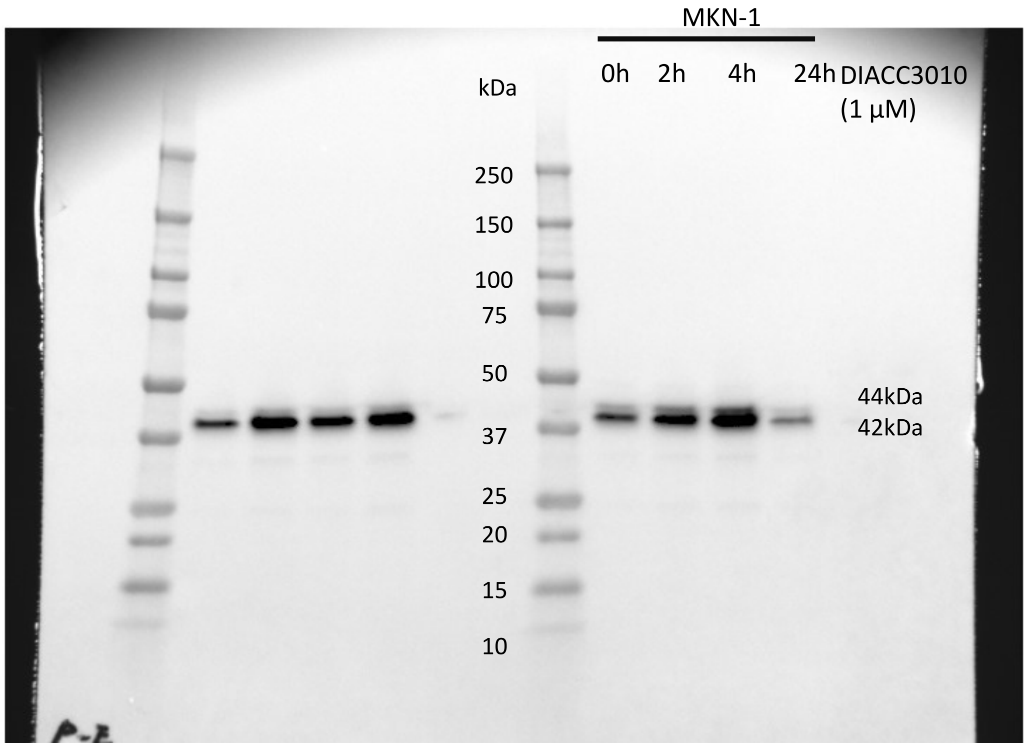

Original image
Figure 3
MKN-1

P44/42 MAPK (Erk1/2)

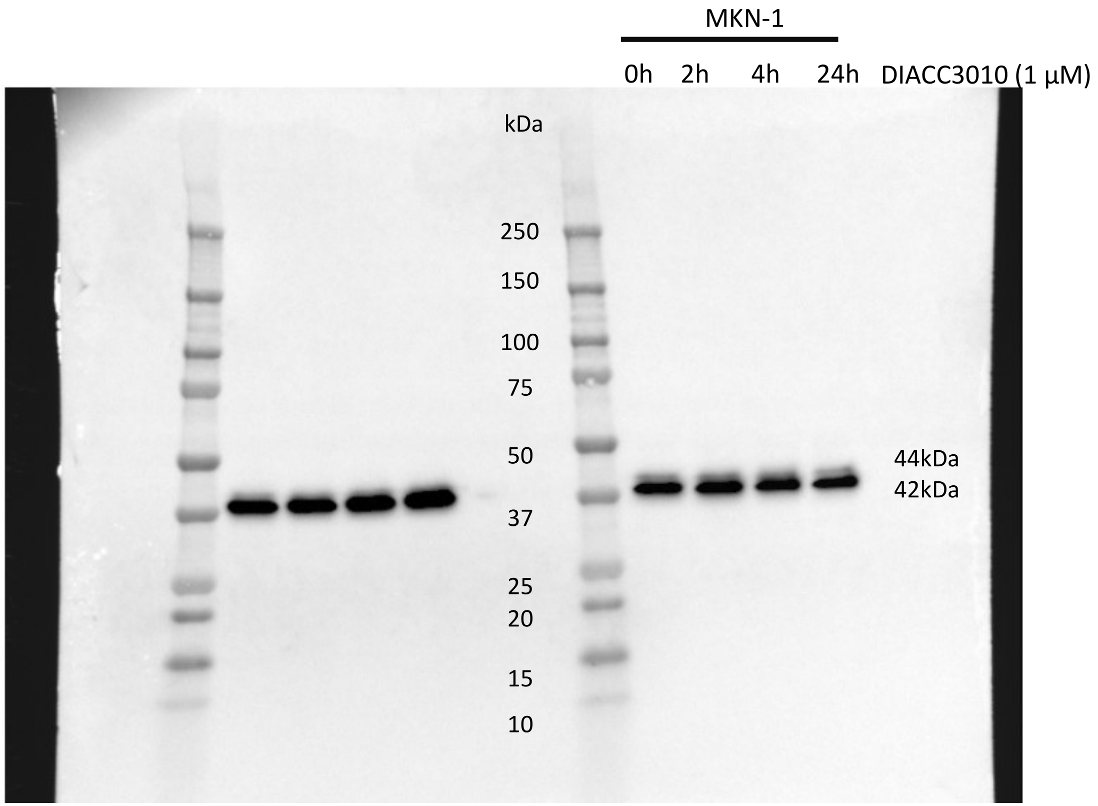

Original image
Figure 3
MKN-1

Phospho-Akt (Ser473)

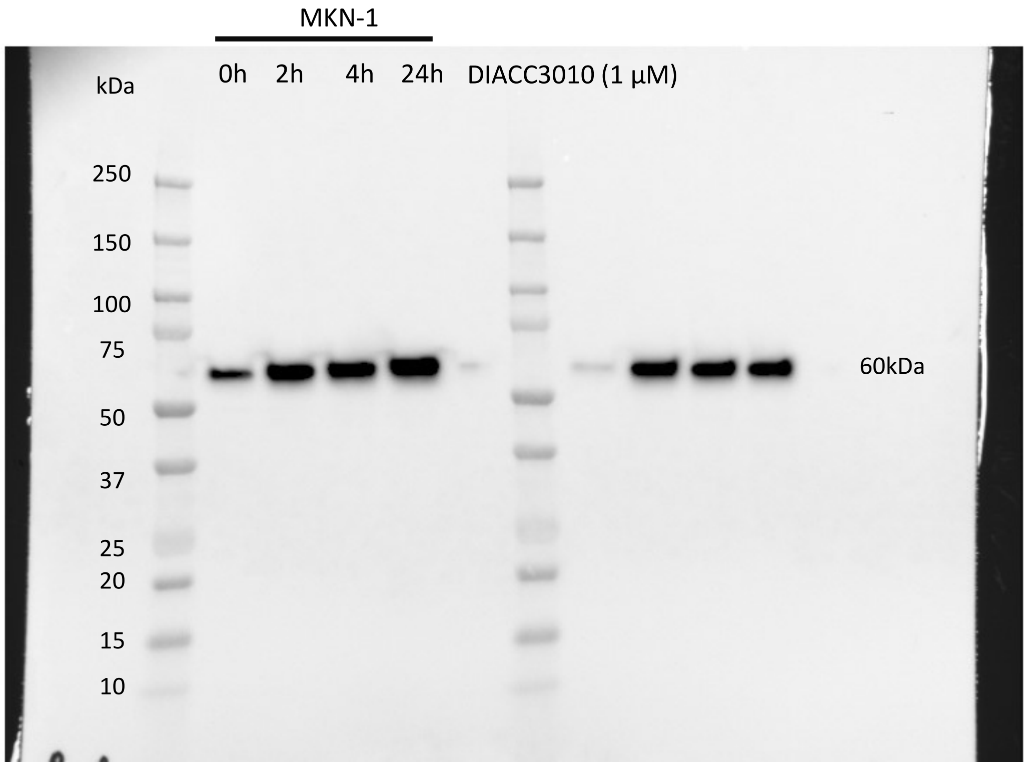

Original image
Figure 3
MKN-1

Akt (pan)

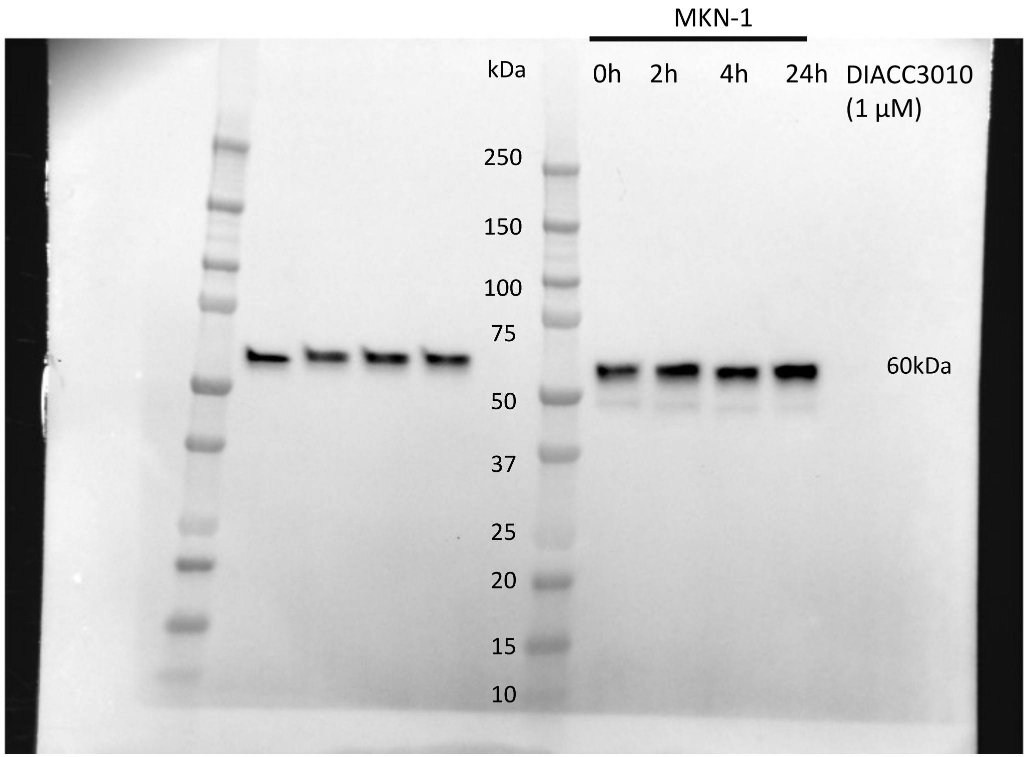

Original image
Figure 3
MKN-1

Phospho-PRAS40 (Thr246)

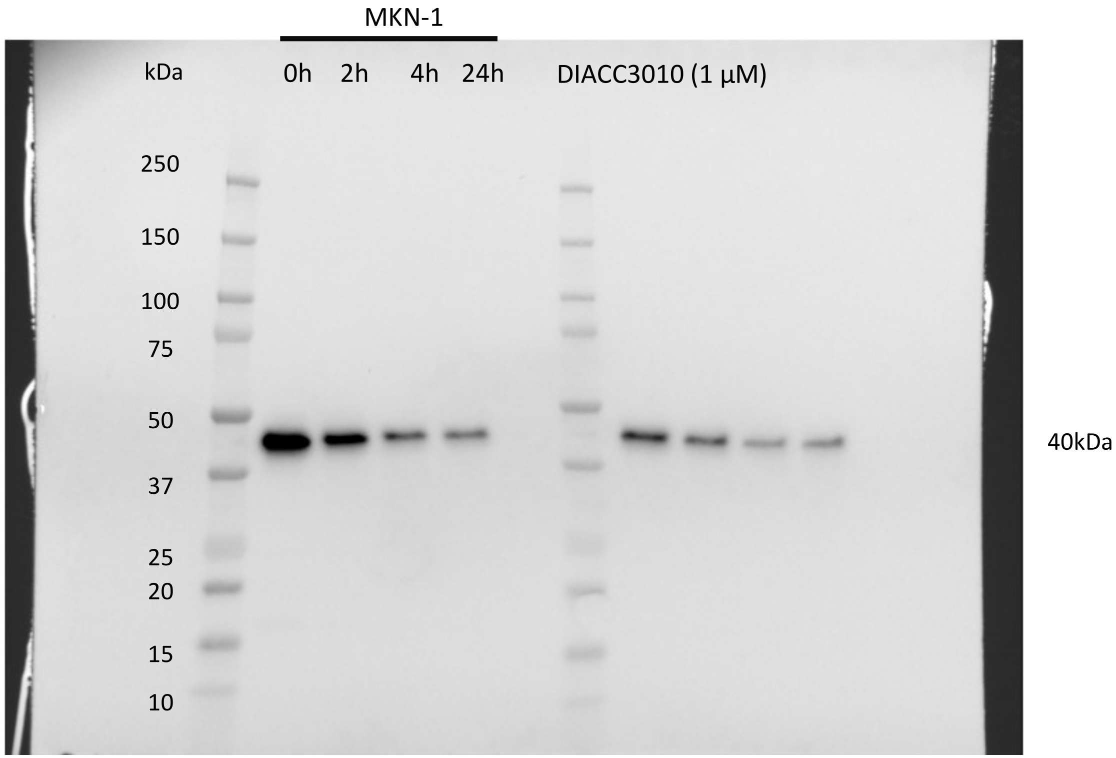

Original image
Figure 3
MKN-1

PRAS40

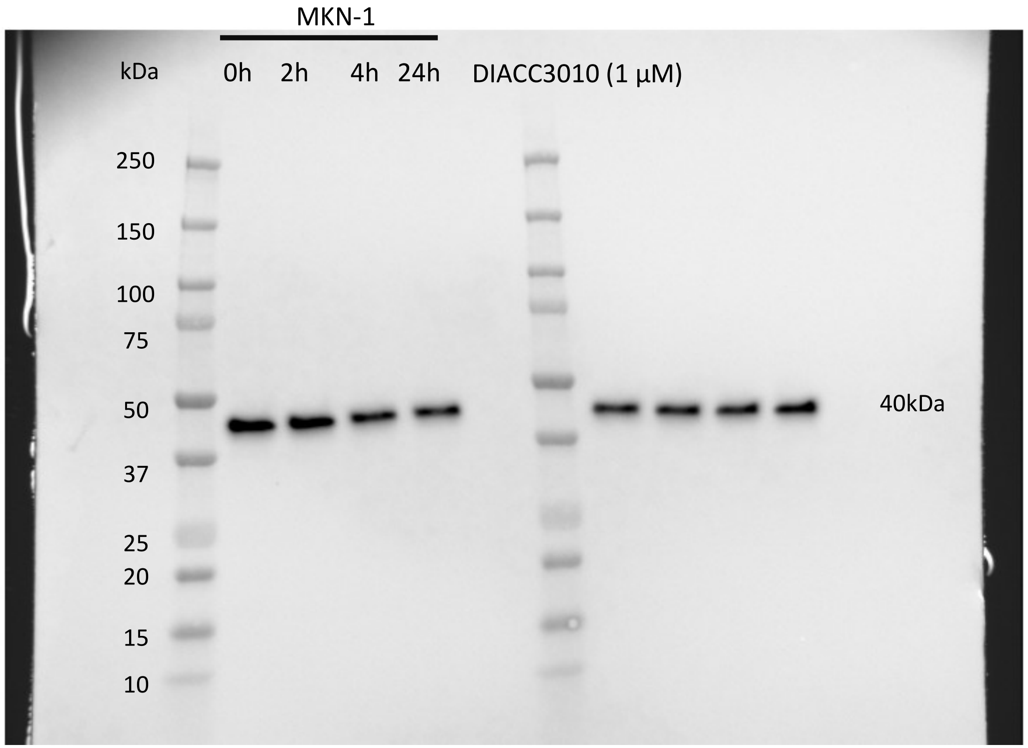

Original image
Figure 3
MKN-1

Phospho-S6 Ribosomal protein (Ser240/244)

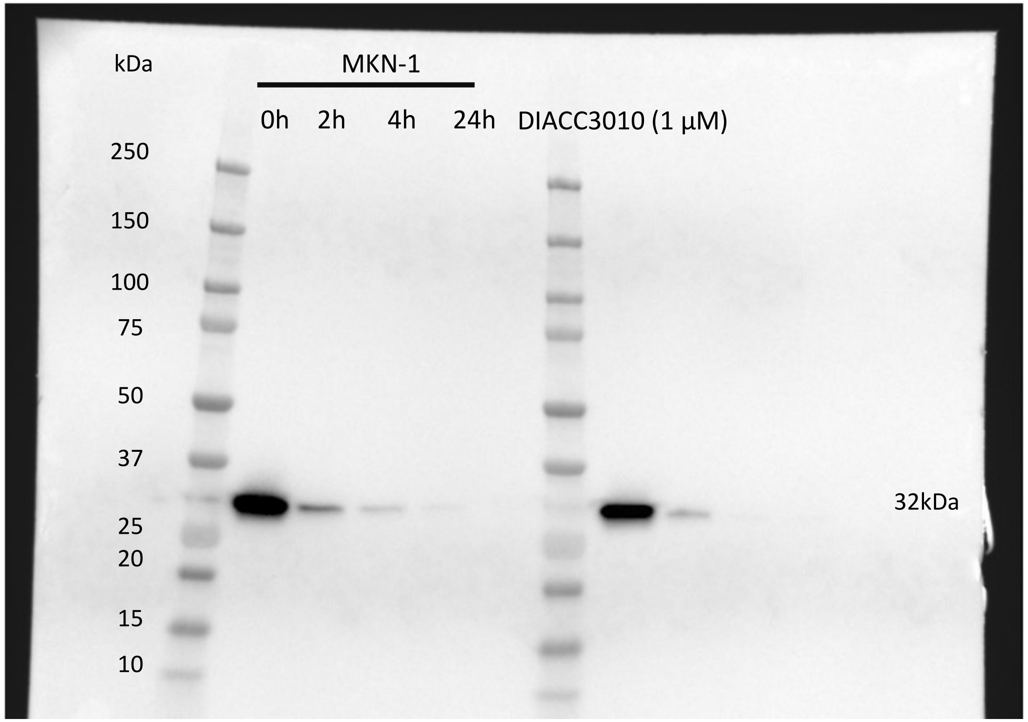

Original image
Figure 3
MKN-1

S6 Ribosomal protein

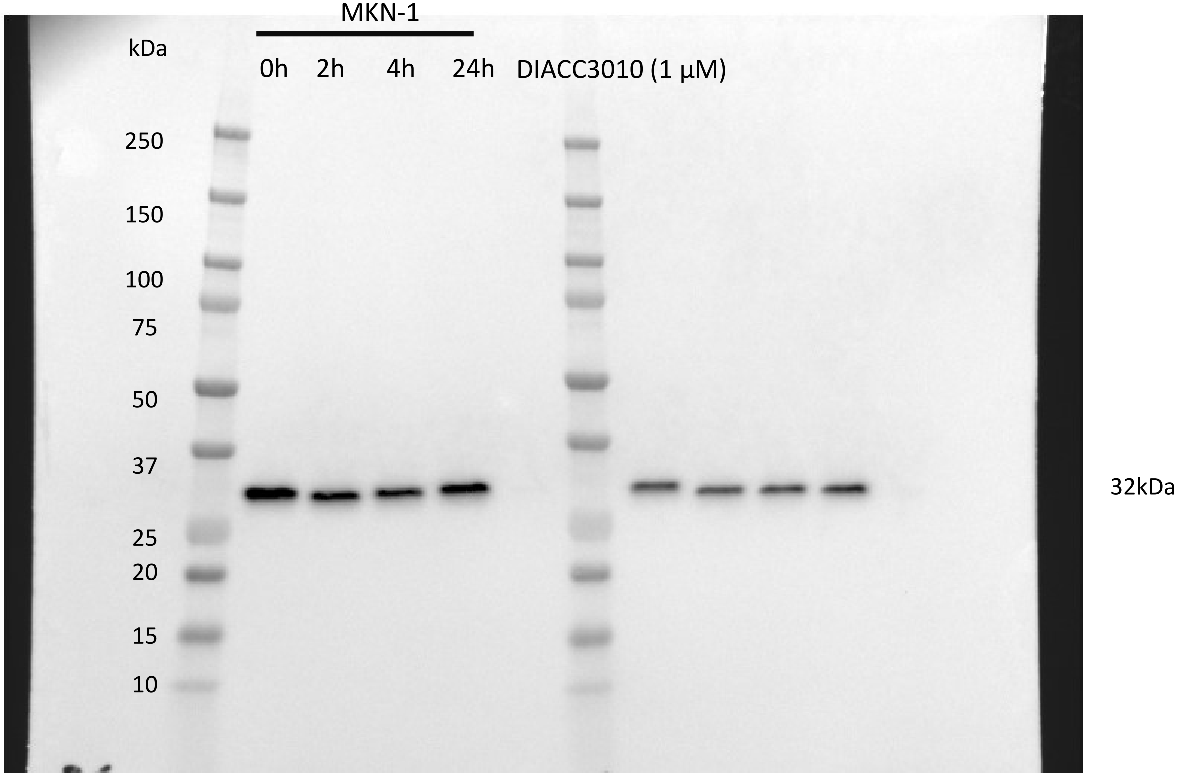

Original image
Figure 3
MKN-1

β-Actin

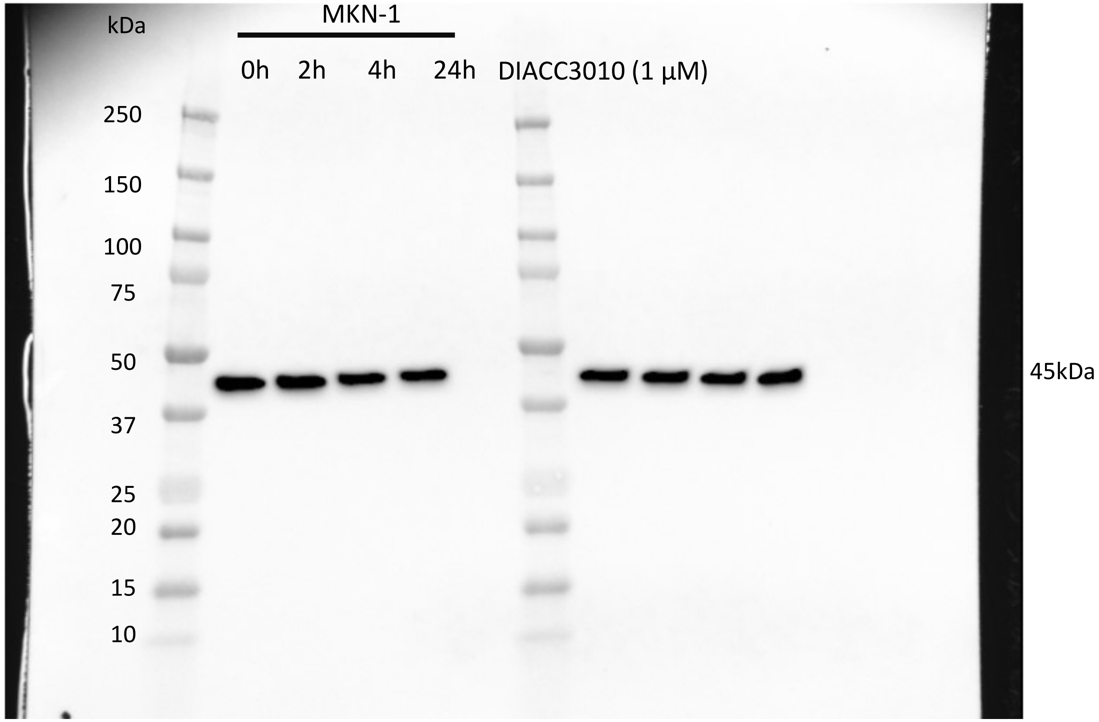

Original image
Figure 3
MKN-7

Phospho-p44/42 MAPK (Erk1/2)(Thr202/Tyr204)

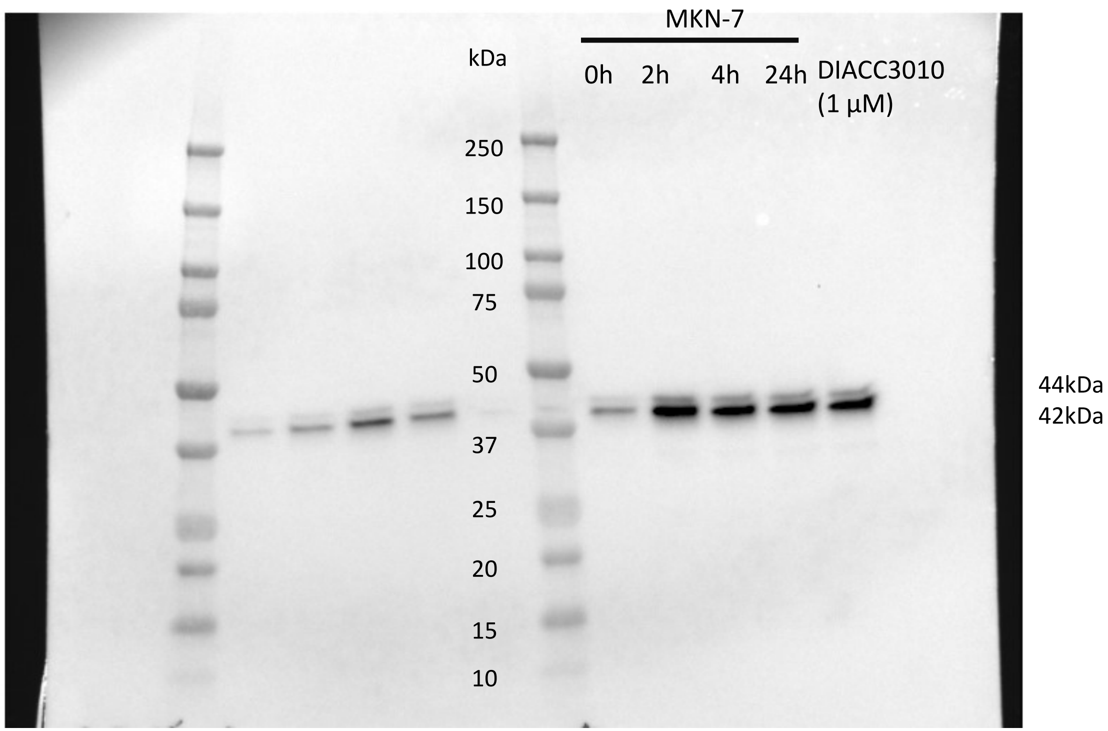

Original image
Figure 3
MKN-7

P44/42 MAPK (Erk1/2)

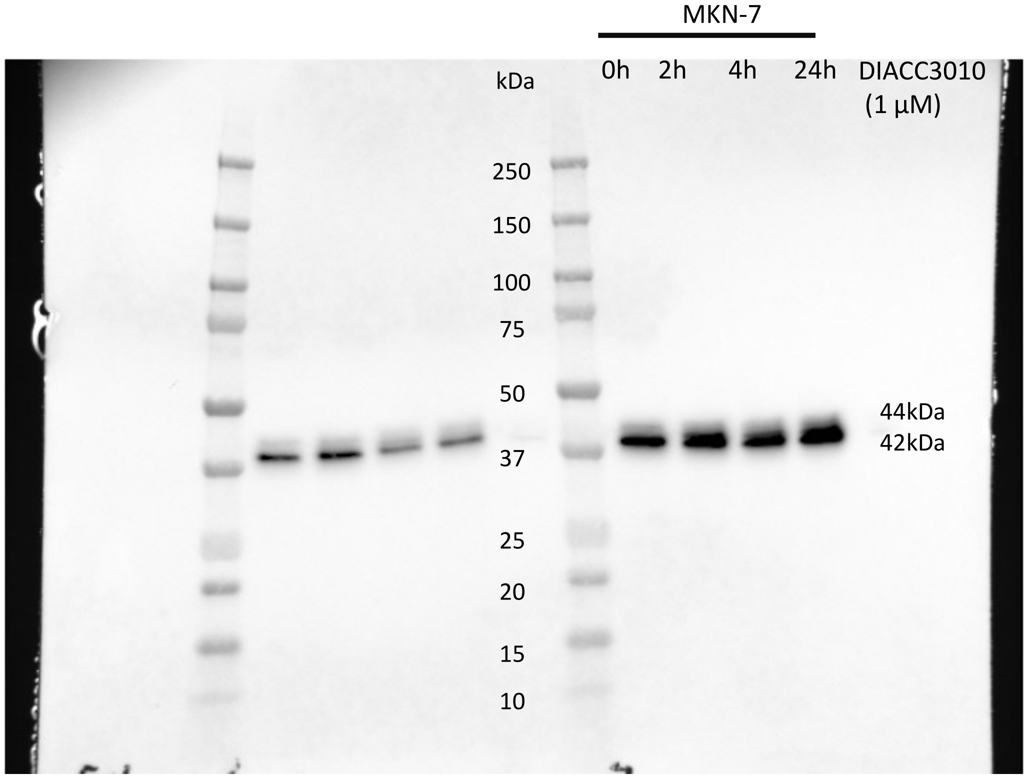

Original image
Figure 3
MKN-7

Phospho-Akt (Ser473)

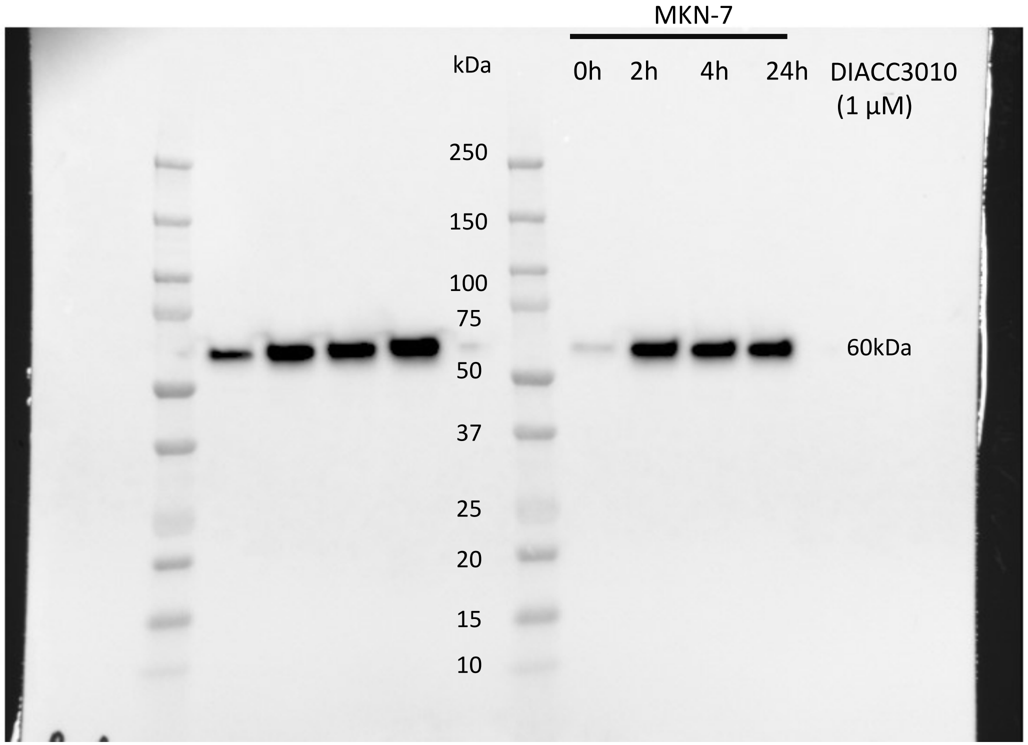

Original image
Figure 3
MKN-7

Akt (pan)

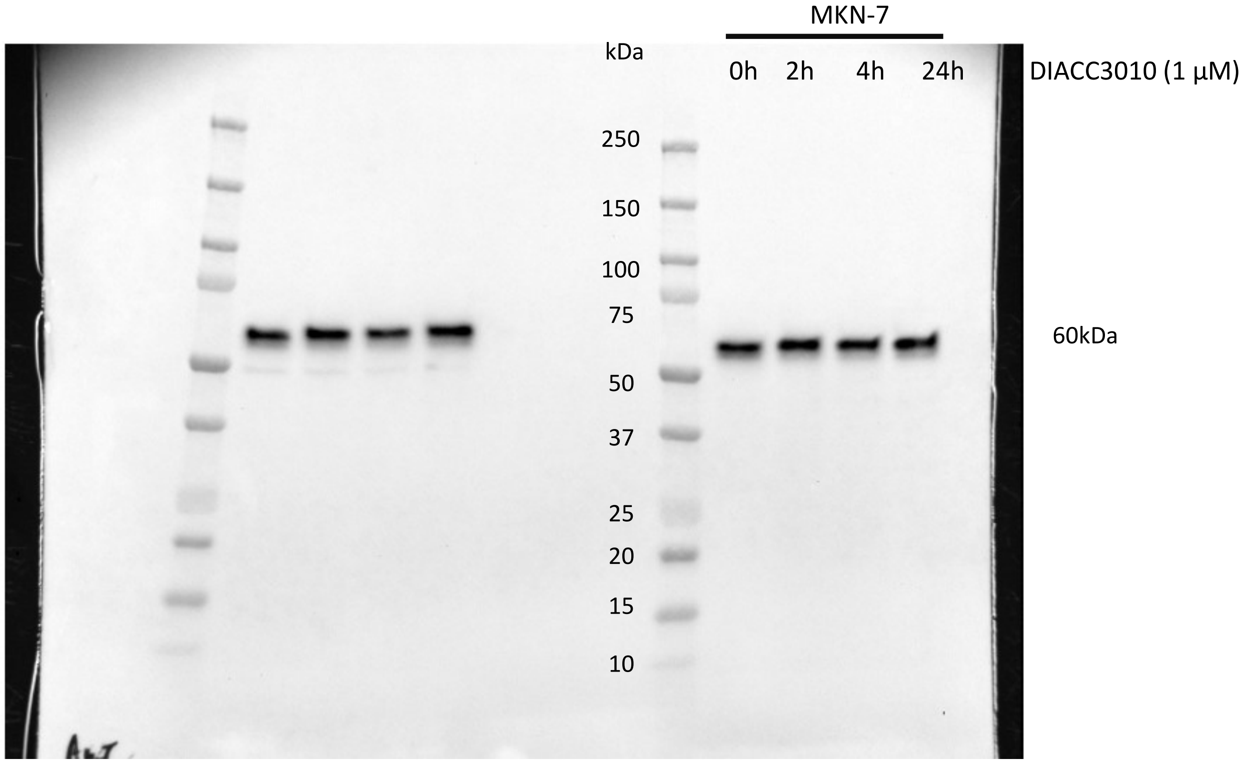

Original image
Figure 3
MKN-7

Phospho-PRAS40 (Thr246)

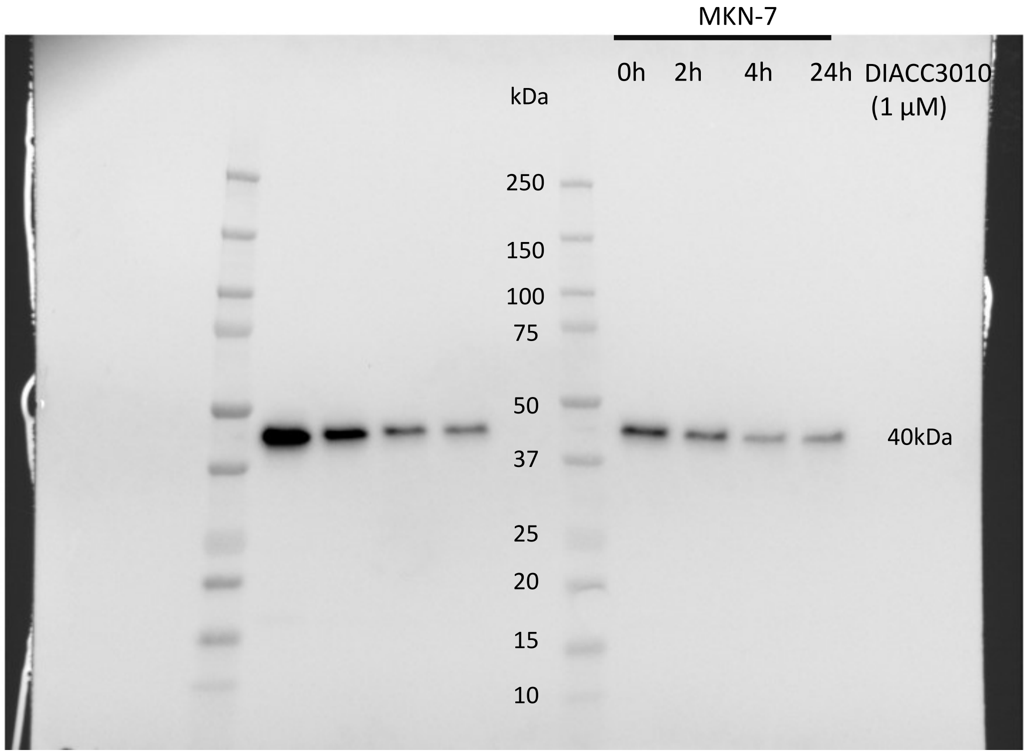

Original image
Figure 3
MKN-7

PRAS40

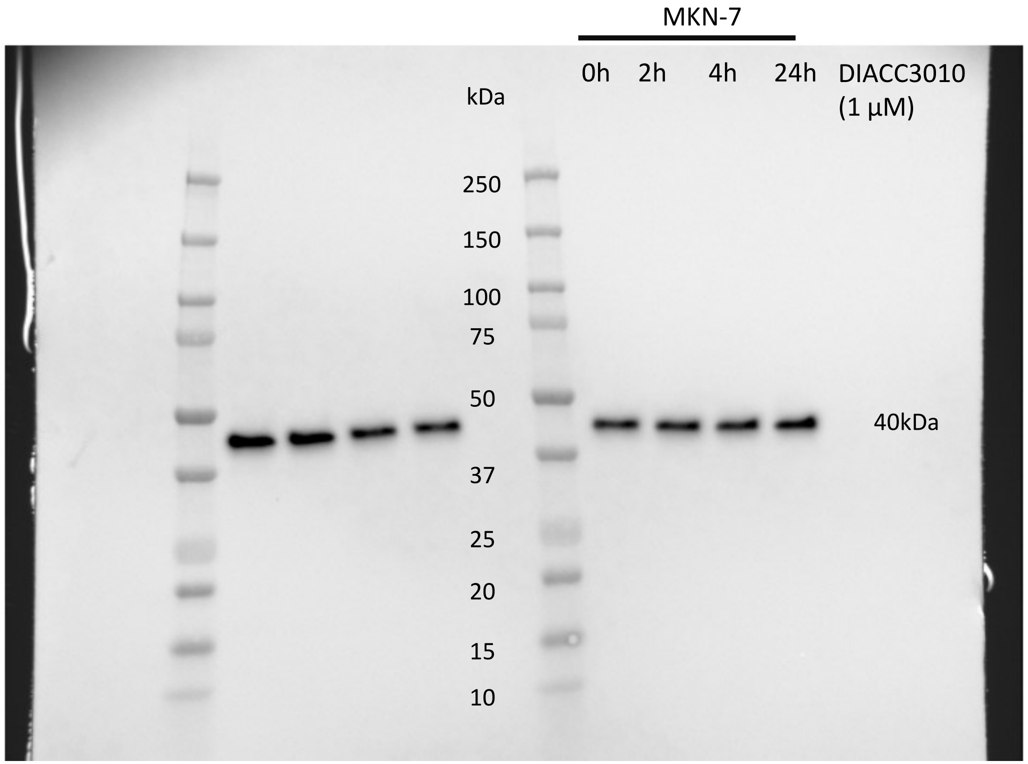

Original image
Figure 3
MKN-7

Phospho-S6 Ribosomal protein (Ser240/244)

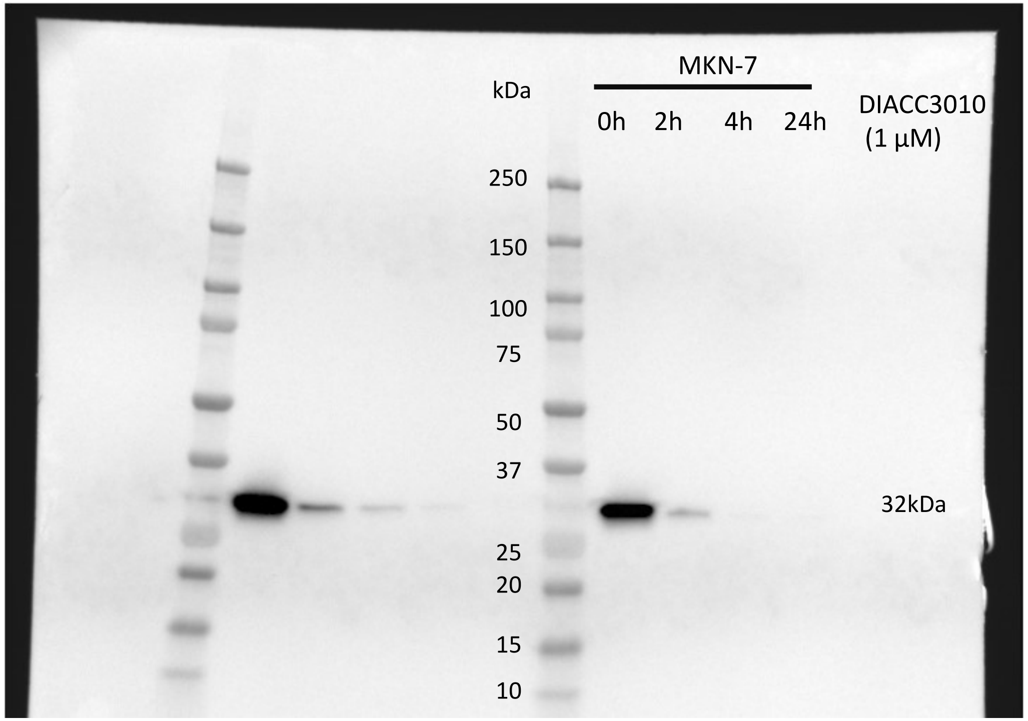

Original image
Figure 3
MKN-7

S6 Ribosomal protein

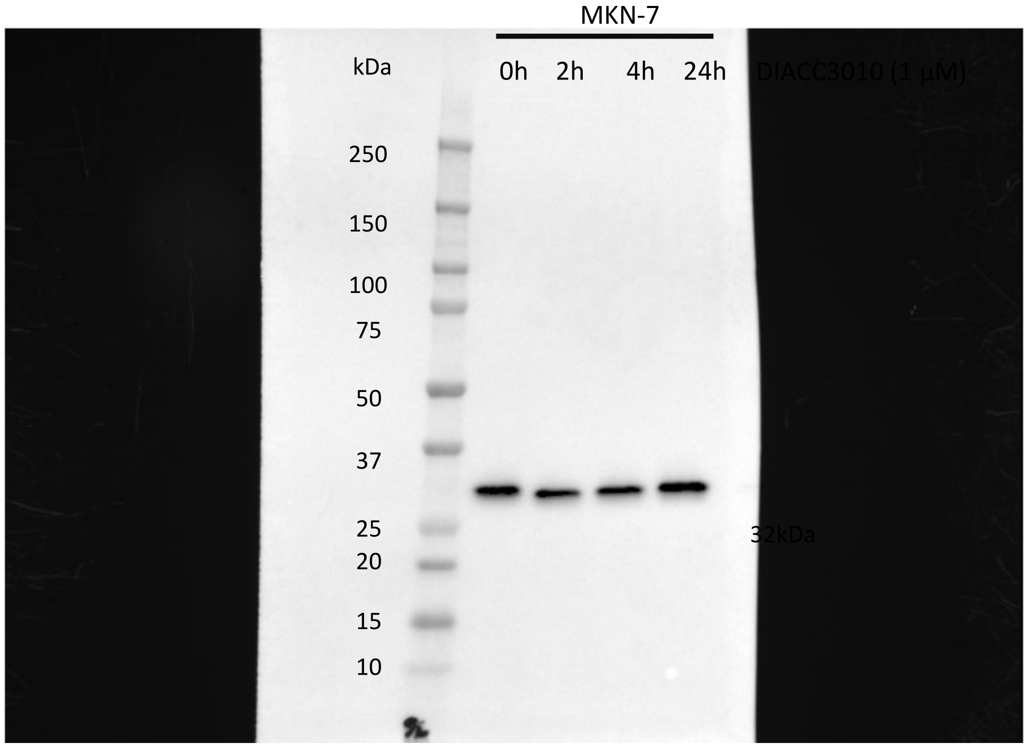

Original image
Figure 3
MKN-7

β-Actin

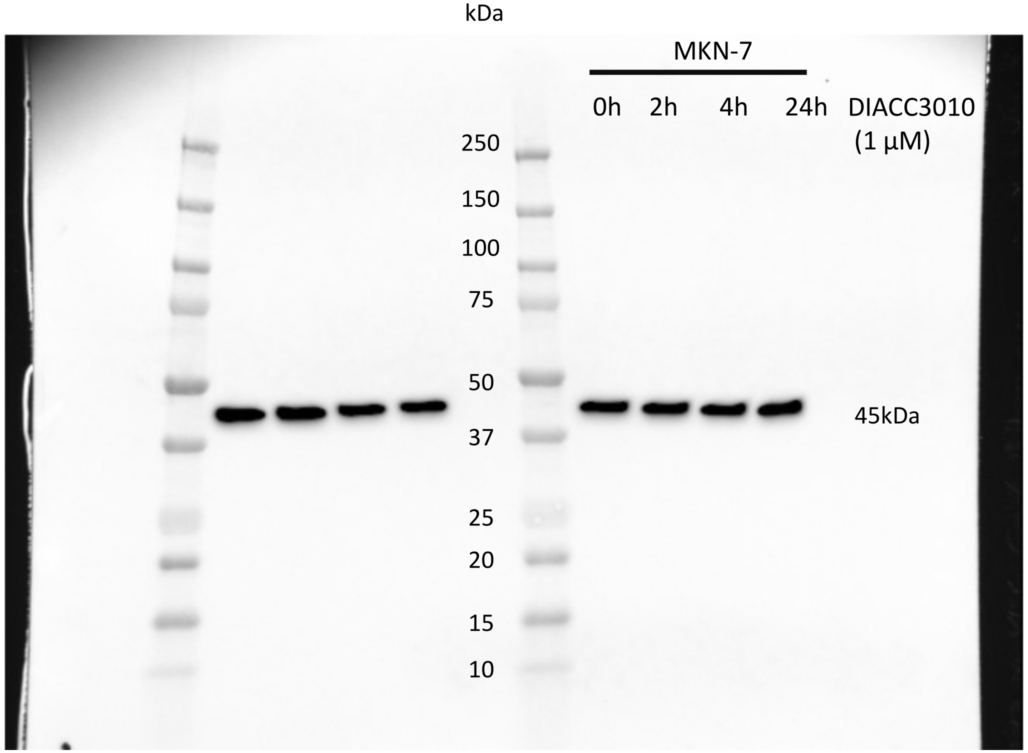

Original image
Figure 3
OE-19

Phospho-p44/42 MAPK (Erk1/2)(Thr202/Tyr204)

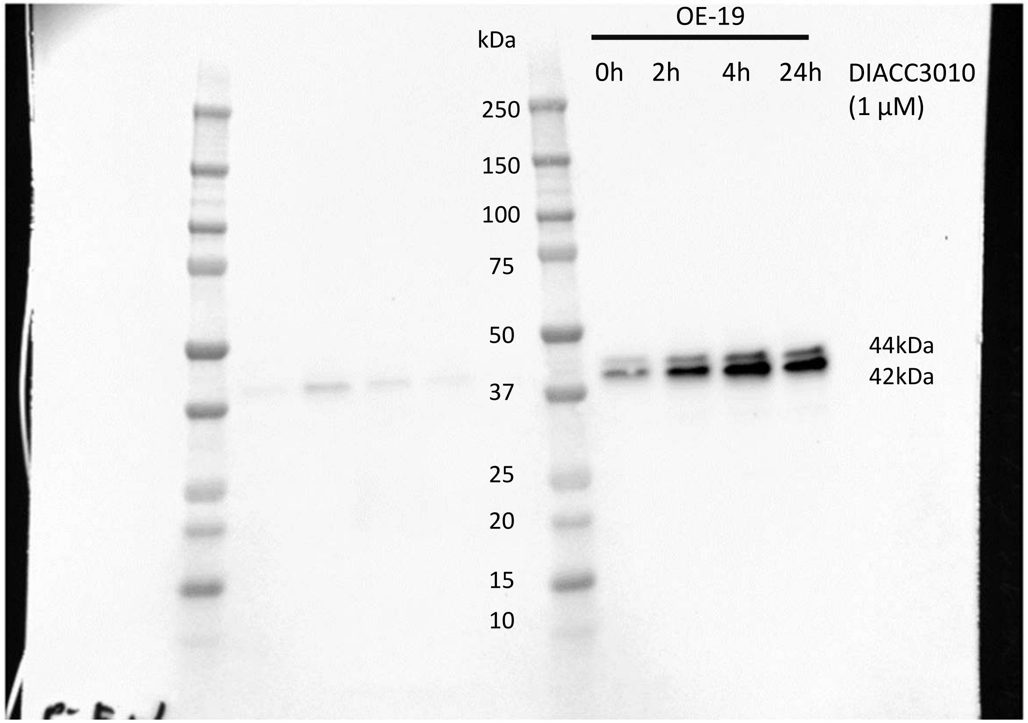

Original image
Figure 3
OE-19

P44/42 MAPK (Erk1/2)

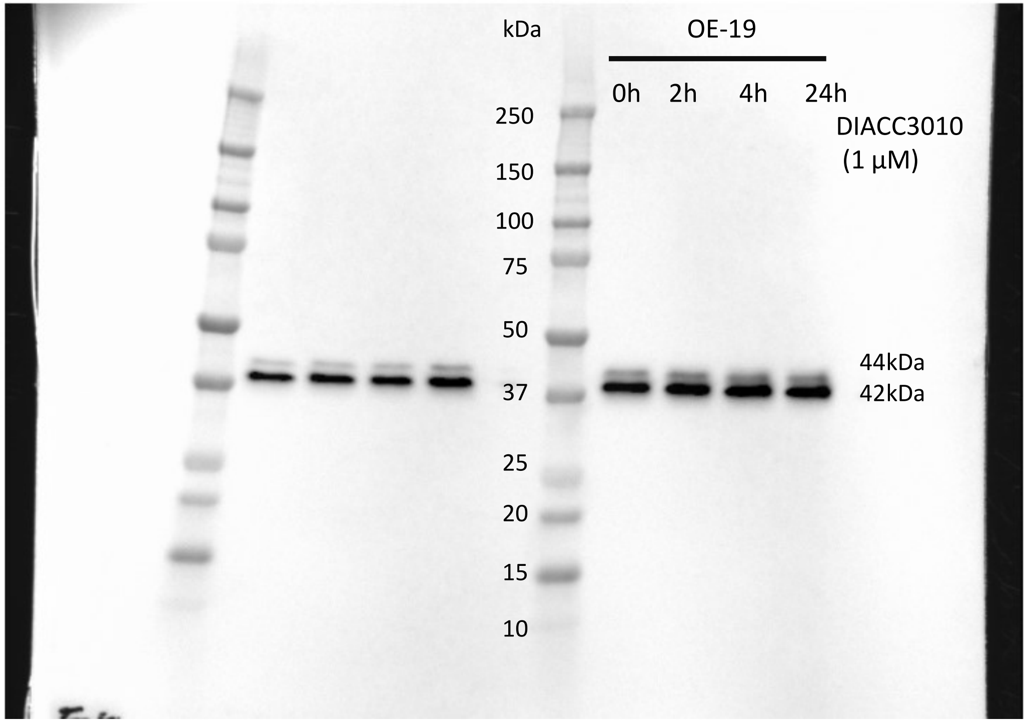

Original image
Figure 3
OE-19

Phospho-Akt (Ser473)

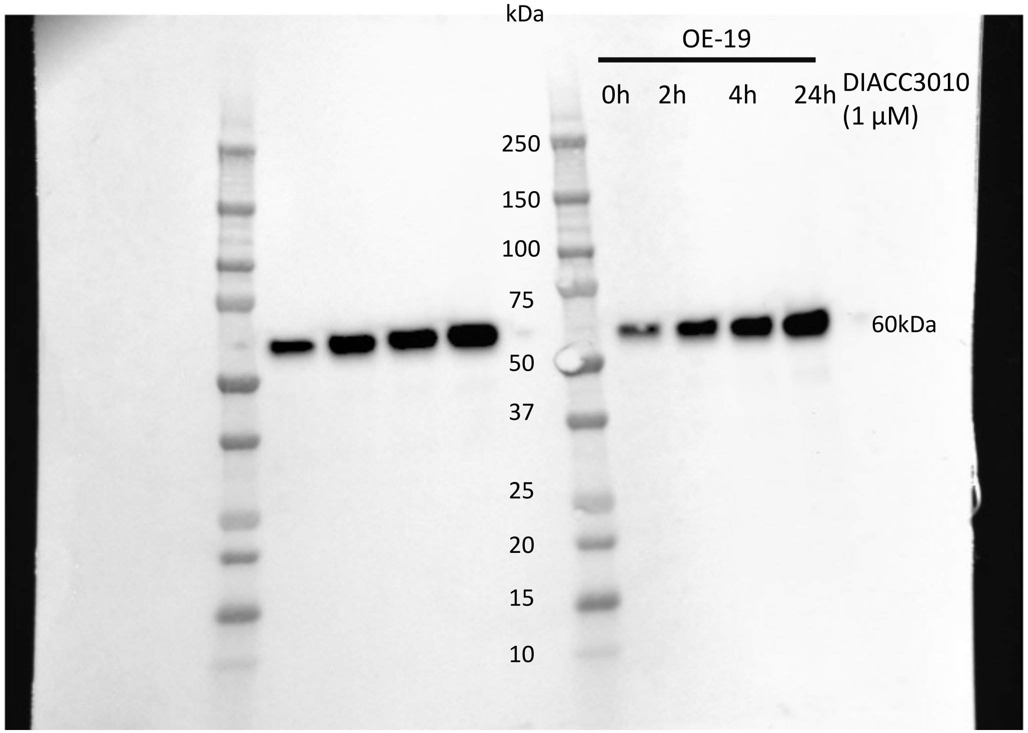

Original image
Figure 3
OE-19

Akt (pan)

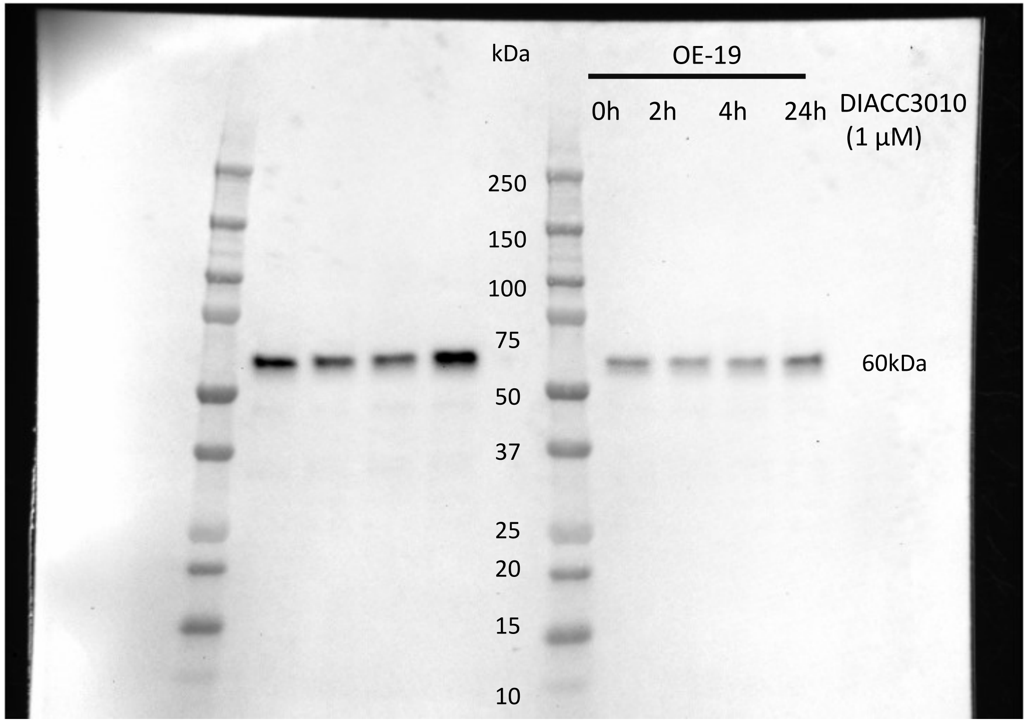

Original image
Figure 3
OE-19

Phospho-PRAS40 (Thr246)

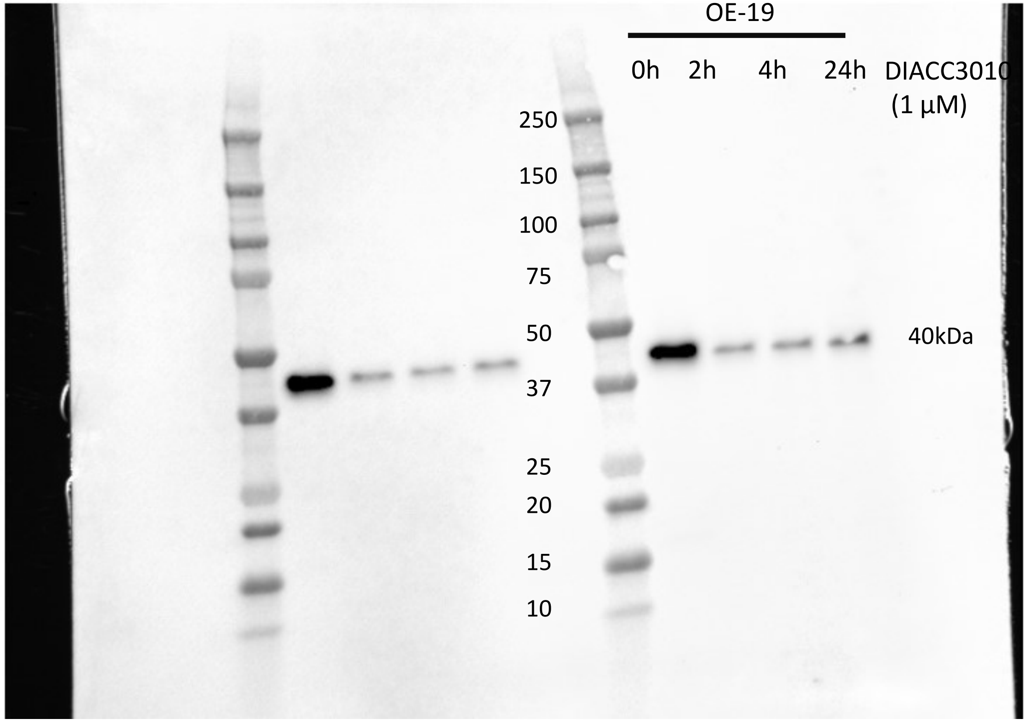

Original image
Figure 3
OE-19

PRAS40

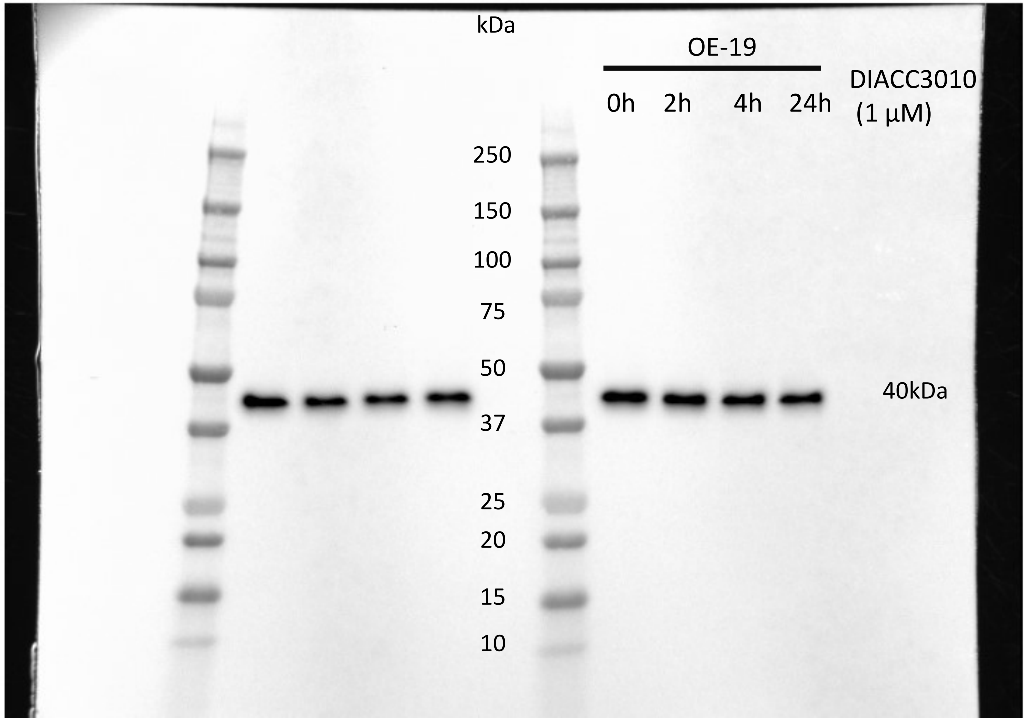

Original image
Figure 3
OE-19

Phospho-S6 Ribosomal protein (Ser240/244)

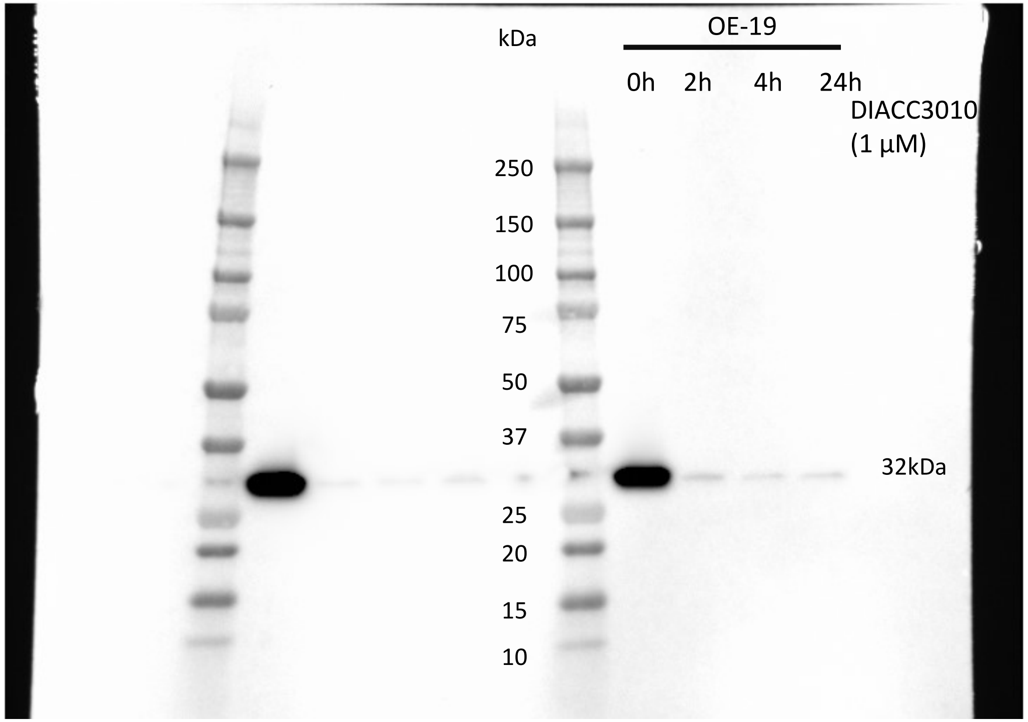

Original image
Figure 3
OE-19

S6 Ribosomal protein

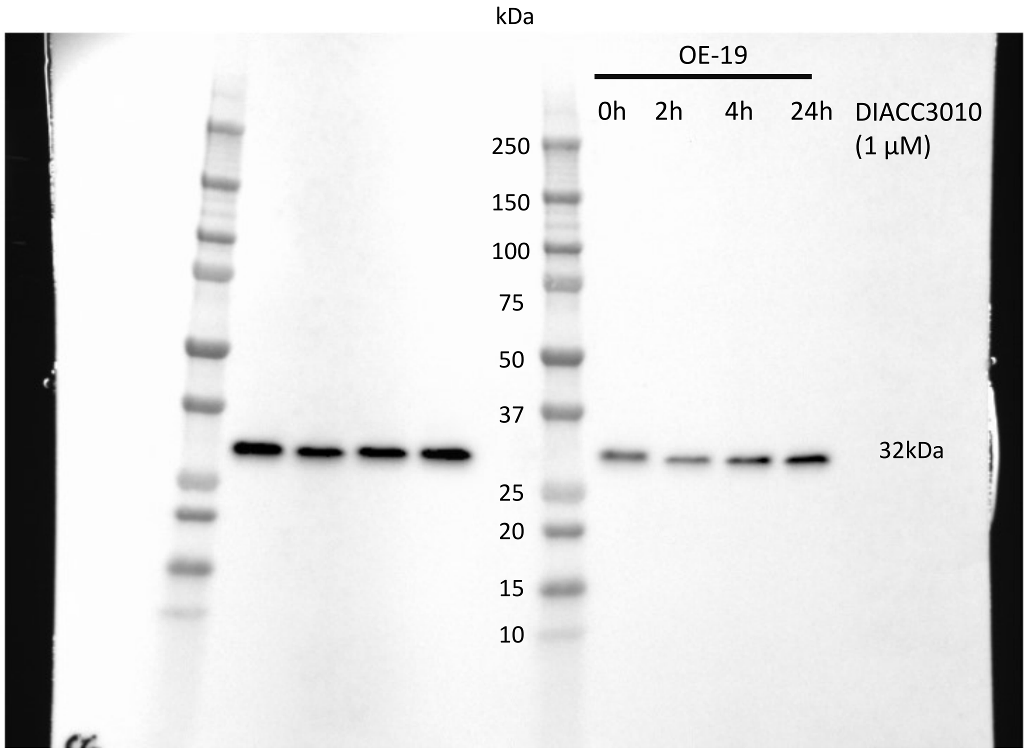

Original image
Figure 3
OE-19

β-Actin

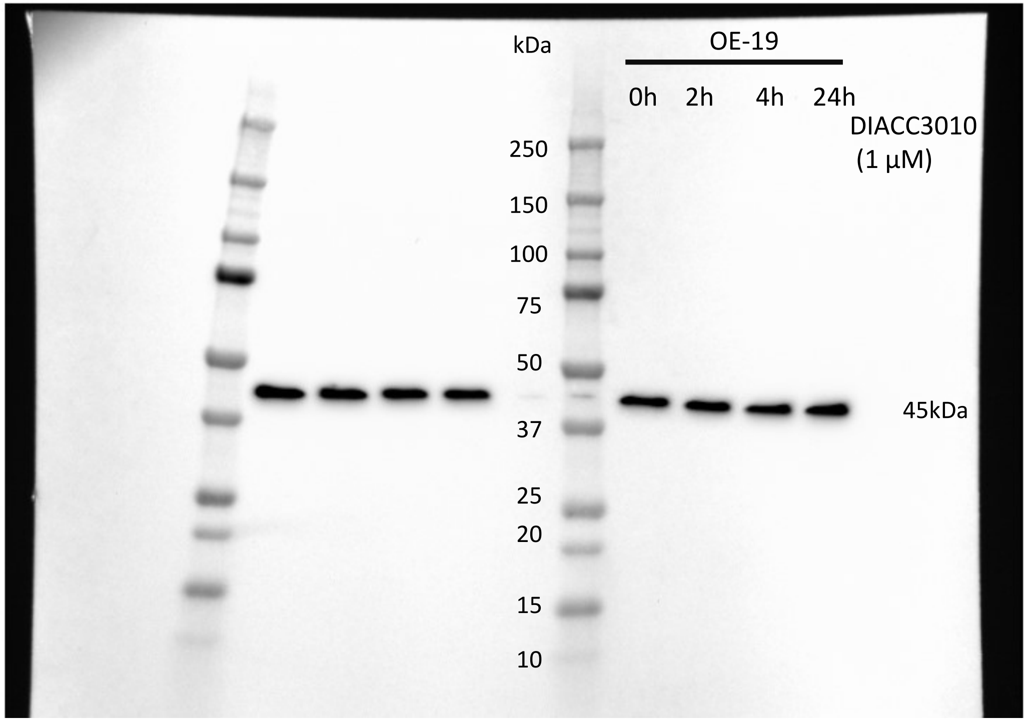

Original image
Figure 4

P44/42 MAPK (Erk1/2)

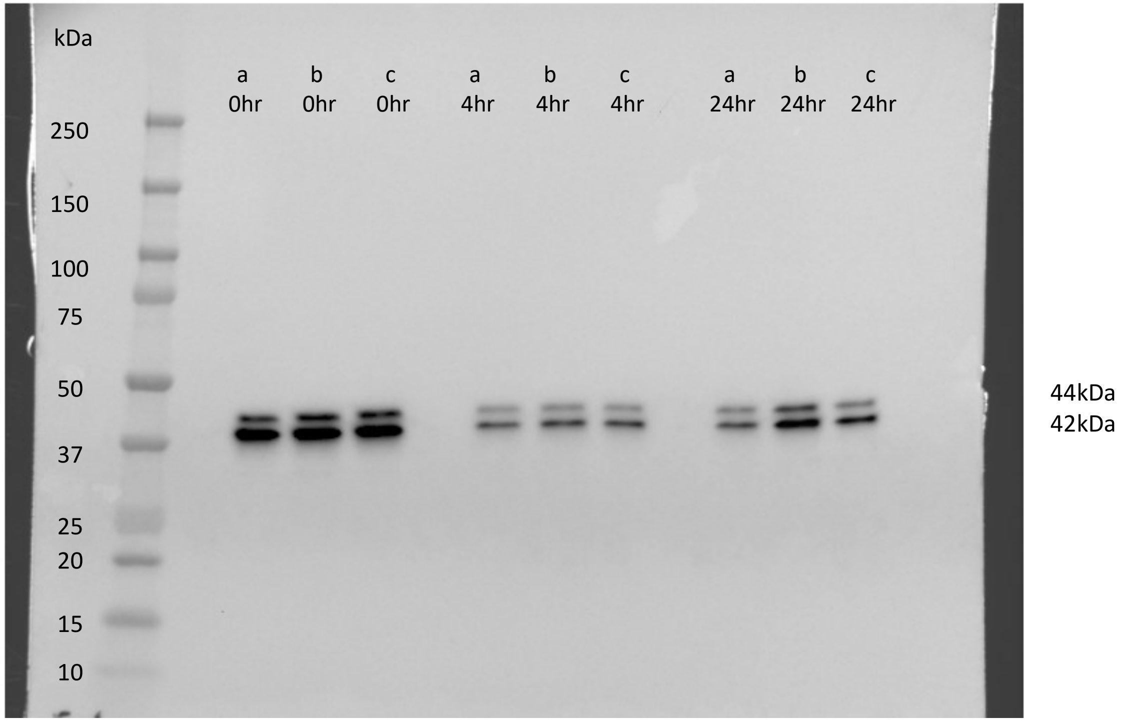

Original image
Figure 4

Phospho-p44/42 MAPK (Erk1/2)(Thr202/Tyr204)

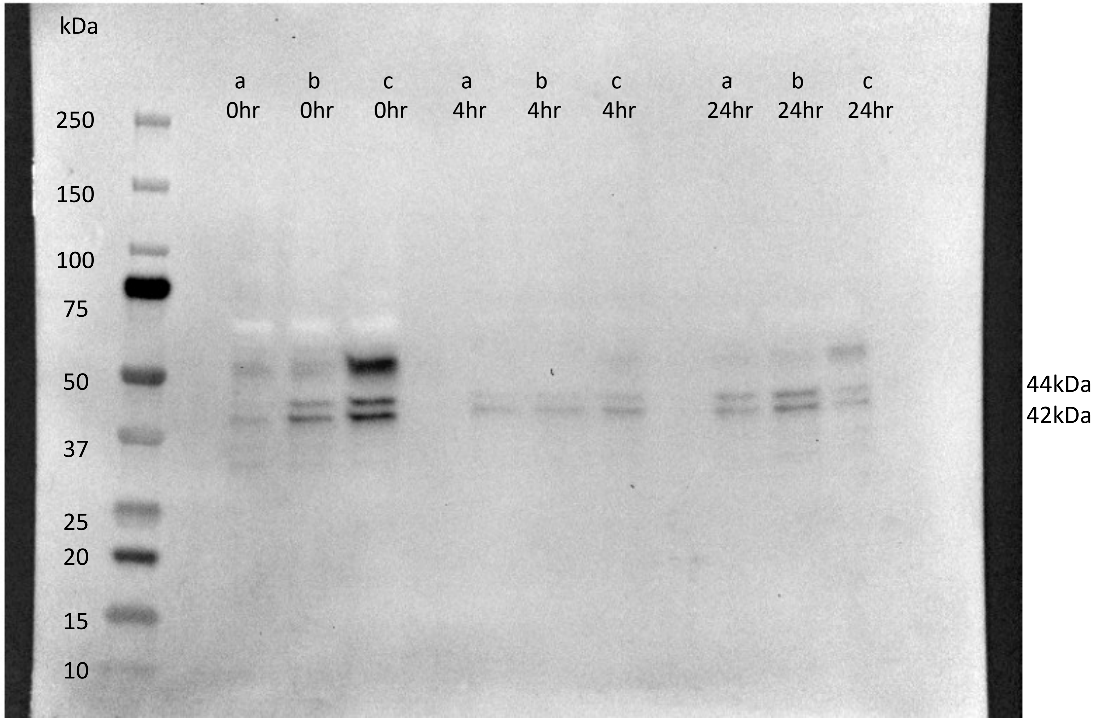

Original image
Figure 4

Akt (pan)

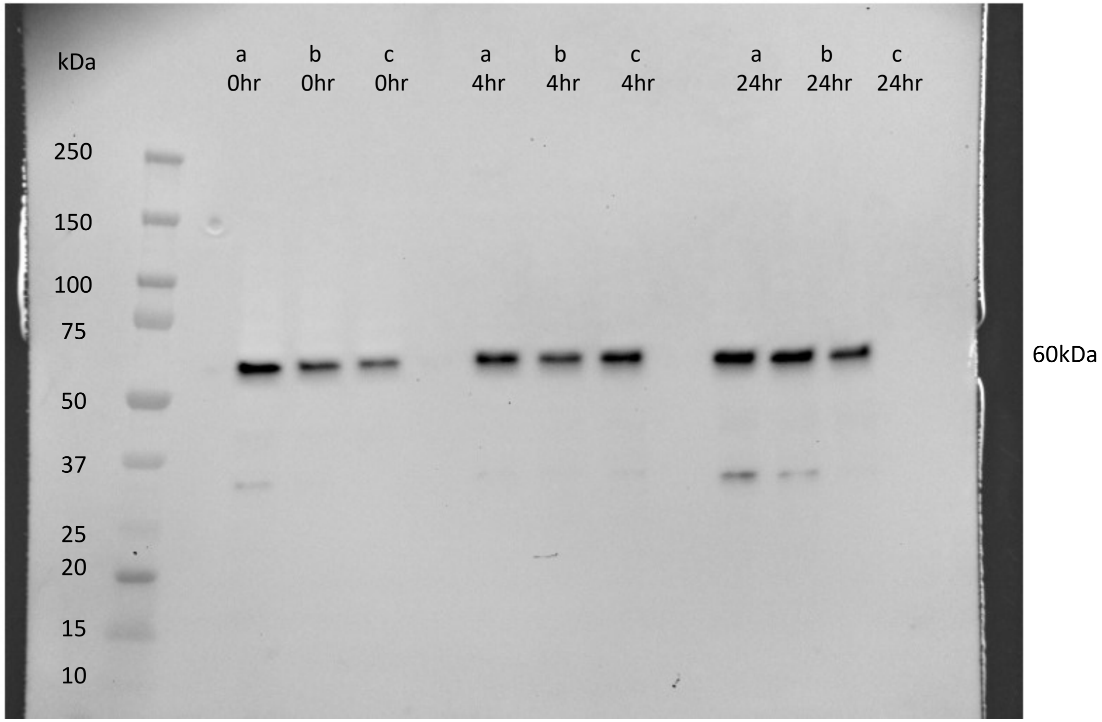

Original image
Figure 4

Phospho-Akt (Ser473)

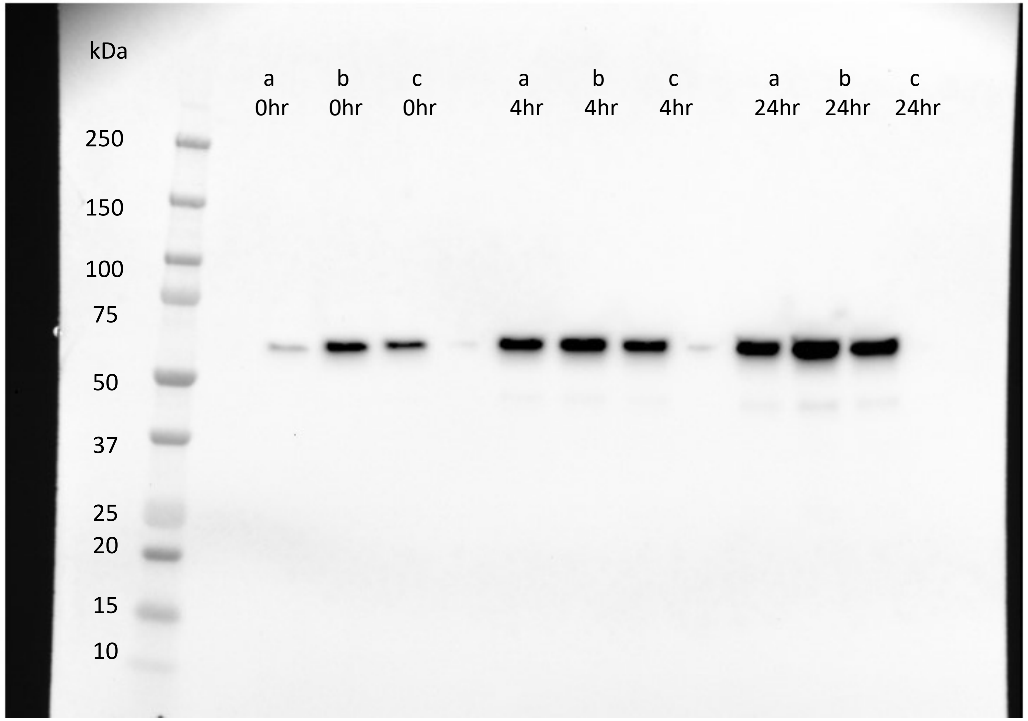

60kDa

Original image
Figure 4

PRAS40

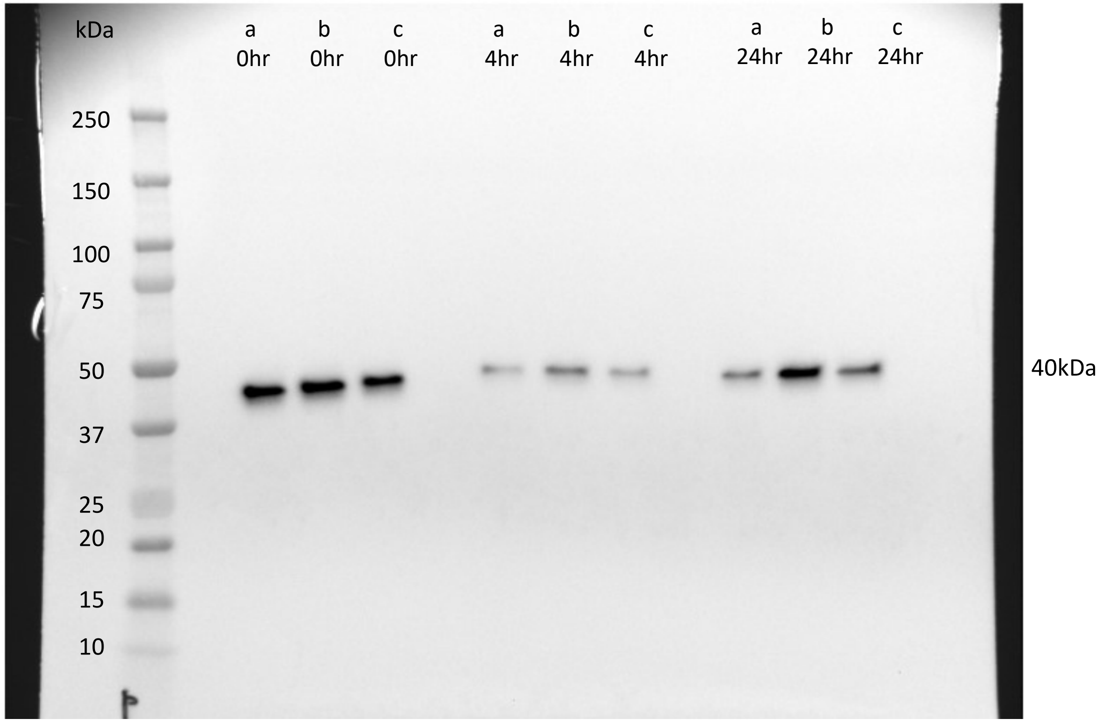

Original image
Figure 4

Phospho-PRAS40 (Thr246)

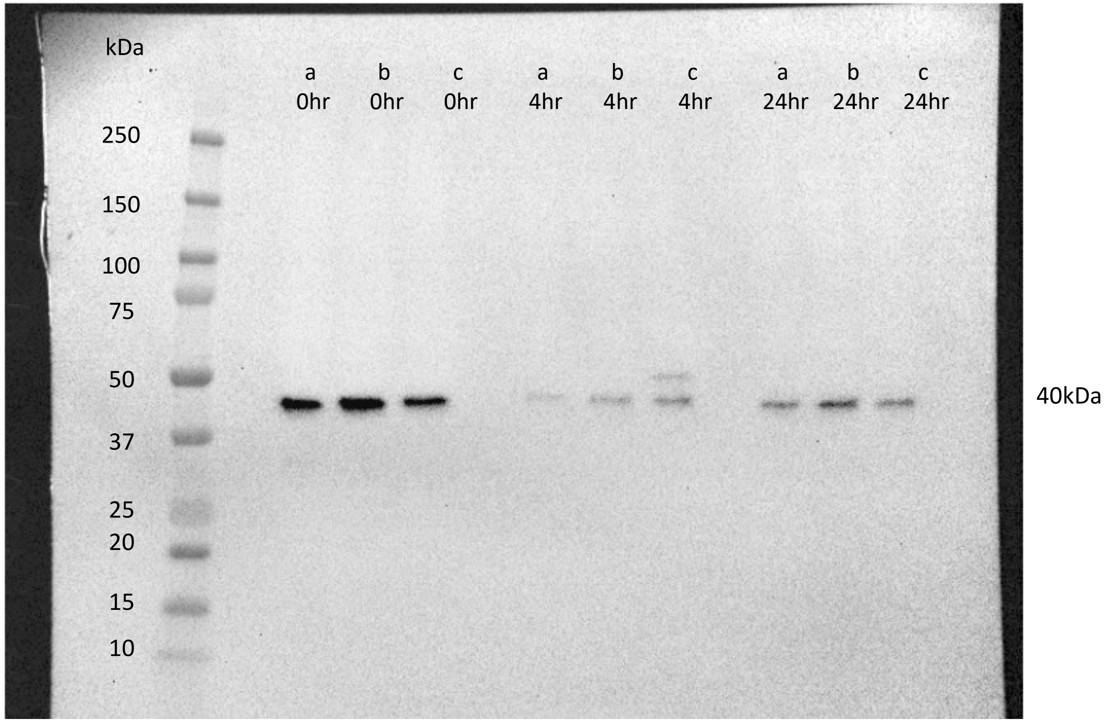

Original image
Figure 4

S6 Ribosomal protein

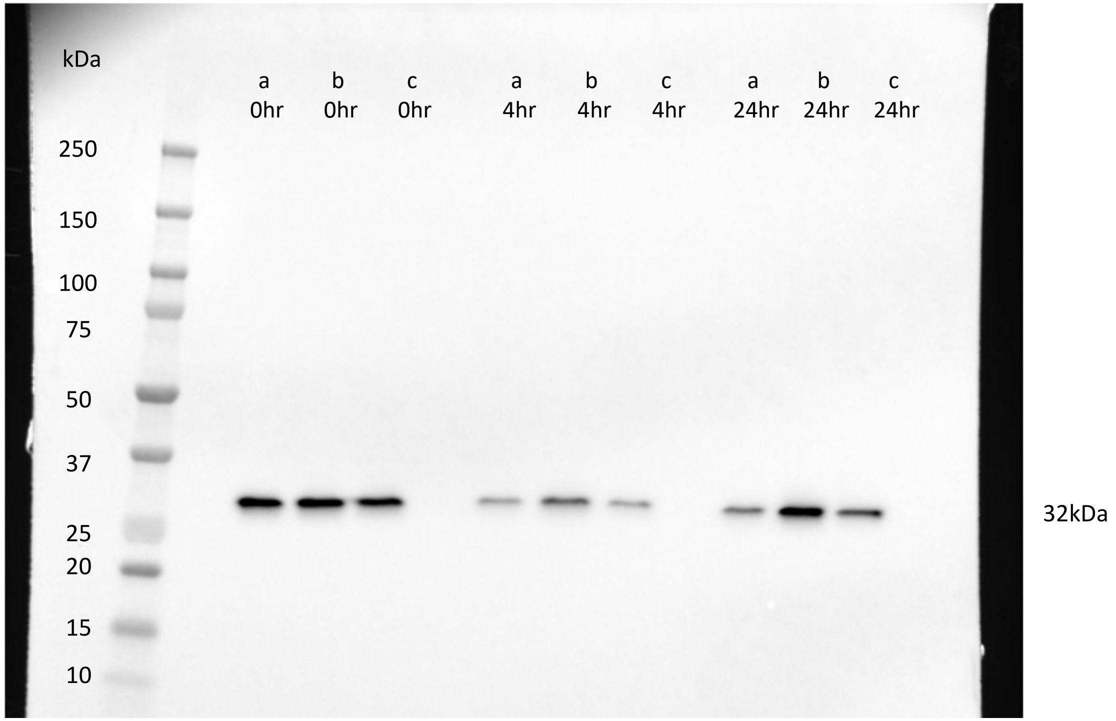

Original image
Figure 4

Phospho-S6 Ribosomal protein (Ser240/244)

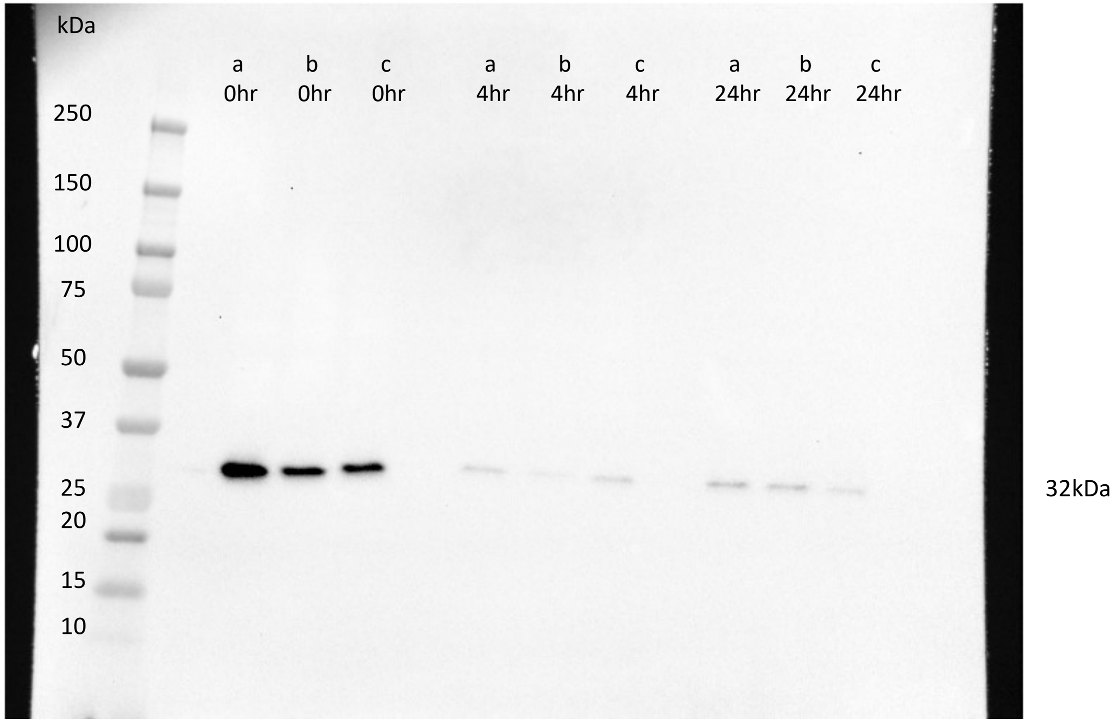

Original image
Figure 4

β-Actin

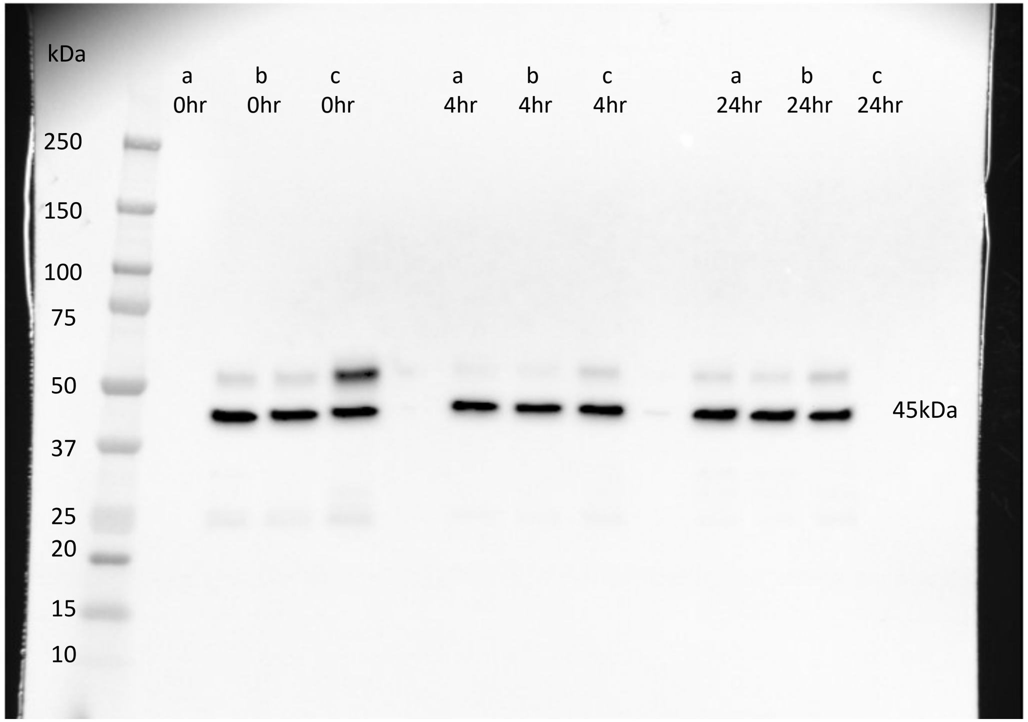

Original image
Figure 6
OE-19

Phospho-p44/42 MAPK (Erk1/2)(Thr202/Tyr204)

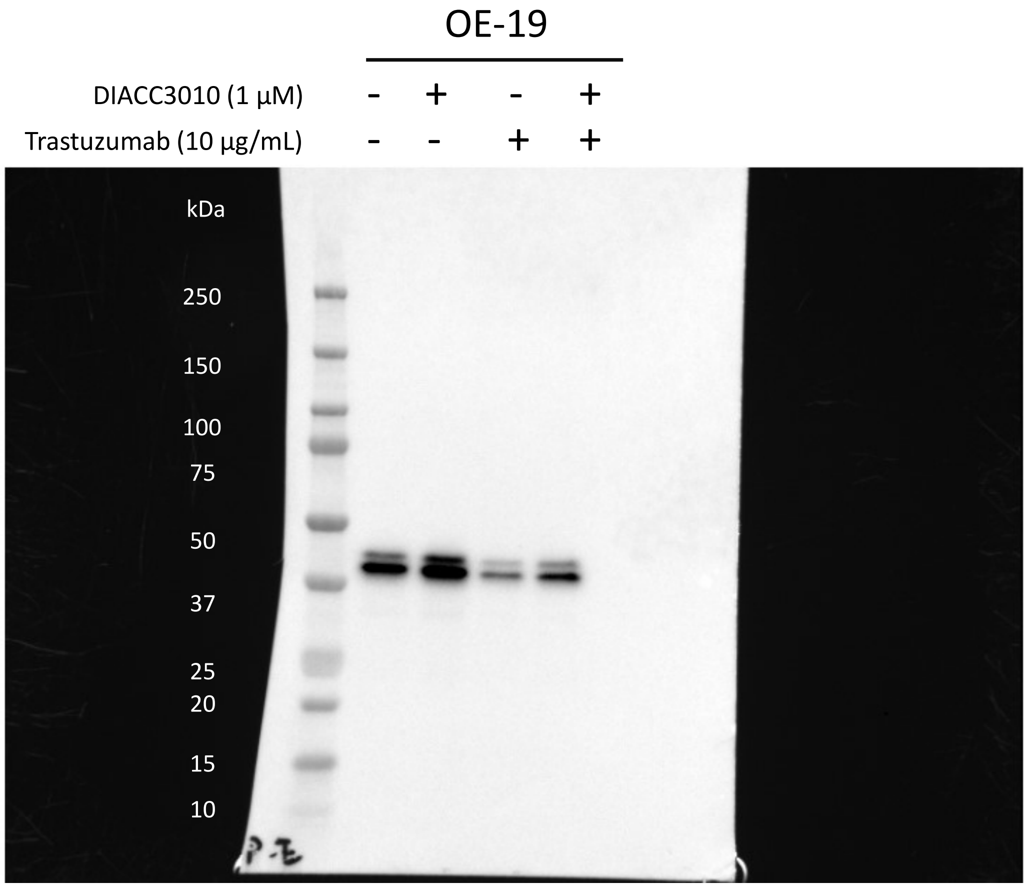

Original image
Figure 6
OE-19

P44/42 MAPK (Erk1/2)

	OE-19			
DIACC3010 (1 µM)	-	+	-	+
Trastuzumab (10 µg/mL)	-	-	+	+

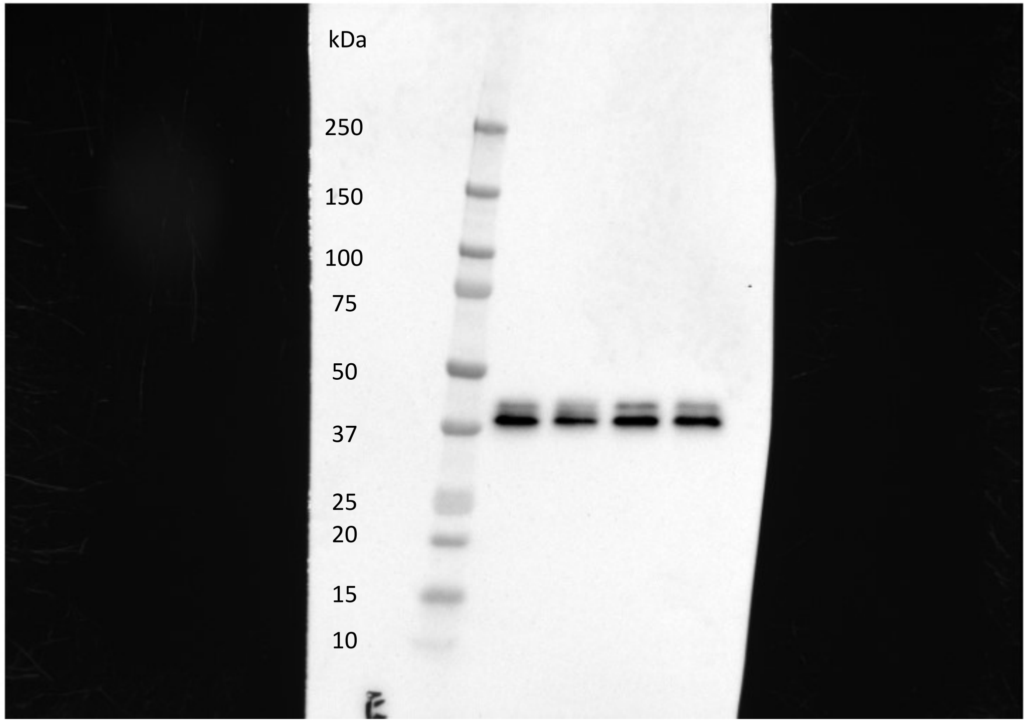

Original image
Figure 6
OE-19

Phospho-Akt (Ser473)

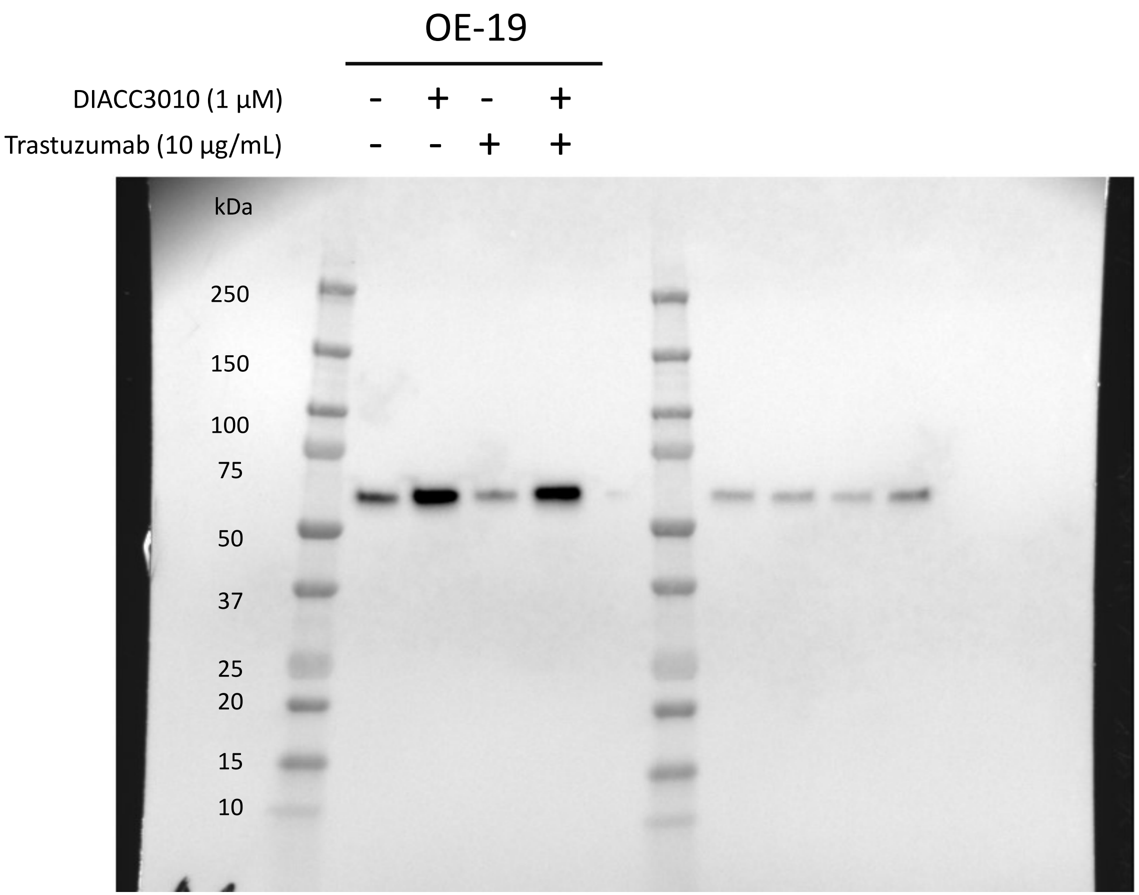

Original image
Figure 6
OE-19

Akt (pan)

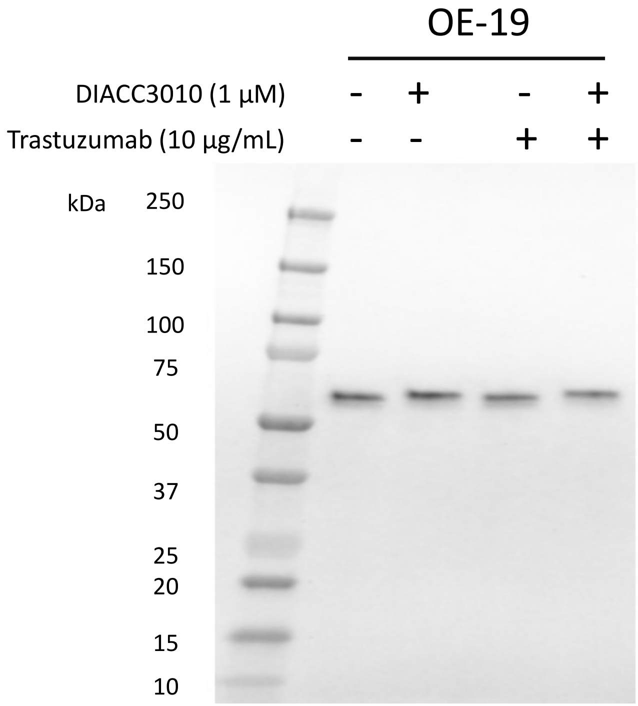

Original image
Figure 6
OE-19

Phospho-PRAS40 (Thr246)

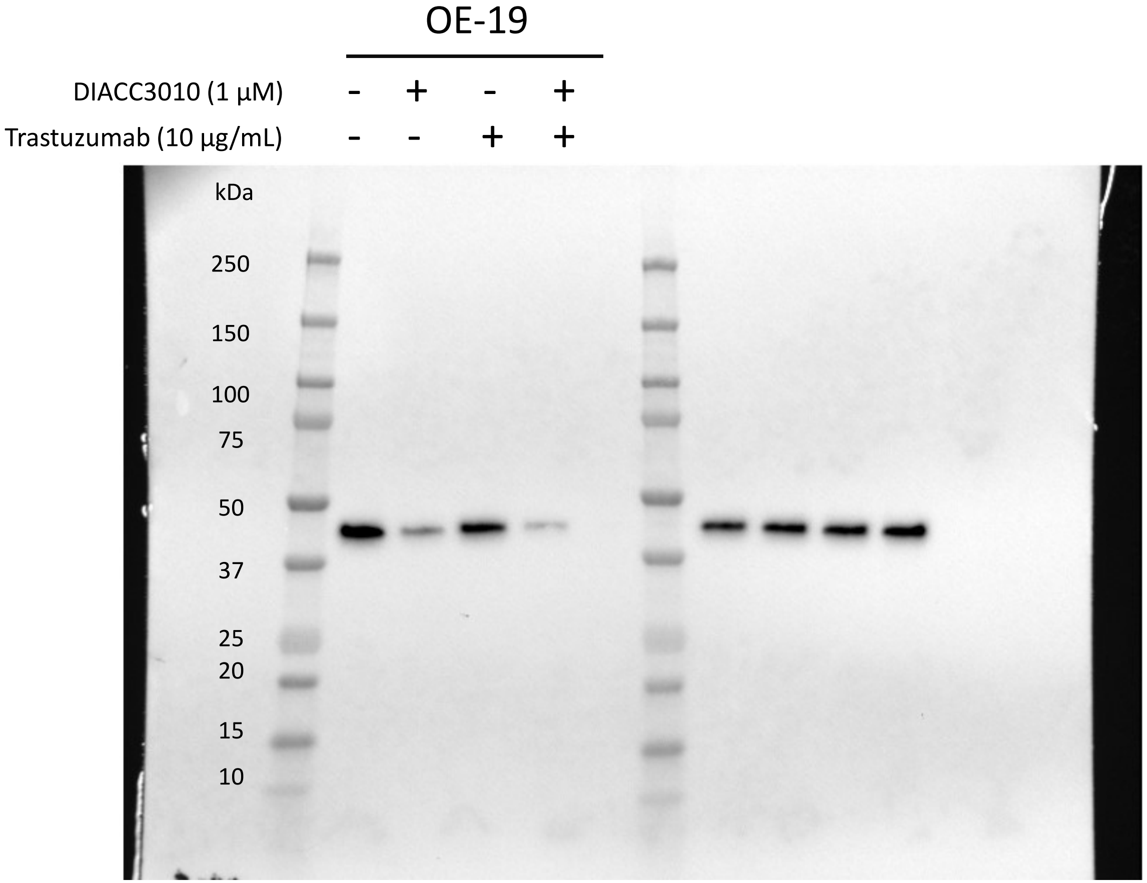

Original image
Figure 6
OE-19

PRAS40

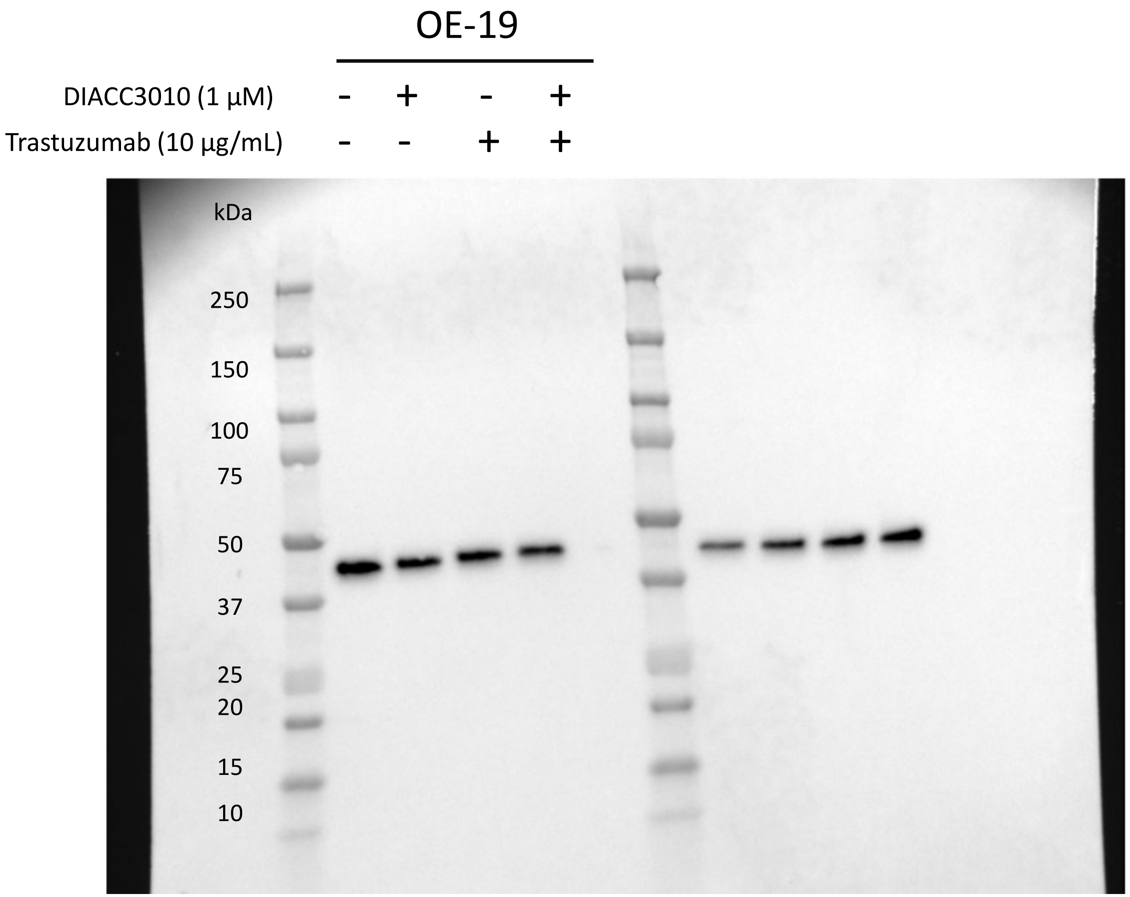

Original image
Figure 6
OE-19

Phospho-S6 Ribosomal protein (Ser240/244)

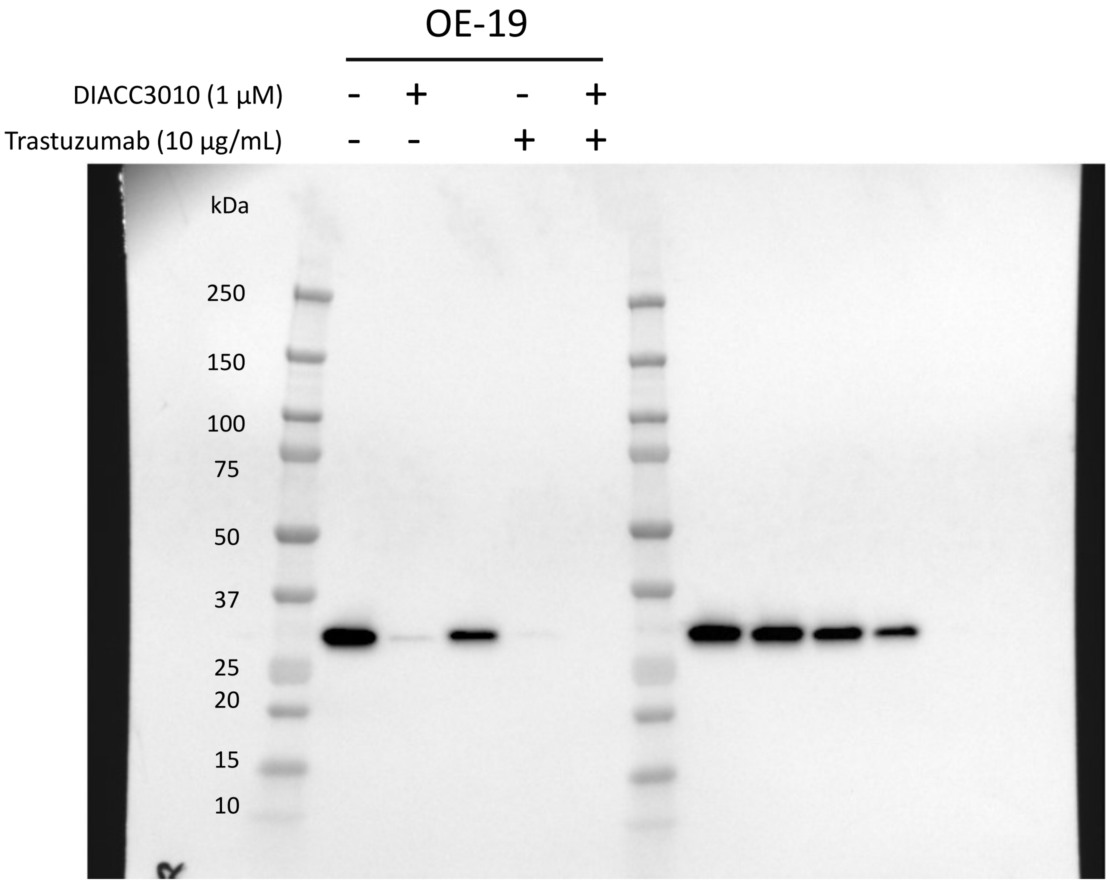

Original image
Figure 6
OE-19

S6 Ribosomal protein

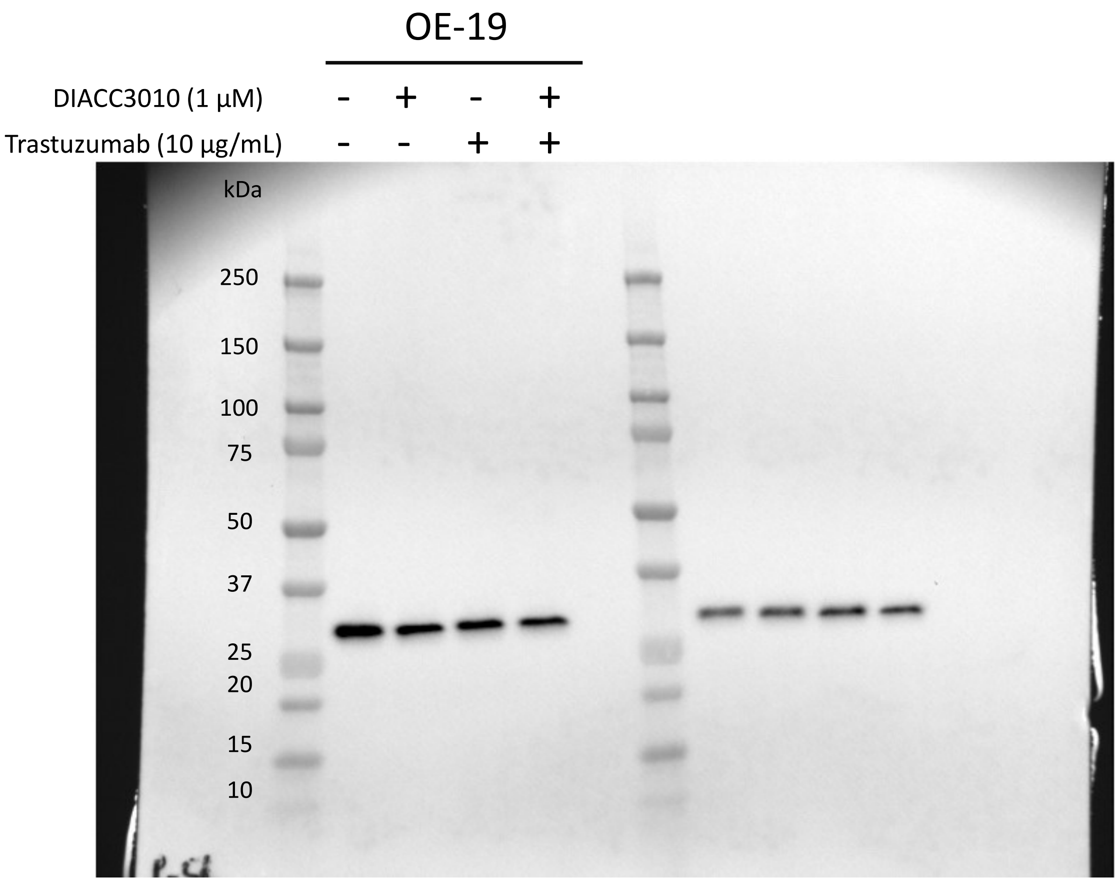

Original image
Figure 6
OE-19

β-Actin

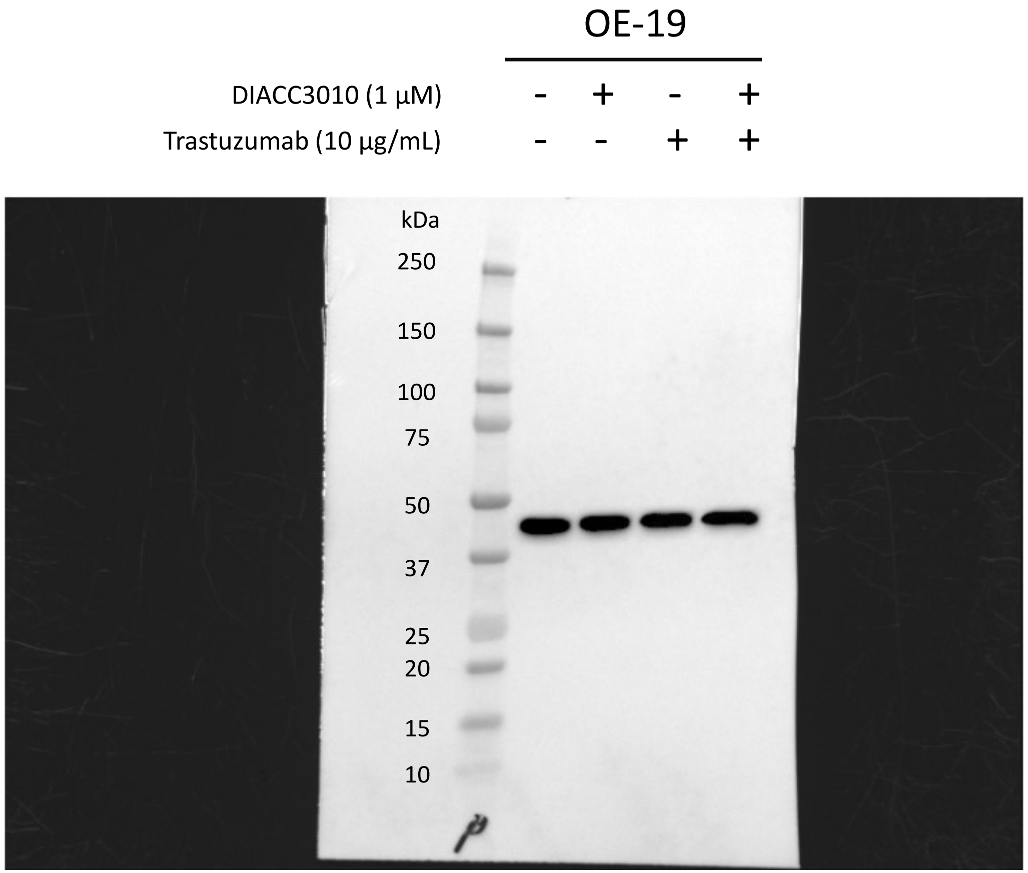

Original image
Figure 6
NCI-N87

Phospho-p44/42 MAPK (Erk1/2)(Thr202/Tyr204)

NCI-N87

DIACC3010 (1 µM)	-	+	-	+
Trastuzumab (10 µg/mL)	-	-	+	+

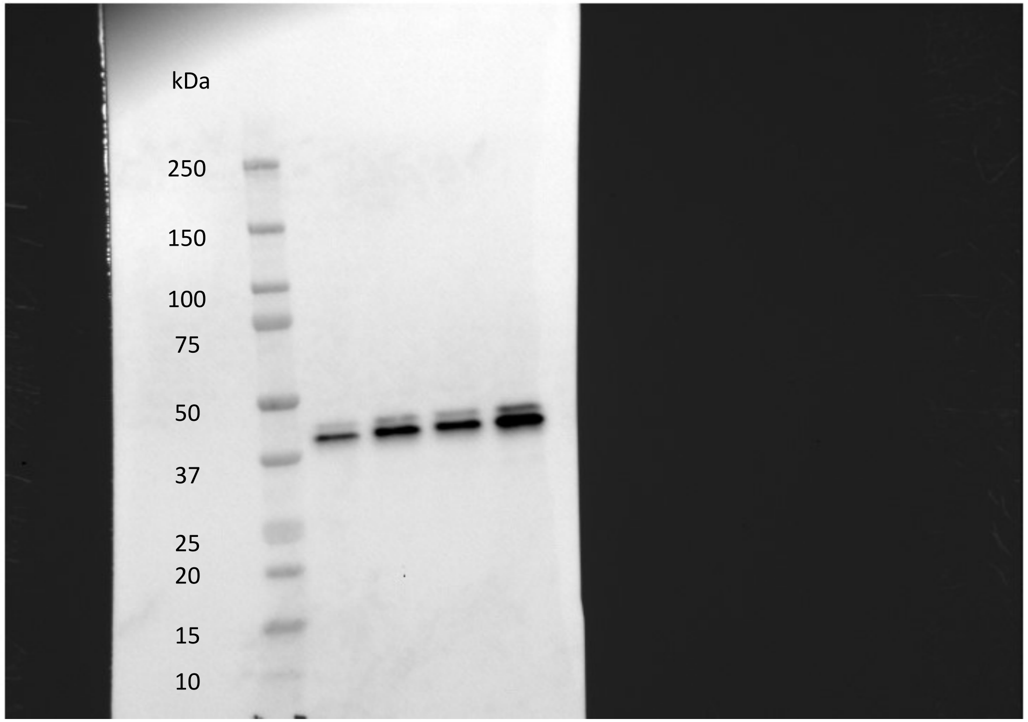

Original image
Figure 6
NCI-N87

P44/42 MAPK (Erk1/2)

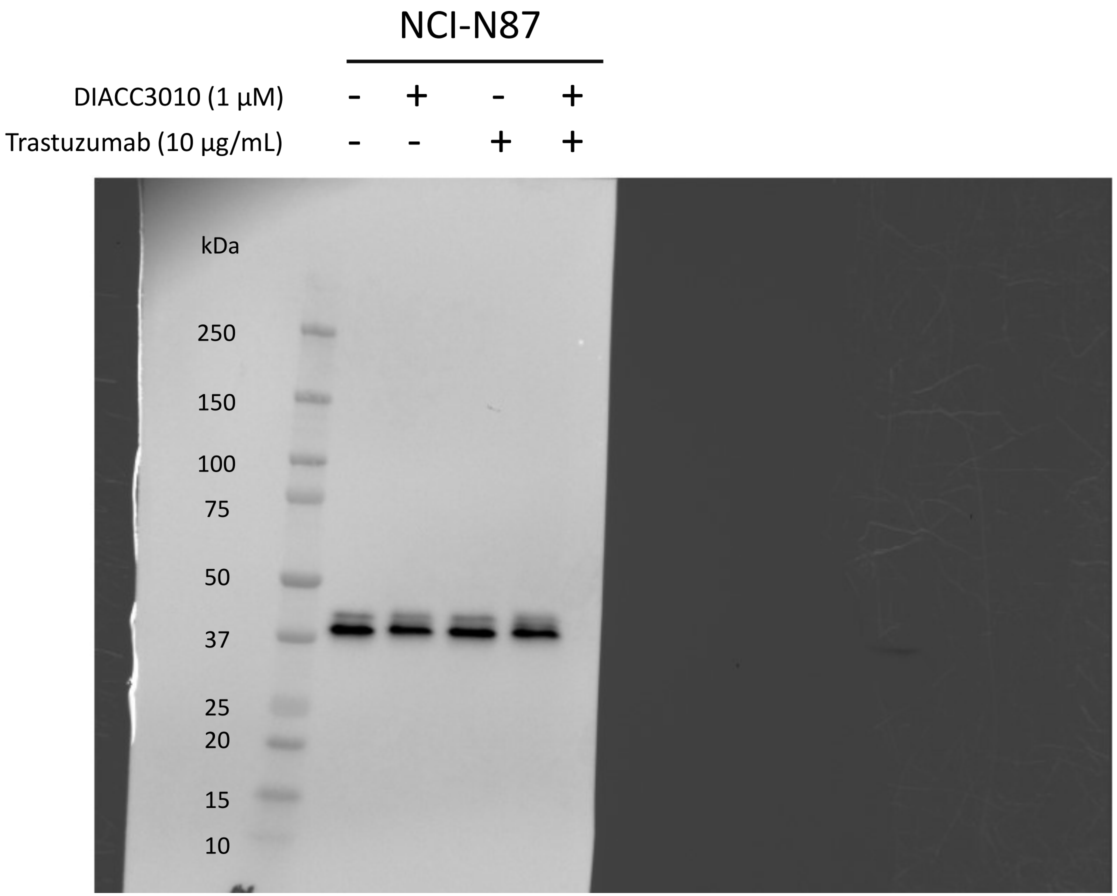

Original image
Figure 6
NCI-N87

Phospho-Akt (Ser473)

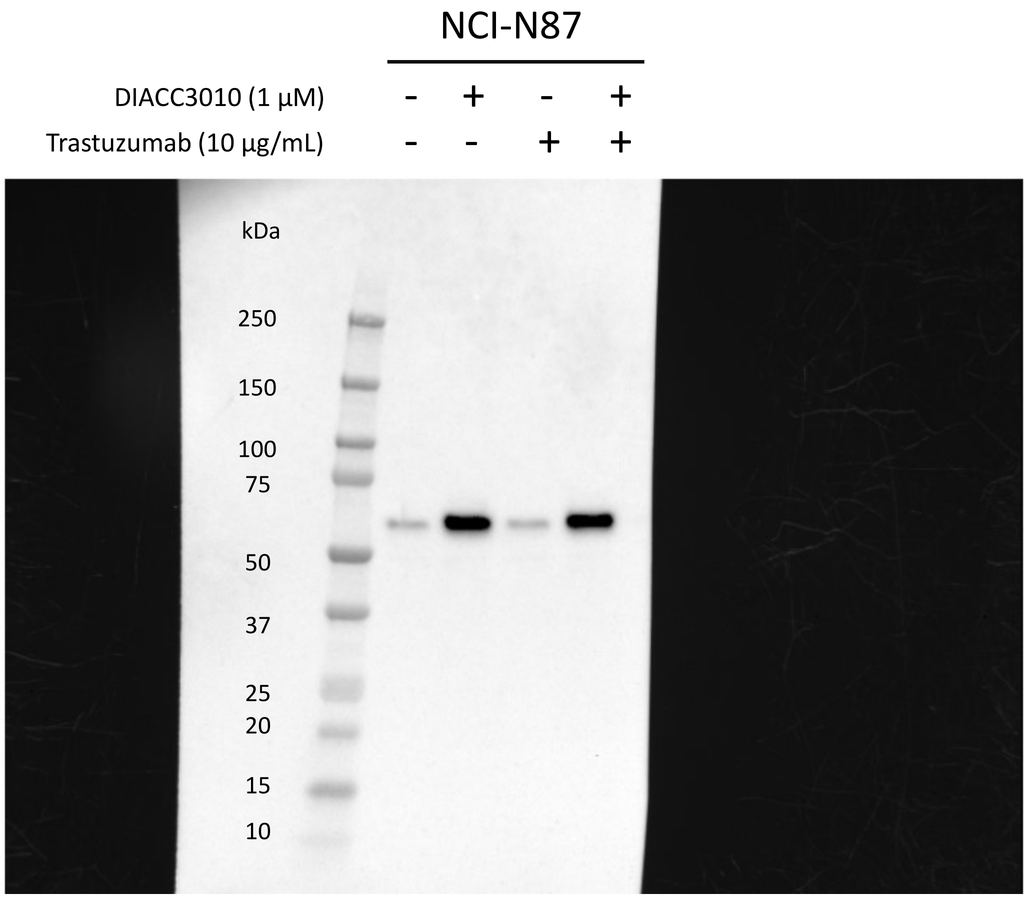

Original image
Figure 6
NCI-N87

Akt (pan)

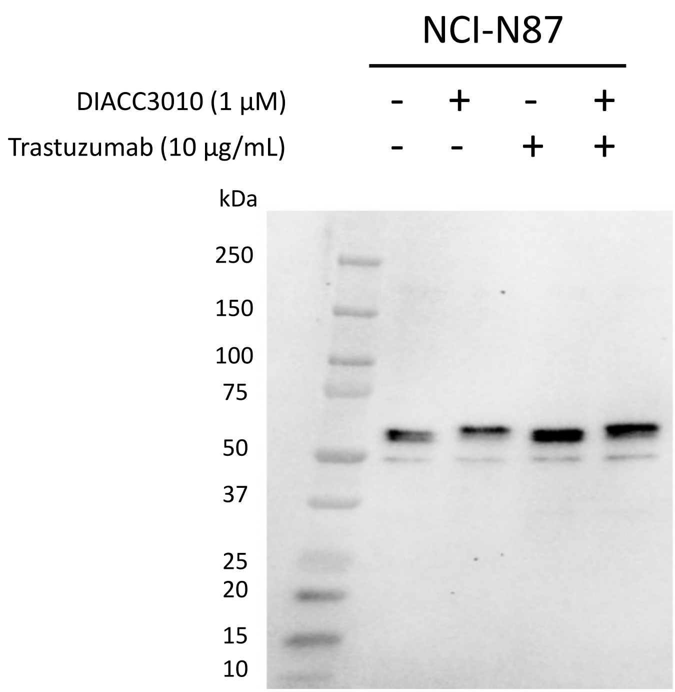

Original image
Figure 6
NCI-N87

Phospho-PRAS40 (Thr246)

NCI-N87

DIACC3010 (1 µM)	-	+	-	+
Trastuzumab (10 µg/mL)	-	-	+	+

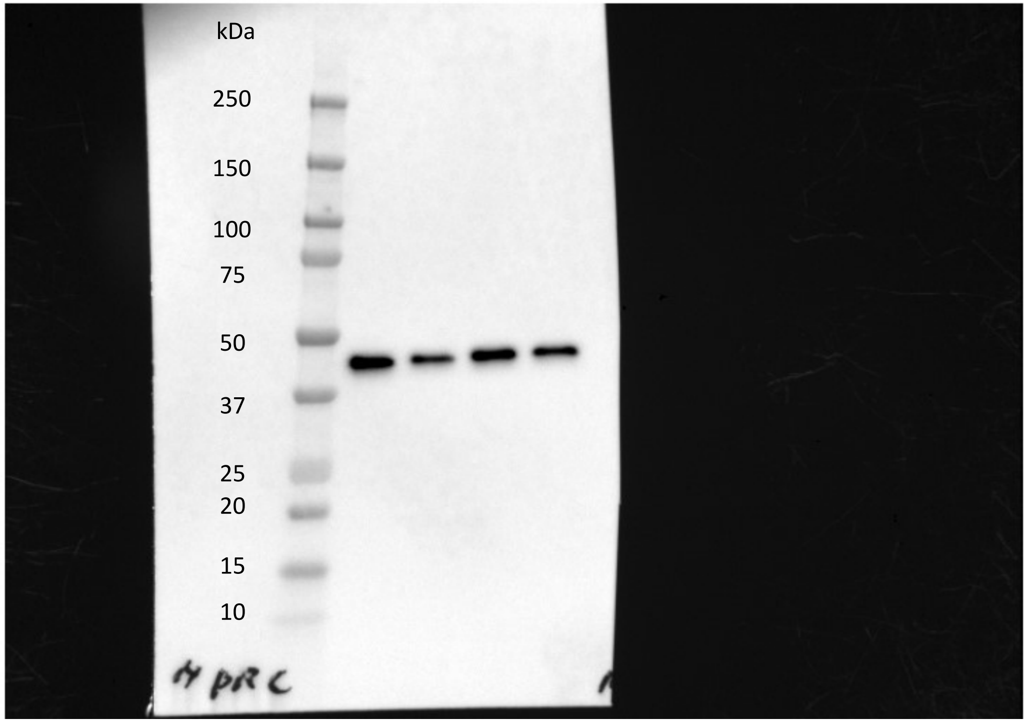

Original image
Figure 6
NCI-N87

PRAS40

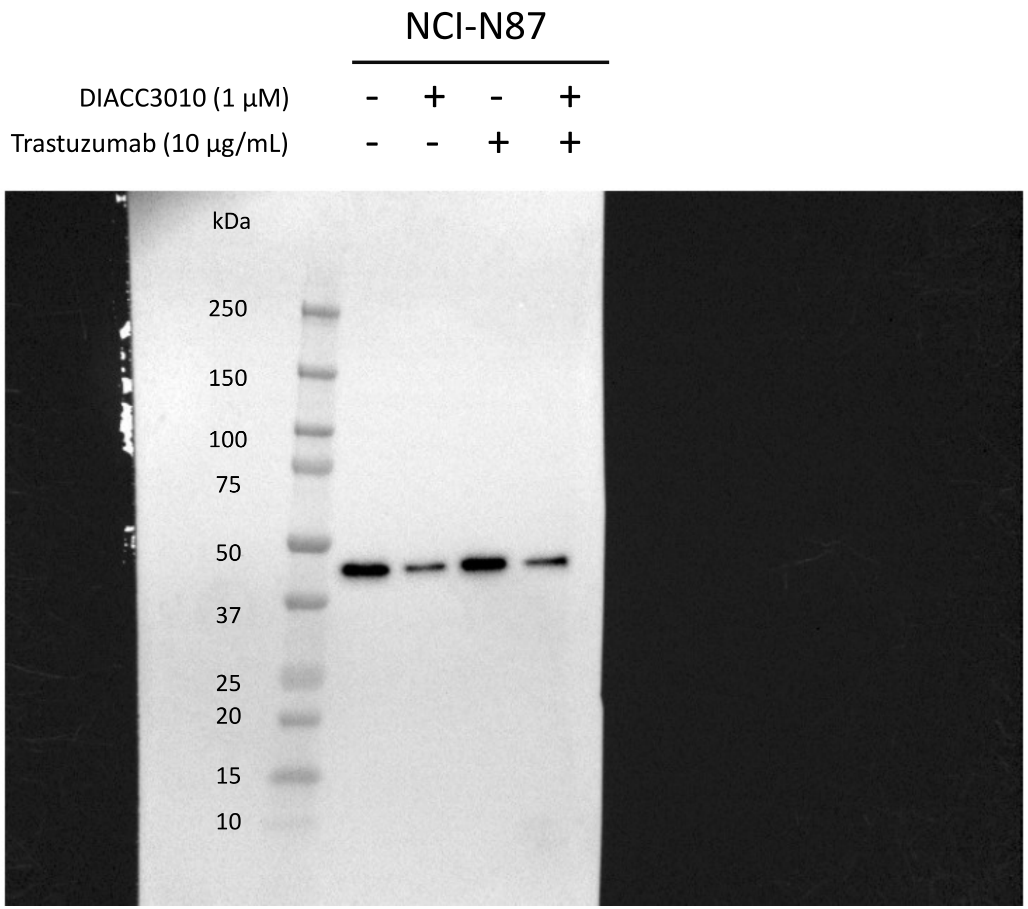

Original image
Figure 6
NCI-N87

Phospho-S6 Ribosomal protein (Ser240/244)

NCI-N87

DIACC3010 (1 µM)	-	+	-	+
Trastuzumab (10 µg/mL)	-	-	+	+

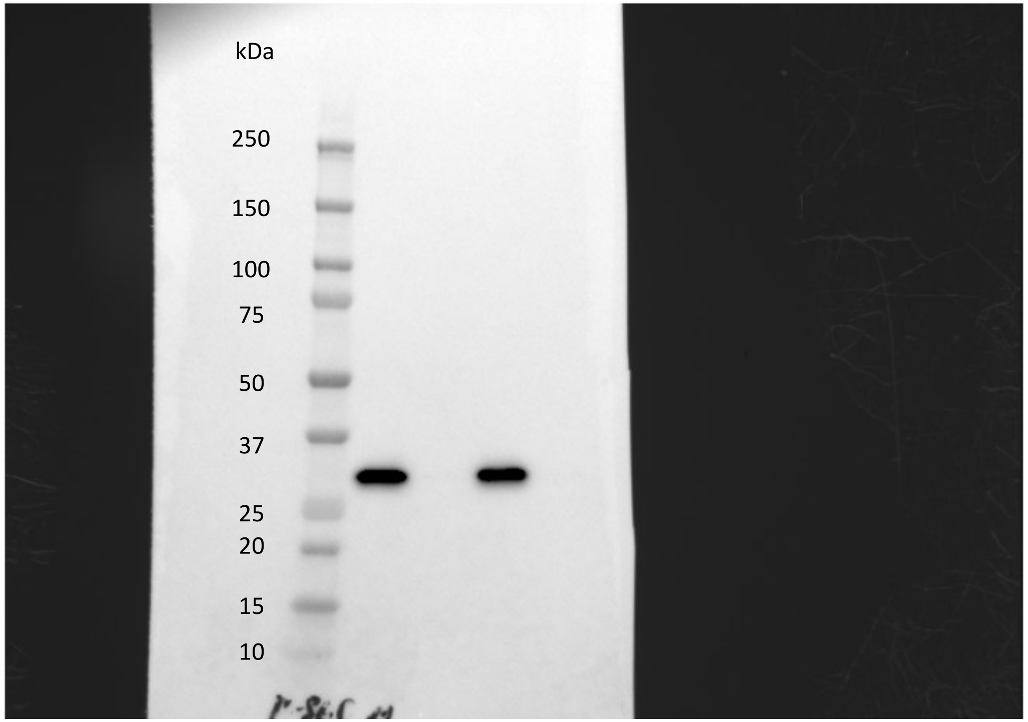

Original image
Figure 6
NCI-N87

S6 Ribosomal protein

NCI-N87

DIACC3010 (1 µM)	-	+	-	+
Trastuzumab (10 µg/mL)	-	-	+	+

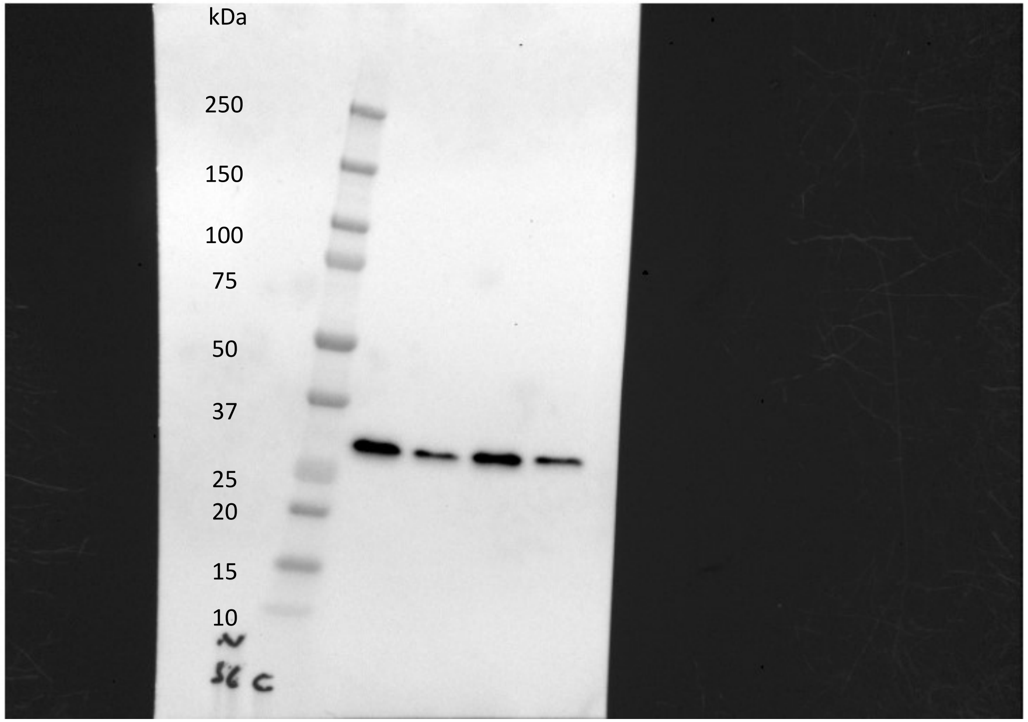

Original image
Figure 6
NCI-N87

β-Actin

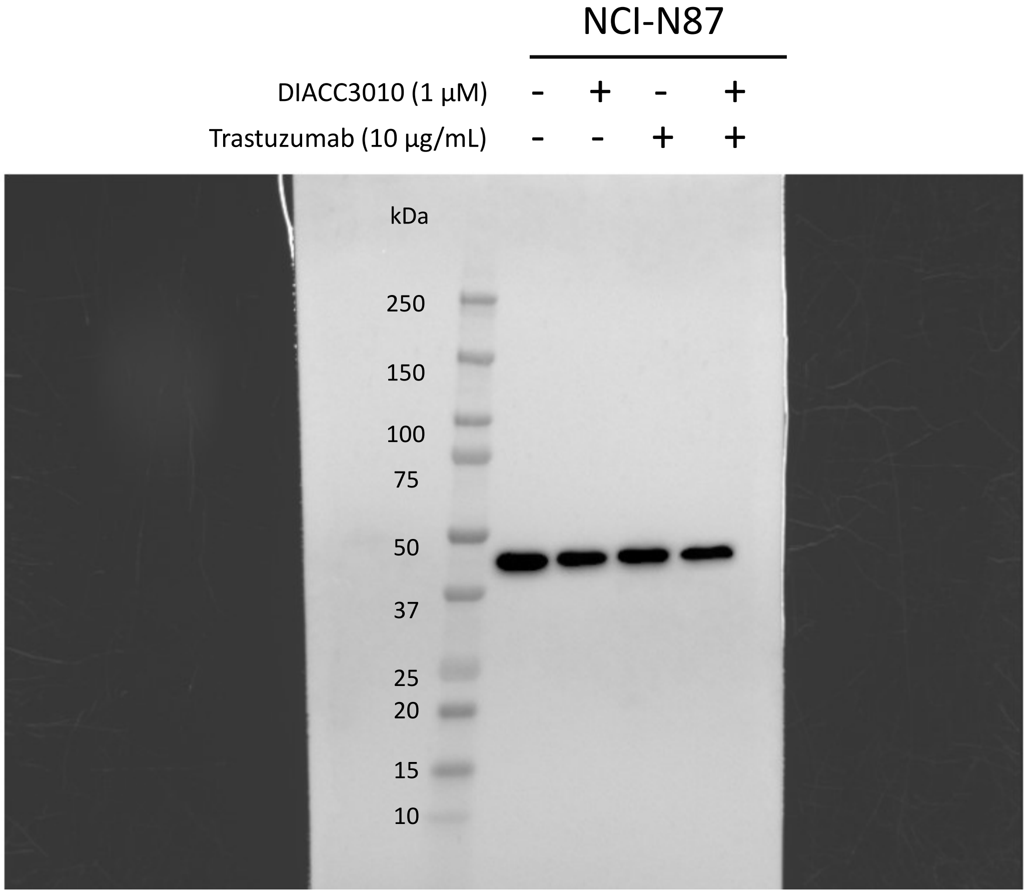

Supplement: Supplementary file 1 — Supplementary Information. [file 41598_2023_40612_MOESM1_ESM.pdf]
